# Gabapentin and pregabalin in bipolar disorder, anxiety states, and insomnia: Systematic review, meta-analysis, and rationale

**DOI:** 10.1038/s41380-021-01386-6

**Published:** 2021-11-24

**Authors:** James S. W. Hong, Lauren Z. Atkinson, Noura Al-Juffali, Amine Awad, John R. Geddes, Elizabeth M. Tunbridge, Paul J. Harrison, Andrea Cipriani

**Affiliations:** 1 Department of Psychiatry, University of Oxford, Warneford Hospital, Oxford, OX3 7JX UK; 2 Oxford Centre for Human Brain Activity, University of Oxford, Oxford, UK; 3 Oxford Health NHS Foundation Trust, Warneford Hospital, Oxford, UK; 4 Department of Neurology, Massachusetts General Hospital, Boston, MA 02114 USA

**Keywords:** Neuroscience, Bipolar disorder

## Abstract

The gabapentinoids, gabapentin, and pregabalin, target the α_2_δ subunits of voltage-gated calcium channels. Initially licensed for pain and seizures, they have become widely prescribed drugs. Many of these uses are off-label for psychiatric indications, and there is increasing concern about their safety, so it is particularly important to have good evidence to justify this usage. We conducted a systematic review and meta-analysis of the evidence for three of their common psychiatric uses: bipolar disorder, anxiety, and insomnia. Fifty-five double-blind randomised controlled trials (RCTs) and 15 open-label studies were identified. For bipolar disorder, four double-blind RCTs investigating gabapentin, and no double-blind RCTs investigating pregabalin, were identified. A quantitative synthesis could not be performed due to heterogeneity in the study population, design and outcome measures. Across the anxiety spectrum, a consistent but not universal effect in favour of gabapentinoids compared to placebo was seen (standardised mean difference [SMD] ranging between -2.25 and -0.25). Notably, pregabalin (SMD -0.55, 95% CI -0.92 to -0.18) and gabapentin (SMD -0.92, 95% CI -1.32 to -0.52) were more effective than placebo in reducing preoperative anxiety. In insomnia, results were inconclusive. We conclude that there is moderate evidence of the efficacy of gabapentinoids in anxiety states, but minimal evidence in bipolar disorder and insomnia and they should be used for these disorders only with strong justification. This recommendation applies despite the attractive pharmacological and genetic rationale for targeting voltage-gated calcium channels.

## Introduction

The gabapentinoids comprise gabapentin and pregabalin. Gabapentin is licensed for use in the USA for the treatment of focal seizures and post-herpetic neuralgia [1] and in the UK for focal seizures and peripheral neuropathic pain [2]. Pregabalin has similar indications, as well as for fibromyalgia in the USA and generalised anxiety disorder (GAD) in the UK [1, 2]. Since their introduction in 1993, these drugs have become two of the most commonly prescribed medications [3], with a significant proportion likely to have been off-label. An early study of 105 Medicaid patients showed that 95% of prescriptions for gabapentin were for off-label indications, at least 10% of which were for psychiatric disorders [4]. In the UK, at least half of all gabapentinoid prescriptions are off-label, and one in five are co-prescribed with opioids [5]. A recent survey using the US-based TriNetX electronic health records network [6] showed that gabapentin had been prescribed at least once in 13.6% of patients with bipolar disorder (BD), 11.5% with anxiety disorders, and 12.7% with insomnia disorder; for pregabalin, the figures were 2.9%, 2.6%, 3.0% respectively (PJH, *unpublished observations*).

Despite the widespread off-label use of gabapentin in BD, the evidence for its efficacy is unclear [7]. Gabapentin was not found to be effective over placebo in a comprehensive network meta-analysis of pharmacologic treatments in acute mania [8]. Systematic reviews of gabapentin treatment in psychiatric and/or substance use disorders showed inconclusive evidence for efficacy in BD, but possible efficacy for some anxiety disorders [9, 10]. These studies did not examine pregabalin, did not attempt a quantitative synthesis, and only included published studies.

Beyond its licensed use for GAD in the UK, pregabalin has been used in the management of other anxiety disorders [11] and acute anxiety states such as preoperative anxiety [12]. The efficacy of pregabalin is well established in GAD [13] but is unclear in other anxiety diagnoses such as social anxiety disorder [14]. A review of pregabalin for preoperative anxiolysis did not attempt a quantitative synthesis [15]. Gabapentinoids may also be of benefit in the treatment of insomnia in some conditions [16], but again the evidence is unclear.

Overall, these studies show that there is limited evidence for the efficacy of gabapentinoids in several of the disorders for which they are used. Moreover, these drugs have potential harms, including a range of serious side-effects, and evidence of misuse and addiction potential [17–20], leading to their re-categorisation as controlled drugs in the UK in 2019. In the United States, pregabalin (but not gabapentin) has been a federally controlled drug since market availability because of its potential for dependence and abuse [21].

A priori, gabapentinoids represent attractive candidate molecules for psychiatric indications because of their mode of action. Despite the nomenclature, they act primarily by inhibiting neuronal voltage-gated calcium channel (VGCC) currents, by binding to the α_2_δ auxiliary subunit that regulates channel trafficking and function [22]. Though genes encoding α_2_δ subunits have not thus far been associated with psychiatric phenotypes, other VGCC genes – particularly *CACNA1C*, that encode the α-1 subunit of the Ca_V_1.2 L-type VGCC subtype – show robust, transdiagnostic associations with multiple disorders, including BD [23–26]. Furthermore, the prescription of calcium channel blockers, which block the α-1 subunit of L-type VGCCs, is associated with reduced hospitalisations in patients with severe mental illness [27]. More broadly, dysregulation of cellular calcium is observed in patients with BD and some other psychiatric disorders [28–30].

Given the potential harms of gabapentinoids, and their widespread off-label prescribing, it is important to assess rigorously the evidence base for their use. Thus, we have carried out a systematic review and meta-analysis of the efficacy, acceptability and tolerability of gabapentin and pregabalin in the treatment of BD, anxiety and insomnia. To complement this analysis, we discuss further the rationale for their use in psychiatric indications.

## Methods

### Search strategy

To identify published studies, we searched the Cochrane Central Register of Controlled Trials (CENTRAL), EMBASE, MEDLINE, MEDLINE In-Process and PsycINFO from their inception to 4^th^ August 2020 (see published protocol [7] and Supplementary Appendix for full information about search terms). For unpublished studies, we searched international trial registries (ClinicalTrials.gov; the International Clinical Trials Registry Platform [ICTRP], www.who.int/clinical-trials-registry-platform/the-ictrp-search-portal) and the websites of the following regulatory agencies: the European Medicines Agency, the United States Food and Drug Administration, the Medicines and Healthcare Products Regulatory Agency in the UK, the Medicines Evaluation Board, The Medical Products Agency, the Pharmaceuticals and Medical Devices Agency, and the Therapeutic Goods Administration in Australia. Reference lists of included studies were hand-checked for relevant papers and systematic reviews.

### Selection criteria

To assess efficacy and acceptability (dropout rate), we included double-blind, randomised controlled trials (DB-RCT) comparing gabapentin or pregabalin, in any dose, frequency, route of administration or setting, with placebo or any other active pharmacological treatment. RCTs investigating adjunctive gabapentin/pregabalin to pre-existing treatment and trials allowing rescue medications were included if pre-existing treatments or rescue medications were evenly distributed in the experimental and comparator intervention arms. To assess tolerability (adverse effects), we included randomised and non-randomised studies, irrespective of blinding. Crossover trials were only included if data from the first period, prior to crossover, were available. Cluster randomised trials were excluded. Patients of any age, sex, ethnicity, and clinical setting were included, but we excluded studies with patients with serious medical illnesses.

For BD, we included studies that assessed both acute treatment (follow-up of 3 weeks for manic and mixed episodes; 8 weeks for depression) and long-term treatment (>12 weeks). We included studies with patients of any BD subtype based on standardised diagnostic criteria, i.e., the International Classification of Diseases (ICD) and the Diagnostic and Statistical Manual of Mental Disorders (DSM). Studies were excluded if a diagnosis of BD was defined using cut-off scores on screening questionnaires, or if patients reported a concurrent Axis I disorder (excluding comorbid anxiety or insomnia) [7].

For anxiety, we included studies of any anxiety disorder as defined in ICD or DSM. We also included studies investigating preoperative anxiety. Studies were excluded if patients reported a concurrent Axis I disorder (excluding BD or insomnia) [7].

For insomnia, we included studies investigating sleep or insomnia, including healthy populations and those with a defined sleep disorder [7].

Three authors (LZA, JSWH, AA) independently screened titles and abstracts generated by the search strategies. Full-text articles were reviewed for inclusion and the relevant information was extracted from included trials. Where both published and unpublished data were available, data from unpublished sources were prioritised in the case of any discrepancies. We contacted authors for additional data if necessary. Graphical data were extracted using WebPlotDigitizer version 4.4 (https://automeris.io/WebPlotDigitizer). The risk of bias for each study was independently assessed by four authors (LZA, JSWH, NA, and AA) using the Cochrane Risk of Bias tool [31]. Any disagreements were resolved by consensus with another team member.

### Outcomes

#### Primary outcome

For the acute treatment of BD, our primary outcome was the efficacy of gabapentin or pregabalin as measured by the following: (i) number of hospital admissions during the study period, (ii) length of hospital admission, (iii) change on validated manic or depressive symptom rating scales from baseline, (iv) change on validated psychotic symptom rating scales from baseline, (v) response to treatment (i.e., at least 50% improvement on any validated rating scale), and (vi) time to cessation of additional treatment for manic/depressive symptoms. For the long-term treatment of BD, our primary outcome was efficacy measured by the following: (i) time to recurrence of any mood episodes, (ii) number of recurrences of any mood episodes during the trial period, and (iii) number of recurrences of manic, mixed, or depressive episodes. For the acute and long-term treatment of anxiety, our primary outcome was efficacy as measured by a change in validated and standardised anxiety rating scales. For the acute and long-term treatment of insomnia, our primary outcome was efficacy measured by the following: (i) objectively measured or self-reported sleep time, (ii) self-reported sleep quality, and (iii) sleep onset latency.

#### Secondary outcomes

Secondary outcomes included the acceptability and tolerability of gabapentin or pregabalin in acute/long-term treatment of BD, anxiety, and insomnia. Acceptability was measured as follows: (i) participants dropping out of treatment due to any cause, (ii) participants dropping out of treatment due to adverse events, and (iii) participants dropping out of treatment due to inefficacy. Tolerability was measured as follows: (i) the number of participants experiencing at least one side effect and (ii) the number of participants experiencing a pre-specified list of side effects in the British National Formulary [2].

### Data analysis

Continuous outcomes were calculated as mean differences (MDs) or standardised MDs (SMDs) with 95% confidence intervals (CI). For studies where the continuous outcome of interest was reported in median/interquartile range (IQR)/range, the method by Wan and colleagues [32] was used to convert values to mean/standard deviation (SD). To deal with the multiplicity of continuous outcome measures, we selected the most relevant outcome guided by previous literature relevant to the diagnostic category, e.g., in GAD, we used the baseline-to-endpoint change in the Hamilton Anxiety Rating Scale (HAM-A) where possible [13]. In preoperative anxiety studies, we used the primary outcome measure of preoperative anxiety recorded at 1–2 h post-drug administration and closest to the time of the induction of anaesthesia. For dichotomous outcomes, risk ratios (RRs) were calculated with 95% CI. For studies with multiple treatment arms of the same type of interventional drug, the mean/SDs were combined following methods described in the Cochrane Handbook (https://training.cochrane.org/handbook/current) and elsewhere [33].

Random effects meta-analyses were conducted, as appropriate, within each disorder [7] as well as across the spectrum of anxiety-related disorders. The grouping of this anxiety spectrum was decided post-hoc to include any disorder classified as an ‘anxiety disorder’ under DSM or ICD classifications, as well as acute anxiety states. Heterogeneity between studies was assessed by visual inspection of forest plots and *I*^2^ statistics. Cases of significant heterogeneity were investigated through subgroup analyses of dose. The following sensitivity analyses were undertaken: (i) excluding trials of gabapentin/pregabalin as adjunctive treatment, (ii) excluding trials involving patients with psychiatric comorbidities, (iii) excluding trials allowing rescue medications and (iv) excluding trials where the outcome measure of interest was originally reported in median/IQR/range. Funnel plots were visually inspected for asymmetry (small study effect) in meta-analyses containing at least 10 studies. Statistical analyses were conducted using Review Manager [34] and R [35]. Data that could not be meta-analysed are presented narratively in a table. The certainty of the evidence was estimated using the Grading of Recommendations, Assessment, Development and Evaluation (GRADE) framework [36]. This study is registered with PROSPERO (registration number CRD42016041802), and we followed the Preferred Reporting Items for Systematic Reviews and Meta-Analyses (PRISMA) guidelines [37].

## Results

Out of 4268 records initially identified, 70 studies were selected for inclusion: 55 DB-RCTs and 15 open-label studies (Fig. 1). One open-label study [38] included an observational component. The risk of bias was unclear for the majority of studies in many domains and a significant proportion of trials were at high risk in terms of attrition bias (Supplementary Appendix, Figs. 1 and 2). A full description of the results is reported in the Supplementary Appendix (Figs. 3–25 and Tables 1–6).

**Fig. 1:**
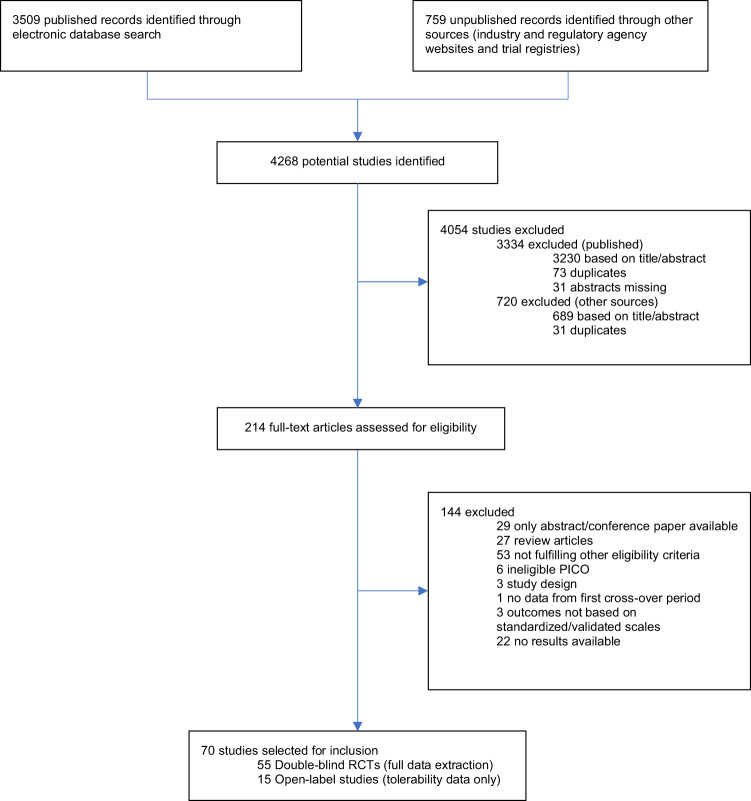
PRISMA diagram. Flowchart of included and excluded studies.

### Bipolar disorder (BD)

Four DB-RCTs investigating the efficacy of gabapentin in BD were identified. 101 patients were randomised to receive gabapentin, 81 to placebo, 30 to lamotrigine and 19 to carbamazepine. The mean age of all randomised patients was 37.5 years and 64.1% were female.

#### Acute treatment

Three studies compared gabapentin with three different comparators for the acute treatment of BD across heterogeneous patient groups with manic/hypomanic, depressed and/or mixed symptoms [39–41] (Table 1). Each study assessed the efficacy of gabapentin using a different outcome measure. Quantitative analysis was not performed due to clinical and methodological heterogeneity.

**Table 1:** Double-blind randomised controlled trials of gabapentin in bipolar disorder (BD).

Study ID	Design	Population	Intervention and Comparator(s)	Placebo control?	Duration of intervention	Primary outcome measure	Key results
**Acute treatment of BD**							
Mokhber 2008 [39]	Parallel	DSM-IV dysphoric mania	Fixed dose gabapentin 900 mg/day (*n* = 18), carbamazepine 600 mg/day (*n* = 13) and lamotrigine 100 mg/day (*n* = 20)	No	8 weeks	MMPI-2	All treatment groups showed a significant within-group improvement in the mania and depression subscales of the MMPI-2 at 8 weeks. Gabapentin showed a significantly greater improvement on the depression symptoms subscale (50%) compared to lamotrigine (33.5%) and carbamazepine (13.6%). There were no significant between-group differences in improvement on the mania symptom subscale. The authors included only study completers in the efficacy analyses.
Frye 2000 [40]	Cross-over	DSM-IV refractory bipolar disorder (BD-I = 11; BD-II = 14) and unipolar depression (UP = 6)	Flexibly dosed gabapentin (mean dose phase 1 = 3987 mg), lamotrigine (274 mg), and placebo	Yes	6 weeks (per phase)	CGI-BP	Response rates (CGI-BP score of much or very much improved) observed during phase 1 was 50% for lamotrigine, 33% for gabapentin and 18% for placebo. Similar clinical response rates were seen across all three phases of the study. Only participants completing all three phases of the study were included in the analysis.
Pande 2000 [41]	Parallel	DSM-IV BD-I with manic/hypomanic (*n* = 82), or mixed symptoms (*n* = 35)	Flexible dose adjunctive gabapentin (*n* = 58) or placebo (*n* = 59) alongside adjunctive treatment with valproate (*n* = 57), lithium (*n* = 39), or both (*n* = 21).	Yes	10 weeks	YMRS and HAM-D	The placebo group showed a significantly greater improvement in the total YMRS change score compared to gabapentin. There was no significant between-group difference in the HAM-D change score. Secondary outcomes showed no significant differences in CGIC response rate or HAM-A change scores between gabapentin and placebo groups, demonstrating no evidence in favor of gabapentin as an adjunctive treatment in BD
**Long-term treatment of BD**							
Vieta 2006 [42]	Parallel	DSM-IV BD-I or BD-II in clinical remission (HAM-D ≤ 8 and YMRS ≤ 4)	Flexible dosed adjunctive gabapentin (*n* = 13) or placebo (*n* = 12)	Yes	1 year	CGI-BP	There was a significant difference in favor of gabapentin in baseline-to-endpoint (1 year) change in CGI-BP score (gabapentin -2.1, placebo -0.6) and in use of sleeping medication (PSQI-item 6, gabapentin -1.1 vs. placebo -0.6). There were no significant differences in YMRS, HAM-D, HAM-A, PSQI change scores, or time to recurrence of new mood episodes between gabapentin and placebo groups.
**Tolerability**							
To further examine the tolerability of gabapentin and pregabalin in BD, 11 open-label studies were included, 8 of which reported outcome data. Among reported side effects, gabapentin was commonly associated with sedation, movement disorders, dyspepsia, drowsiness, dizziness, headache, sleep problems, fatigue, and cognitive side effects. Pregabalin was associated with thought overactivation, weight gain, and increased appetite. All adverse event data are presented in the Supplementary Appendix, Table 6.							
BD-I, bipolar disorder type 1; BD-II, bipolar disorder type 2; CGI-BP, Clinical Global Impressions-Bipolar Version; CGIC, Clinical Global Impression of Change; DSM-IV, Diagnostic and Statistical Manual of Mental Disorders, fourth edition; HAM-A, Hamilton Rating Scale for Anxiety; HAM-D, Hamilton Rating Scale for Depression; MMPI-2, Minnesota Multiphasic Personality Inventory-2; PSQI, Pittsburgh Sleep Quality Index; YMRS, Young Mania Rating Scale.							

Gabapentin was significantly more effective than lamotrigine and carbamazepine in reducing depressive symptoms on the Minnesota Multiphasic Personality Inventory-2 (MMPI-2) depression subscale (50%, 33.5%, and 13.6% reduction, respectively), but there were no group differences in improvements on the MMPI-2 mania subscale [39]. A cross-over study [40] observed response rates of 50% for lamotrigine, 33% for gabapentin, and 18% for placebo on the Clinical Global Impressions-Bipolar Version (CGI-BP) during the first phase, but without clear statistical significance. A third study, testing adjunctive gabapentin vs. placebo, reported a significantly greater improvement in the total Young Mania Rating Scale (YMRS) score in the placebo group but no significant between-group difference in the Hamilton Rating Scale for Depression (HAM-D) change score [41].

#### Long-term treatment

One long-term treatment study of 25 patients with BD in clinical remission (13 on gabapentin, 12 on placebo) [42] showed a significant benefit of gabapentin versus placebo on CGI-BP change scores (gabapentin: -2.1, placebo: -0.6) (Table 1, Supplementary Appendix Tables 1 and 2).

### Anxiety disorders or states

**Fig. 2:**
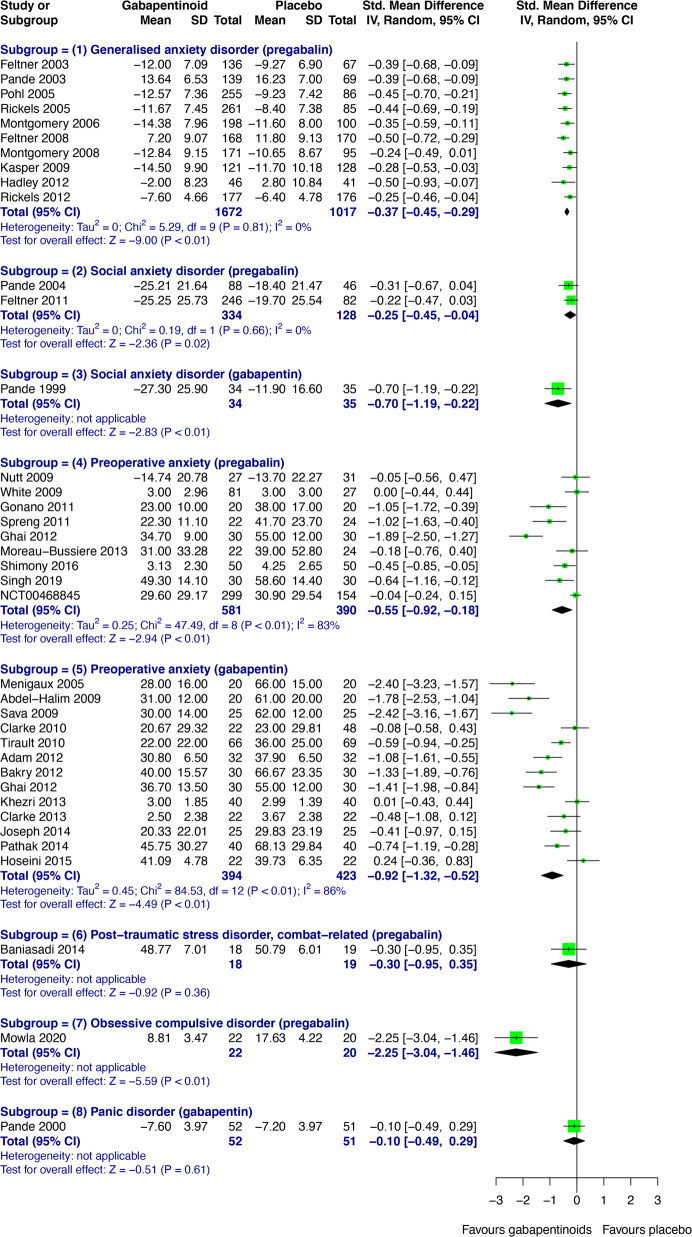
Forest plots showing the efficacy of gabapentinoids versus placebo across the anxiety spectrum. CI Confidence interval, IV Inverse variance, SD Standard deviation.

Forty-two DB-RCTs investigating anxiety disorders/states (GAD; social anxiety disorder, SAD; preoperative anxiety; post-traumatic stress disorder, PTSD; obsessive-compulsive disorder, OCD; panic disorder, PD) were selected for inclusion. 3539 patients were randomised to receive pregabalin, 525 to gabapentin, 2280 to placebo, and 937 to active comparators. The mean age of all randomised patients was 43.0 years, and 60.4% were female. 5190 patients from eligible anxiety studies were analysed for efficacy. Gabapentinoids were significantly more effective than placebo across a range of disorders within the anxiety spectrum (SMDs between -2.25 and -0.25) (Fig. 2). Findings for PTSD, OCD and PD are also presented narratively below, as only one DB-RCT was identified for each of these disorders.

The side effect profile of gabapentinoids was comparable across the anxiety studies. Nineteen studies were included to examine the tolerability of pregabalin in GAD, SAD, PTSD, OCD and PD of that 17 reported side-effect outcomes (Supplementary Appendix, Table 6). Two DB-RCTs reported side-effect outcomes of gabapentin in SAD and PD (Supplementary Appendix, Table 6). For studies of preoperative anxiety, assessment of tolerability was limited due to inconsistent reporting of preoperative side effects across trials. Common side effects of gabapentin and pregabalin included drowsiness, dry mouth, dizziness, headache, fatigue and visual disturbance (see Supplementary Appendix, Table 6).

### Generalised anxiety disorder (GAD)

Pregabalin was significantly more effective than placebo in treating GAD (SMD -0.37, 95% CI -0.45 to -0.29) (Fig. 2). Sensitivity analyses excluding studies in which pregabalin was used as adjunctive treatment, participants had psychiatric comorbidities, or rescue medications were allowed did not significantly alter the effect estimate or estimate of uncertainty (Supplementary Appendix, Table 3). The RR of all-cause drop-out was comparable for pregabalin and placebo. Compared to placebo, pregabalin significantly reduced the RR of drop-out due to inefficacy (RR 0.44, 95% CI 0.28 to 0.70), but showed a trend increase in RR of drop-out due to adverse events (RR 1.30, 95% CI 0.99–1.71) (Supplementary Appendix, Fig. 21). Primary and secondary outcomes relating to the efficacy and acceptability of pregabalin versus lorazepam and venlafaxine are presented in the supplementary analyses (Supplementary Appendix, Figs. 23 and 24). Visual inspection of the funnel plot did not show evidence of small-study effects (Supplementary Appendix, Fig. 6).

### Social anxiety disorder (SAD)

#### Acute treatment

Pregabalin was significantly more effective than placebo in the acute treatment of SAD (SMD -0.25, 95% CI -0.45 to -0.04) (Fig. 2). There was no significant difference in the RR of all-cause drop-out or drop-out due to adverse events (Supplementary Appendix, Figure 8). Compared to placebo, pregabalin showed a trend reduction in RR of drop-out due to inefficacy (RR 0.39, 95% CI 0.15 to 1.03) (Supplementary Appendix, Figure 22).

#### Long-term treatment

A 14-week study of gabapentin in SAD [43] showed significant improvement in symptoms compared to placebo across multiple clinician-evaluated and self-report rating scales. A 26-week study [44] investigating pregabalin for relapse prevention found that a fixed dose of 450 mg/day significantly reduced the overall relapse rate compared to placebo (27.5% vs. 43.8%, based on CGI-Improvement/CGI-Severity criteria).

### Preoperative anxiety

**Fig. 3:**
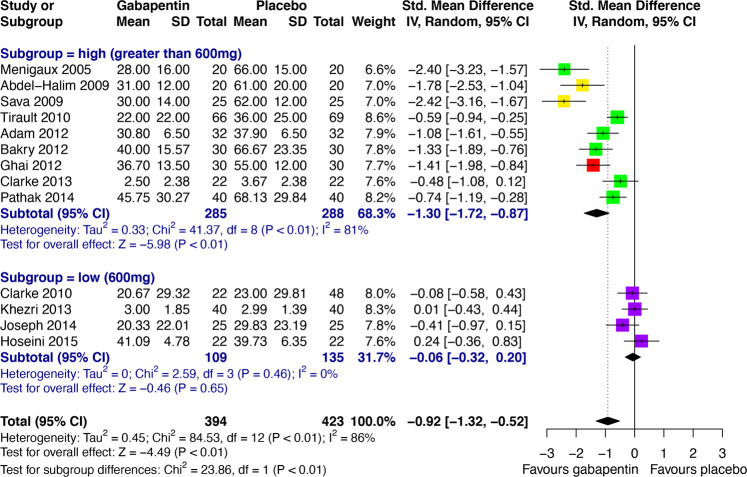
Subgroup analysis of empirically guided high (>600 mg; including 1200 mg (green), 900 mg (red) and 800 mg (yellow)) versus low doses (600 mg, purple) of gabapentin vs. placebo [46] in preoperative anxiety. CI Confidence interval, IV Inverse variance, SD Standard deviation.

**Fig. 4:**
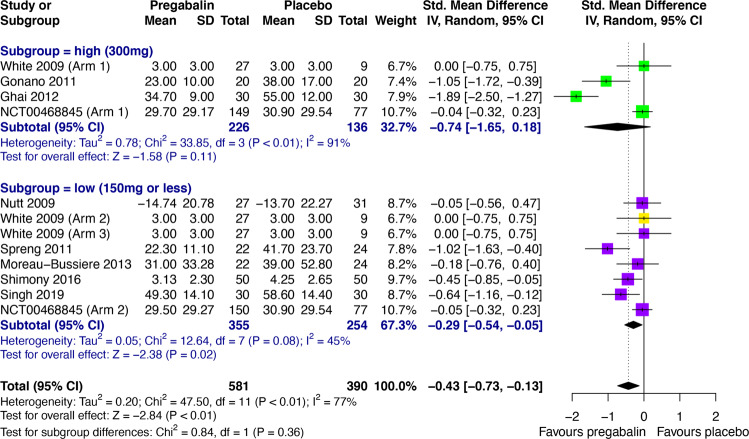
Subgroup analysis of empirically guided high (300 mg, green) versus low doses (≤150 mg; including 75 mg (yellow) and 150 mg (purple)) of pregabalin vs. placebo [45] in preoperative anxiety. CI Confidence interval, IV Inverse variance, SD Standard deviation.

Compared with placebo, both pregabalin (SMD -0.55, 95% CI -0.92 to -0.18) and gabapentin (SMD -0.92, 95% CI -1.32 to -0.52) were significantly more effective in reducing preoperative anxiety (Fig. 2). Significant heterogeneity was found between these studies, which was investigated post-hoc through subgroup analyses based on empirically guided dose thresholds of dose-dependent efficacy [45, 46]. High doses (>600 mg) of gabapentin were found to be significantly effective in reducing preoperative anxiety (SMD -1.30, 95% CI -1.72 to -0.87), but low doses (600 mg) were not (Fig. 3). A high degree of heterogeneity across studies in the high dose group remained (*I*^*2*^ = 81%), hence the validity of the high dose effect estimate is uncertain. Visual inspection of a funnel plot for gabapentin versus placebo in preoperative anxiety suggested possible small study effects, with greater effect size being observed in high-dose gabapentin studies with smaller samples (Supplementary Appendix, Fig. 5.1). There was no significant subgroup difference between low (≤150 mg) and high (300 mg) doses of pregabalin (Fig. 4). Interestingly, low-dose pregabalin was significantly more effective than placebo (SMD -0.29, 95% CI -0.54 to -0.05), whilst the high dose was not. It is worth noting that the splitting of patients in the placebo arm across the intervention arms, to facilitate dose-related subgroup analysis, resulted in a slightly reduced overall effect estimate (SMD -0.43, 95% CI -0.73 to -0.13) (Fig. 4) compared with the original analysis (Fig. 2), but the overall effect remained significant. Sensitivity analyses did not significantly alter the results (Supplementary Appendix, Tables 4 and 5). It was not feasible to perform meta-analyses for the acceptability of pregabalin and gabapentin in preoperative anxiety studies. Short outcome assessment times (lasting hours rather than weeks) meant that dropouts occurred in very few studies. In such cases, dropouts were due to practicalities of preoperative procedures and not due to participant decisions.

### PTSD, OCD, and PD

Three DB-RCTs were included, two assessing adjunctive pregabalin for the treatment of PTSD [47] and OCD [48] and one assessing gabapentin for the treatment of PD [49] (also see Supplementary Appendix, Table 2). Quantitative synthesis was not conducted due to clinical and methodological heterogeneity. Adjunctive pregabalin was more effective than placebo in combat-related chronic PTSD (significantly reduced the severity of symptoms on the PTSD checklist military version at 6 weeks, but not HAM-A, HAM-D, or Spitzer Quality of Life Index) and SSRI-resistant OCD (significantly reduced score on the Yale-Brown Obsessive-Compulsive Scale at 12 weeks). However, eight weeks of flexibly dosed gabapentin was not more effective than placebo in patients with PD (comparable baseline-to-endpoint change on the Panic and Agoraphobia Scale).

### Insomnia/Sleep disturbance

Eight DB-RCTs assessed the efficacy of gabapentin on sleep-related outcomes. Of two trials with sufficient data to conduct a random-effects meta-analysis [50, 51], 111 patients were randomized to receive gabapentin and 60 to placebo (mean age 44.8 years; 44.9% female). In participants with alcohol dependence and related sleep disturbance, gabapentin did not demonstrate improvement in sleep scales compared to placebo (Supplementary Appendix, Figs. 12 and 13). The RR for all-cause drop-out was comparable for gabapentin and placebo (RR 0.96, 95% CI 0.59–1.57) (Supplementary Appendix, Fig. 25). Dropouts due to adverse events and inefficacy could not be assessed as there was no incidence of dropouts contributing to either outcome. Side effect data are reported in the supplementary material (Supplementary Appendix, Table 6).

Just one DB-RCT [52] assessed the efficacy of pregabalin (*n* = 121) vs. venlafaxine (*n* = 125) and placebo (*n* = 128) on sleep-related outcomes (mean age of all randomised patients 40.8 years, 60.7% female). Pregabalin significantly reduced scores on the Medical outcomes study sleep scale and sleep problems index II compared with placebo at weeks 4 and 8.

## Discussion

We conducted a systematic review on the efficacy, acceptability and tolerability of gabapentinoids in BD, anxiety, and insomnia/sleep disturbance. By comparison with prior studies, our systematic review covered more disorders and databases, considered only DB-RCTs for evidence synthesis on efficacy, included unpublished literature, and, as far as possible, conducted quantitative syntheses. Our study shows that there is minimal evidence to support the use of gabapentinoids in BD and insomnia. The moderate effect size was seen across the anxiety spectrum; this was also significant for several individual anxiety states and showed a dose-effect of gabapentin in preoperative anxiolysis.

In BD, the small number of DB-RCTs investigating gabapentin, and the absence of studies investigating pregabalin, highlight the sparse evidence based on that to evaluate the efficacy of gabapentinoids in the treatment of BD. Quantitative synthesis was not performed due to heterogeneity in study population, design, and outcome measures. The evidence is inconclusive and does not support the current use of gabapentinoids in the management of BD.

Our analysis of all anxiety-related studies showed statistically significant low to large effect sizes favoring gabapentinoid use, compared to placebo, across the spectrum of anxiety disorders/states. This transdiagnostic effect is supported by the fact that state, trait, and pathological anxiety are mediated by a common brain network [53, 54] and by the common neural phenotypes observed across anxiety disorders [55]. Our approach necessitated the inclusion of baseline-to-endpoint change scores as well as post-intervention scores, but a study of 21 meta-analyses found that combining post-intervention and change scores produces valid meta-analytical results [56]. The results suggest a dose-dependent effect of preoperative gabapentin, with >600 mg being required for significant acute anxiolysis. These analyses were performed post-hoc to investigate the large between-study heterogeneity and therefore should be considered as hypothesis-generating to guide future studies. The choice of dose thresholds was based on empirical reports of dose-dependent effects in GAD, which is a limitation of this subgroup analysis. For gabapentin, a dose-response pattern has been observed in GAD with remission/mild anxiety on total daily doses of gabapentin ≥900 mg/day and recurrence of severe anxiety, suggesting ineffectiveness, at <600 mg/day [46]. For pregabalin, we used a similar approach based on a reported difference in efficacy between 150 mg/day versus 200–600 mg/day in GAD [45].

Our meta-analysis of studies of alcohol-related insomnia found that gabapentin was not significantly better than placebo in improving self-reported sleep quality. One relevant study [50] of alcohol-related insomnia reported total sleep time and sleep onset latency but did not show a significant benefit of gabapentin over placebo on these outcome measures. Two studies of gabapentin versus placebo in healthy adults with occasional sleep disturbance [57, 58] suggest a limited benefit in this population for sleep quality and total sleep time. Gabapentinoids may improve sleep in patients with a range of clinical conditions, such as GAD- and neuropathic pain-associated insomnia [59], but the extent to which sleep parameters are affected by direct or indirect effects in these conditions is unclear. Whilst studies of pregabalin versus placebo in GAD included in our review reported improvements in insomnia subscales of HAM-A, these were not meta-analyzed due to concerns regarding confounding and the validity of HAM-A subscales in insomnia.

The limited evidence for the efficacy of gabapentinoids in several of the conditions we studied here is complemented by good evidence of their side effects and potential harms. Consistent with previous reports [1], our findings show that gabapentinoids commonly cause side effects associated with central nervous system depression, such as drowsiness and dizziness, which may be associated with an increased risk of accidental physical injuries and road traffic incidents [20]. There is also increasing concern about the addictive potential of gabapentinoids, and the harms when used concurrently with opioids [17, 60]. Another important limitation of the retrieved evidence is the methodological quality and the risk of bias of the included studies. The prominent side-effect profiles of gabapentinoids [61] may be associated with difficulties in maintaining the blinding of patients and participants, and this may have led to the overestimation of effect sizes based on subjective outcomes [62]. Therefore, trial results suggesting gabapentinoid efficacy should be interpreted with caution, particularly when such findings form the basis for their use in unlicensed indications. Against this background, the extensive off-label usage of gabapentinoids appears unwarranted and requires closer scrutiny.

### The rationale for α_2_δ ligands in psychiatry

Neurobiological and pharmacological considerations supporting the candidacy of a drug target should never trump the empirical clinical evidence regarding efficacy and safety. Nevertheless, they may be useful when evaluating the potential for further investigations of the target where evidence is limited.

As outlined earlier, a role for VGCCs in psychiatric disorders is supported by genomic data. The evidence includes both common and rare variants and is most robust for schizophrenia [25] and BD [26], but VGCC associations are observed across a range of psychiatric disorders [63], albeit not (yet) for anxiety disorders [64]. However, the genetic associations to VGCCs are primarily with α-1 and β subunits; we are unaware of robust evidence directly implicating α_2_δ subunit genes.

Furthermore, the desired nature and direction of the manipulation required for therapeutic benefit remains to be determined. Whilst the existing VGCC data are based on channel blockade [27, 65], alternative and more nuanced approaches are likely to be important. For example, VGCC genes encode multiple isoforms [66] with different properties, including sensitivity to the existing channel blockers [67], and with differential expression between tissues [68]. The impact of rare mutations also hints at the need to modulate, rather than simply block, VGCC function in psychiatric disorders. In the case of *CACNA1C*, for example, gain-of-function mutations cause Timothy Syndrome, in which autism is a cardinal feature [69], but autism has also been reported as a feature with loss-of-function mutations [70]. The latter findings are consistent with the presence of psychiatry-relevant phenotypes in *Cacna1c* heterozygous rats [71], which have reduced gene dosage. Taken together, these findings highlight the need for therapeutic agents that are capable of fine-tuning function, perhaps by targeting specific isoforms, or acting homeostatically, in order to maximize clinical benefit and minimize side effects.

Targetting the α_2_δ subunits, as the gabapentinoids do, provides a potential means to achieve nuanced modulation of VGCC function. α_2_δ subunits increase the density of VGCCs on the plasma membrane, direct trafficking of these channels to subcellular sites, and enhance function by altering their biophysical properties [72]. Conversely, gabapentin decreases the number of α_2_δ and α-1 subunits on the cell surface [73] and reduces VGCC currents [74], suggesting an inhibitory role. However, the precise effect of gabapentin on calcium currents depends on the stoichiometry of VGCC auxiliary subunits [74] which, like the other VGCC subunits, differ in abundance between tissues, raising the possibility that gabapentinoids may differentially affect VGCC currents in different cell types. Furthermore, it is possible that the gabapentinoids’ effects on anxiety are mediated by α_2_δ-dependent, but VGCC-independent, mechanisms [75]. Notably, α_2_δ-1 interacts with NMDA receptors (NMDARs) to promote dendritic spine maturation and NMDAR trafficking to synaptic sites [76]. Thus, as well as clarifying the clinical effects of the gabapentinoids, it will be of interest to establish the underlying molecular mechanisms in order to illuminate pathophysiology and identify novel therapeutic strategies. Ultimately, a personalized medicine approach may well be appropriate [77], given the genomic and other contributions to VGCC function and involvement in psychiatric disorders [78].

### Future directions for clinical research: Targeted treatment of anxiety in BD

Gabapentinoids, as shown here, are broadly efficacious across the anxiety spectrum, and it is likely that this reflects a pharmacologic effect on transdiagnostic anxiety phenotypes mediated by α_2_δ-dependent mechanisms. Anxiety is the most common co-morbidity in patients with BD [79], reflecting in part a shared genetic predisposition [80]. It is also associated with a greater symptom burden and a range of worse clinical outcomes [81]. However, there are to date no clinical trials investigating the efficacy of gabapentinoids in the treatment of anxiety in BD. Therefore, a future area of research would be to explore the targeted treatment of ‘bipolar anxiety’ using gabapentinoids or modified α_2_δ ligands.

## Conclusions

The results of this systematic review and meta-analysis show that the widespread and often off-label psychiatric prescribing of gabapentinoids is not supported by robust evidence except for some anxiety states. Thus, despite the attractive genetic and pharmacological rationale for their use, caution is indicated, and further evidence of efficacy and safety is required. It may also be possible to develop modified α_2_δ ligands, targeting particular subtypes or isoforms, with a more beneficial therapeutic profile.

## Supplementary information

Supplementary Appendix
