# Supplementary material for: Gabapentin and pregabalin in bipolar disorder, anxiety states, and insomnia: Systematic review, meta-analysis, and rationale
Source: Mol Psychiatry. 2021 Nov 24;27(3):1339–49. doi: 10.1038/s41380-021-01386-6 (PMC9095464; doi:10.1038/s41380-021-01386-6)
Supplement: Supplementary file 1 — Supplementary Appendix [file 41380_2021_1386_MOESM1_ESM.docx]

**Supplementary appendix**

**Title**

Gabapentin and pregabalin in bipolar disorder, anxiety states, and insomnia: systematic review, meta-analysis, and rationale

**Authors**

James S W Hong, Lauren Z Atkinson, Noura Al-Juffali, Amine Awad, John R Geddes, Elizabeth M Tunbridge, Paul J Harrison, Andrea Cipriani

Table of Contents

[Search Terms 4](#_Toc86053037)

[Reference list of included studies 5](#_Toc86053038)

[DB-RCT (k = 55) 5](#_Toc86053039)

[Open label studies (k = 15) 11](#_Toc86053040)

[Table 1. Characteristics of all diagnostic groups 13](#_Toc86053041)

[Table 2. Characteristics of included double-blind randomised controlled studies 15](#_Toc86053042)

[Table 4. Sensitivity analyses of main text Figure 3: Gabapentin vs placebo in preoperative anxiety by dose subgroups 26](#_Toc86053043)

[Table 5. Sensitivity analyses of main text Figure 4: Pregabalin vs placebo in preoperative anxiety by dose subgroups 27](#_Toc86053044)

[Table 6. Tolerability (table of side effects) 28](#_Toc86053045)

[Risk of bias of included studies 48](#_Toc86053046)

[Figure 1. Risk of bias summary 48](#_Toc86053047)

[Figure 2. Risk of bias by individual study. 49](#_Toc86053048)

[50](#_Toc86053049)

[Primary outcome: efficacy, forest plots, including sensitivity analyses 51](#_Toc86053050)

[Figure 3: Pregabalin vs placebo in a meta-analysis of generalised anxiety disorder, social anxiety disorder, pre-operative anxiety, PTSD, and OCD 51](#_Toc86053051)

[Figure 4: Gabapentin vs placebo in a meta-analysis of pre-operative anxiety, social anxiety disorder, and panic disorder 52](#_Toc86053052)

[Figure 5: Pregabalin vs placebo in generalised anxiety disorder, Mean difference in HAM-A* 53](#_Toc86053053)

[Figure 6: Funnel Plot - Pregabalin vs placebo in generalised anxiety disorder (GAD), Mean difference in HAM-A 53](#_Toc86053054)

[Figure 7: Pregabalin vs lorazepam in generalised anxiety disorder, Mean difference in HAM-A 54](#_Toc86053055)

[Figure 8: Lorazepam vs placebo in generalised anxiety disorder, Mean difference in HAM-A 54](#_Toc86053056)

[Figure 9: Pregabalin vs venlafaxine in generalised anxiety disorder, Mean difference in HAM-A 54](#_Toc86053057)

[Figure 10: Venlafaxine vs placebo in generalised anxiety disorder, Mean difference in HAM-A 54](#_Toc86053058)

[Figure 11: Funnel plot (gabapentin versus placebo in preoperative anxiety) 55](#_Toc86053059)

[Figure 12: Gabapentin vs placebo in insomnia participants with alcohol dependence, Week 6, SMD (standardised mean difference) 56](#_Toc86053060)

[Figure 13: Gabapentin vs placebo in insomnia participants with alcohol dependence, Week 12, SMD (standardised mean difference) 56](#_Toc86053061)

[Figure 14: Gabapentin vs placebo, weeks 4-6, subjective measures, including healthy volunteers, SMD (standardised mean difference) 56](#_Toc86053062)

[Figure 15: Gabapentin vs placebo, week 12, subjective measures, including healthy volunteers, SMD (standardised mean difference) 57](#_Toc86053063)

[Figure 16: Gabapentin vs placebo, weeks 1-4, Mean difference in PSG-derived WASO*, including healthy volunteers 57](#_Toc86053064)

[Figure 17: Pregabalin vs placebo in social anxiety disorder, Mean difference in LSAS* 57](#_Toc86053065)

[Figure 18: Pregabalin vs placebo in social anxiety disorder, Mean difference in HAM-A* 58](#_Toc86053066)

[Figure 19: Pregabalin vs placebo in social anxiety disorder, Mean difference in HAM-D* 58](#_Toc86053067)

[Figure 20: Pregabalin vs placebo in social anxiety disorder, Mean difference in MFQ* 58](#_Toc86053068)

[Secondary outcome: acceptability, forest plots 59](#_Toc86053069)

[Figure 21: Acceptability of pregabalin versus placebo in GAD 59](#_Toc86053070)

[Figure 22: Acceptability of pregabalin versus placebo in SAD 60](#_Toc86053071)

[Supplementary Analyses 61](#_Toc86053072)

[Figure 23: Acceptability of pregabalin versus lorazepam in GAD 62](#_Toc86053073)

[Figure 24: Acceptability of pregabalin versus venlafaxine in GAD 63](#_Toc86053074)

[Figure 25: Acceptability of gabapentin versus placebo in participants with alcohol dependence and related sleep disturbance 64](#_Toc86053075)

[Further details of tolerability data 65](#_Toc86053076)

[Bipolar disorder (BD) 65](#_Toc86053077)

[Generalised anxiety disorder (GAD) and Social anxiety disorder (SAD) 65](#_Toc86053078)

[Preoperative anxiety 65](#_Toc86053079)

[Insomnia 65](#_Toc86053080)

[Changes to original protocol 67](#_Toc86053081)

# **Search Terms**

The following phrase was used in literature searches:

((((‘bipolar disorder’ OR ‘cyclothymic disorder’) OR ‘sleep initiation and maintenance disorders’ OR (‘anxiety’ OR ‘anxiety disorders’ OR ‘agoraphobia’ OR ‘anxiety, separation’ OR ‘combat disorders’ OR ‘neurotic disorders’ OR ‘obsessive-compulsive disorder’ OR ‘panic disorder’ OR ‘phobic disorders’ OR ‘stress disorders, traumatic’ OR ‘stress disorders, post-traumatic’ OR ‘psychological trauma’ OR ‘stress disorders, traumatic, acute’)) AND (‘gamma-aminobutyric acid’ OR ‘pregabalin’)) combined with terms for randomised controlled trials (efficacy) OR adverse effects (tolerability))*

**Subject (MeSH) entries sourced from MEDLINE*

# Reference list of included studies

## DB-RCT (k = 55)

**Bipolar disorder**

**Frye 2000 (NCT00001482)**

- Frye MA, Ketter TA, Kimbrell TA, Dunn RT, Speer AM, Osuch EA et al. A placebo-controlled study of lamotrigine and gabapentin monotherapy in refractory mood disorders. *J Clin Psychopharmacol* 2000; 20(6): 607-14. doi: 10.1097/00004714-200012000-00004.

**Mokhber 2008**

- Mokhber N, Lane CJ, Azarpazhooh MR, Salari E, Fayazi R, Shakeri MT et al. Anticonvulsant treatments of dysphoric mania: a trial of gabapentin, lamotrigine and carbamazepine in Iran. *Neuropsychiatr Dis Treat* 2008; 4(1): 227-34. doi: 10.2147/ndt.s2316.

**Pande 2000a** **(945-209)**

- Pande AC, Crockatt JG, Janney CA, Werth JL, Tsaroucha G. Gabapentin in bipolar disorder: a placebo-controlled trial of adjunctive therapy. Gabapentin Bipolar Disorder Study Group. *Bipolar Disord* 2000; 2(3 Pt 2): 249-55. doi: 10.1034/j.1399-5618.2000.20305.x.

**Vieta 2006 (945-291)**

- Vieta E, Manuel Goikolea J, Martínez-Arán A, Comes M, Verger K, Masramon X et al. A double-blind, randomized, placebo-controlled, prophylaxis study of adjunctive gabapentin for bipolar disorder. *J Clin Psychiatry* 2006; 67(3): 473-7. doi: 10.4088/jcp.v67n0320.

**Pre-operative studies**

**Abdel-Halim 2009**

- Abdel-Halim JMK, ElAwady GA, ElShaikh SM, Azer MS. Gabapentin and dexamethasone as adjuvants for intraoperative and postoperative pain management. *Eg J Anaesth* 2009; 25(4): 347-354.

**Adam 2012**

- Adam F, Bordenave L, Sessler DI, Chauvin M. Effects of a single 1200-mg preoperative dose of gabapentin on anxiety and memory. *Ann Fr Anesth Reanim* 2012; 31(10): e223-7. doi: 10.1016/j.annfar.2012.05.006.

**Bakry 2012**

- Bakry AEAE, Marey H. The effect of gabapentin premedication on pain and anxiety during cataract surgery under peribulbar block.*Egyptian Journal of Anaesthesia* 2012; 28(1): 13-47. doi: [10.1016/j.egja.2011.10.001](https://doi.org/10.1016/j.egja.2011.10.001).

**Clarke 2010**

- Clarke H, Kay J, Orser BA, Gollish J, Mitsakakis N, Katz J. Gabapentin does not reduce preoperative anxiety when given prior to total hip arthroplasty. *Pain Med* 2010; 11(6): 966-71. doi: 10.1111/j.1526-4637.2010.00826.x.

**Clarke 2013**

- Clarke H, Kirkham KR, Orser BA, Katznelson R, Mitsakakis N, Ko R et al. Gabapentin reduces preoperative anxiety and pain catastrophizing in highly anxious patients prior to major surgery: a blinded randomized placebo-controlled trial. *Can J Anaesth* 2013; 60(5): 432-43. doi: 10.1007/s12630-013-9890-1.

**Ghai 2012**

- Ghai A, Gupta M, Rana N, Wadhera R. The effect of pregabalin and gabapentin on preoperative anxiety and sedation: a double blind study. *Anaesth Pain & Intensive Care* 2012; 16(3): 257-261.

**Gonano 2011**

- Gonano C, Latzke D, Sabeti-Aschraf M, Kettner SC, Chiari A, Gustorff. The anxiolytic effect of pregabalin in outpatients undergoing minor orthopaedic surgery. *J Psychopharmacol* 2011; 25(2): 249-253. doi: 10.1177/0269881109106928.

**Hoseini 2015** (IRCT2014041217231N1)

- Hoseini VS, Yekta RA, Marashi S, Marashi SM. The efficacy of melatonin, clonidine and gabapentin in reducing preoperative anxiety and postoperative pain in patients undergoing laparoscopic cholecystectomy: a randomized clinical trial. *Arch Anesth & Critical Care* 2015; 1(4): 120-125.

**Joseph 2014 (CTRI/2010/091/002830)**

- Joseph TT, Krishna HM, Kamath S. Premedication with gabapentin, alprazolam or a placebo for abdominal hysterectomy: Effect on pre-operative anxiety, post-operative pain and morphine consumption. *Indian J Anaesth* 2014; 58(6): 693-9. doi: 10.4103/0019-5049.147134.

**Khezri 2013 (ACTRN12610000727044, NCT01200641)**

- Khezri MB, Oladi MR, Atlasbaf A. Effect of melatonin and gabapentin on anxiety and pain associated with retrobulbar eye block for cataract surgery: a randomized double-blind study. *Indian J Pharmacol*. 2013; 45(6): 581-6. doi: 10.4103/0253-7613.121368.

**Menigaux 2005**

- Ménigaux C, Adam F, Guignard B, Sessler DI, Chauvin M. Preoperative gabapentin decreases anxiety and improves early functional recovery from knee surgery. *Anesth Analg* 2005; 100(5): 1394-9. doi: 10.1213/01.ANE.0000152010.74739.B8.

**Moreau-Bussière 2013 (NCT01158859)**

- Moreau-Bussière F, Gaulin J, Gagnon V, Sansoucy Y, de Médicis E. Preoperative pregabalin does not reduce propofol ED(50): a randomized controlled trial. *Can J Anaesth* 2013; 60(4): 364-9. doi: 10.1007/s12630-013-9885-y.

**Nasr 2014**

- Nasr DA, Abdellatif AA. Efficacy of preoperative melatonin versus pregabalin on perioperative anxiety and postoperative pain in gynecological surgeries. *Egypt J Anaesth* 2014; 30(1): 89-93.

**NCT00468845**

- NCT00468845. Study of the efficacy and safety of pregabalin compared to placebo for treatment of post-surgical pain from hysterectomy. <https://clinicaltrials.gov/ct2/show/NCT00468845> (first received 3 May 2007).

**NCT00551135**

- NCT00551135. Surgical pain after inguinal hernia repair (SPAIHR). <https://www.clinicaltrials.gov/ct2/show/NCT00551135> (first received 30 October 2007).

**Nutt 2009**

- Nutt D, Mandel F, Baldinetti F. Early onset anxiolytic efficacy after a single dose of pregabalin: double-blind, placebo- and active-comparator controlled evaluation using a dental anxiety model. *J Psychopharmacol* 2009; 23(8): 867-73. doi: 10.1177/0269881108094722.

**Pathak 2014**

- Pathak L, Chaturvedi A. Effect of gabapentin premedication on preoperative anxiety and postoperative pain. *Health Renaissance* 2014; 11(3): 254-259. <https://doi.org/10.3126/hren.v11i3.9642>

**Rorarius 2004**

- Rorarius MG, Mennander S, Suominen P, Rintala S, Puura A, Pirhonen R et al. Gabapentin for the prevention of postoperative pain after vaginal hysterectomy. *Pain* 2004; 110(1-2): 175-81. doi: 10.1016/j.pain.2004.03.023.

**Sava 2009**

- Sava M, Rusu N. Effects of gabapentin on preoperative anxiety and postoperative analgesia with morphine in rectocolic surgery. *Jurnalul Roman de Anestezie Terapie Intensiva/Romanian Journal of Anaesthesia and Intensive Care* 2009; 16(1): 10-16.

**Shimony 2016 (NCT01612832)**

- Shimony N, Amit U, Minz B, Grossman R, Dany MA, Gonen L et al. Perioperative pregabalin for reducing pain, analgesic consumption, and anxiety and enhancing sleep quality in elective neurosurgical patients: a prospective, randomized, double-blind, and controlled clinical study. *J Neurosurg* 2016; 125(6): 1513-1522. doi: 10.3171/2015.10.JNS151516.

**Singh 2019**

- Singh D, Yadav JS, Jamuda BK, Singh P. Oral Pregabalin as Premedication on Anxiolysis and Stress Response to Laryngoscopy and Endotracheal Intubation in Patients Undergoing Laparoscopic Cholecystectomy: A Randomized Double-Blind Study. *Anesth Essays Res* 2019; 13(1): 97-104. doi: 10.4103/aer.AER_12_19.

**Spreng 2011 (NCT00353704)**

- Spreng UJ, Dahl V, Raeder J. Effect of a single dose of pregabalin on post-operative pain and pre-operative anxiety in patients undergoing discectomy. *Acta Anaesthesiol Scand* 2011; 55(5): 571-6. doi: 10.1111/j.1399-6576.2011.02410.x.

**Tirault 2010**

- Tirault M, Foucan L, Debaene B, Frasca D, Lebrun T, Bernard JC et al. Gabapentin premedication: assessment of preoperative anxiolysis and postoperative patient satisfaction. *Acta Anaesthesiol Belg* 2010; 61(4): 203-9.

**White 2009**

- White PF, Tufanogullari B, Taylor J, Klein K. The effect of pregabalin on preoperative anxiety and sedation levels: a dose-ranging study. *Anesth Analg* 2009; 108(4): 1140-5. doi: 10.1213/ane.0b013e31818d40ce.

**Generalised anxiety disorder (GAD)**

**Feltner 2003**

- Feltner DE, Crockatt JG, Dubovsky SJ, Cohn CK, Shrivastava RK, Targum SD et al. A randomized, double-blind, placebo-controlled, fixed-dose, multicenter study of pregabalin in patients with generalized anxiety disorder. *J Clin Psychopharmacol* 2003; 23(3): 240-9. doi: 10.1097/01.jcp.0000084032.22282.ff.

**Feltner 2008**

- Feltner D, Wittchen HU, Kavoussi R, Brock J, Baldinetti F, Pande AC. Long-term efficacy of pregabalin in generalized anxiety disorder. *Int Clin Psychopharmacol* 2008; 23(1): 18-28. doi: 10.1097/YIC.0b013e3282f0f0d7.

**Hadley 2012 (NCT00368745, 2006-001347-66)**

- Hadley SJ, Mandel FS, Schweizer E. Switching from long-term benzodiazepine therapy to pregabalin in patients with generalized anxiety disorder: a double-blind, placebo-controlled trial. *J Psychopharmacol* 2012; 26(4): 461-70. doi: 10.1177/0269881111405360.

**Kasper 2009 (NCT00151450, EUCTR2004-001500-13-IE)**

- Kasper S, Herman B, Nivoli G, Van Ameringen M, Petralia A, Mandel FS et al. Efficacy of pregabalin and venlafaxine-XR in generalized anxiety disorder: results of a double-blind, placebo-controlled 8-week trial. *Int Clin Psychopharmacol* 2009; 24(2): 87-96. doi: 10.1097/yic.0b013e32831d7980.

**Kasper 2014** **(NCT00624780, EUCTR2007-004768-32-ES)**

- Kasper S, Iglesias-García C, Schweizer E, Wilson J, DuBrava S, Prieto R et al. Pregabalin long-term treatment and assessment of discontinuation in patients with generalized anxiety disorder. *Int J Neuropsychopharmacol* 2014; 17(5): 685-95. doi: 10.1017/S1461145713001557.

**Montgomery 2006**

- Montgomery SA, Tobias K, Zornberg GL, Kasper S, Pande AC. Efficacy and safety of pregabalin in the treatment of generalized anxiety disorder: a 6-week, multicenter, randomized, double-blind, placebo-controlled comparison of pregabalin and venlafaxine. *J Clin Psychiatry* 2006; 67(5): 771-82. doi: 10.4088/jcp.v67n0511.

**Montgomery 2008 (EUCTR2004-000955-40-LV)**

- Montgomery S, Chatamra K, Pauer L, Whalen E, Baldinetti F. Efficacy and safety of pregabalin in elderly people with generalised anxiety disorder. *Br J Psychiatry* 2008; 193(5): 389-94. doi: 10.1192/bjp.bp.107.037788.

**Pande 2003**

- Pande AC, Crockatt JG, Feltner DE, Janney CA, Smith WT, Weisler R et al. Pregabalin in generalized anxiety disorder: a placebo-controlled trial. *Am J Psychiatry* 2003; 160(3): 533-40. doi: 10.1176/appi.ajp.160.3.533.

**Pohl 2005**

- Pohl RB, Feltner DE, Fieve RR, Pande AC. Efficacy of pregabalin in the treatment of generalized anxiety disorder: double-blind, placebo-controlled comparison of BID versus TID dosing. *J Clin Psychopharmacol* 2005; 25(2): 151-8. doi: 10.1097/01.jcp.0000155820.74832.b0.

**Rickels 2005**

- Rickels K, Pollack MH, Feltner DE, Lydiard RB, Zimbroff DL, Bielski RJ et al. Pregabalin for treatment of generalized anxiety disorder: a 4-week, multicenter, double-blind, placebo-controlled trial of pregabalin and alprazolam. *Arch Gen Psychiatry* 2005; 62(9): 1022-30. doi: 10.1001/archpsyc.62.9.1022.

**Rickels 2012 (NCT00413010, EUCTR2006-006339-31-FI)**

- Rickels K, Shiovitz TM, Ramey TS, Weaver JJ, Knapp LE, Miceli JJ. Adjunctive therapy with pregabalin in generalized anxiety disorder patients with partial response to SSRI or SNRI treatment*. Int Clin Psychopharmacol* 2012; 27(3): 142-50. doi: 10.1097/YIC.0b013e328350b133.

**Social anxiety disorder/Social phobia**

**Feltner 2011**

- Feltner DE, Liu-Dumaw M, Schweizer E, Bielski R. Efficacy of pregabalin in generalized social anxiety disorder: results of a double-blind, placebo-controlled, fixed-dose study. *Int Clin Psychopharmacol* 2011; 26(4): 213-20. doi: 10.1097/YIC.0b013e32834519bd.

**Greist 2011**

- Greist JH, Liu-Dumaw M, Schweizer E, Feltner D. Efficacy of pregabalin in preventing relapse in patients with generalized social anxiety disorder: results of a double-blind, placebo-controlled 26-week study. *Int Clin Psychopharmacol* 2011; 26(5): 243-51. doi: 10.1097/YIC.0b013e3283491fd5.

**Pande 1999**

- Pande AC, Davidson JR, Jefferson JW, Janney CA, Katzelnick DJ, Weisler RH et al. Treatment of social phobia with gabapentin: a placebo-controlled study. *J Clin Psychopharmacol* 1999; 19(4): 341-8. doi: 10.1097/00004714-199908000-00010.

**Pande 2004**

- Pande AC, Feltner DE, Jefferson JW, Davidson JR, Pollack M, Stein MB et al. Efficacy of the novel anxiolytic pregabalin in social anxiety disorder: a placebo-controlled, multicenter study. *J Clin Psychopharmacol* 2004; 24(2): 141-9. doi: 10.1097/01.jcp.0000117423.05703.e7.

**Panic disorder**

**Pande 2000b**

- Pande AC, Pollack MH, Crockatt J, Greiner M, Chouinard G, Lydiard RB et al. Placebo-controlled study of gabapentin treatment of panic disorder. *J Clin Psychopharmacol* 2000; 20(4): 467-71. doi: 10.1097/00004714-200008000-00011.

**PTSD**

**Baniasadi 2014**

- Baniasadi M, Hosseini G, Fayyazi Bordbar MR, Rezaei Ardani A, Mostafavi Toroghi H. Effect of pregabalin augmentation in treatment of patients with combat-related chronic posttraumatic stress disorder: a randomized controlled trial. J *Psychiatr Pract* 2014; 20(6): 419-27. doi: 10.1097/01.pra.0000456590.12998.41.

**OCD**

**Mowla 2020**

- Mowla A, Ghaedsharaf M. Pregabalin augmentation for resistant obsessive-compulsive disorder: a double-blind placebo-controlled clinical trial. *CNS Spectr* 2020; 25(4): 552-556. doi: 10.1017/S1092852919001500.

**Insomnia**

**Bollu 2010**

- Bollu V, Bushmakin AG, Cappelleri JC, Chen C-C, Feltner D, Wittchen HU. Pregabalin reduces sleep disturbance in patients with generalized anxiety disorder via both direct and indirect mechanisms. *Eur J Psychiat* 2010; 24(1): 18-27. http://scielo.isciii.es/scielo.php?script=sci_arttext&pid=S0213-61632010000100003&lng=es&tlng=en.

**Brower 2008**

- Brower KJ, Myra Kim H, Strobbe S, Karam-Hage MA, Consens F, Zucker RA. A randomized double-blind pilot trial of gabapentin versus placebo to treat alcohol dependence and comorbid insomnia. *Alcohol Clin Exp Res* 2008; 32(8): 1429-38. doi: 10.1111/j.1530-0277.2008.00706.x.

**Furey 2014 (NCT00163046)**

- Furey SA, Hull SG, Leibowitz MT, Jayawardena S, Roth T. A randomized, double-blind, placebo-controlled, multicenter, 28-day, polysomnographic study of gabapentin in transient insomnia induced by sleep phase advance. *J Clin Sleep Med* 2014; 10(10): 1101-9. doi: 10.5664/jcsm.4110.

**Malcolm 2007**

- Malcolm R, Myrick LH, Veatch LM, Boyle E, Randall PK. Self-reported sleep, sleepiness, and repeated alcohol withdrawals: a randomized, double blind, controlled comparison of lorazepam vs gabapentin. *J Clin Sleep Med* 2007; 3(1):24-32.

**Mason 2014**

- Mason BJ, Quello S, Goodell V, Shadan F, Kyle M, Begovic A. Gabapentin treatment for alcohol dependence: a randomized clinical trial. *JAMA Intern Med* 2014; 174(1): 70-7. doi: 10.1001/jamainternmed.2013.11950.

**Mowla 2015**

- Mowla A, Ahmadzadeh L, Razeghian Jahromi L, Dastgheib SA. Comparing Gabapentin with Clonazepam for Residual Sleeping Problems following Antidepressant Therapy in Patients with Major Depressive Disorder: A Randomized Clinical Trial. *Clin Drug Investig* 2015; 35(8): 513-7. doi: 10.1007/s40261-015-0304-8.

**NCT01014533**

- NCT01014533. Pharmacotherapy and mechanisms of sleep disturbance in alcohol dependence (MA). <https://clinicaltrials.gov/ct2/show/results/NCT01014533> (first received 17 November 2009).

**Rosenberg 2014 (NCT00674752)**

- Rosenberg RP, Hull SG, Lankford DA, Mayleben DW, Seiden DJ, Furey SA et al. A randomized, double-blind, single-dose, placebo-controlled, multicenter, polysomnographic study of gabapentin in transient insomnia induced by sleep phase advance. *J Clin Sleep Med* 2014; 10(10): 1093-100. doi: 10.5664/jcsm.4108.

**Yurcheshen 2009**

- Yurcheshen ME, Guttuso T Jr, McDermott M, Holloway RG, Perlis M. Effects of gabapentin on sleep in menopausal women with hot flashes as measured by a Pittsburgh Sleep Quality Index factor scoring model. *J Womens Health (Larchmt)* 2009; 18(9): 1355-60. doi: 10.1089/jwh.2008.1257.

## Open label studies (k = 15)

**Bipolar disorder**

**Altshuler 1999**

- Altshuler LL, Keck PE, McElroy SL, Suppes T, Brown ES, Denicoff K et al. Gabapentin in the acute treatment of refractory bipolar disorder. *Bipolar Disord* 1999; 1: 61-65. doi:10.1034/j.1399-5618.1999.10113.x.

**Astaneh 2012**

- Astaneh AN, Rezaei O. Adjunctive treatment with gabapentin in bipolar patients during acute mania. *Int J Psychiatry Med* 2012; 43(3): 261-271. https://doi.org/10.2190/PM.43.3.e.

**Erfurth 1998**

- Erfurth A, Kammerer C, Grunze H, Normann C, Walden J. An open label study of gabapentin in the treatment of acute mania. *J Psychiatr Res* 1998; 32(5): 261-264. https://doi.org/10.1016/S0022-3956(98)00010-7.

**Knoll 1998**

- Knoll J, Stegman K, Suppes T. Clinical experience using gabapentin adjunctively in patients with a history of mania or hypomania. *J Affect Disord* 1998; 49(3): 229-233. https://doi.org/10.1016/S0165-0327(98)00027-5.

**Mauri 2001**

- Mauri MC, Laini V, Scalvini ME, Omboni A, Ferrari VMS, Clemente A, et al. Gabapentin and the prophylaxis of bipolar disorders in patients intolerant to lithium. *Clin Drug Investig* 2001; 21(3): 169-174. https://doi.org/10.2165/00044011-200121030-00002.

**McElroy 1997**

- McElroy SL, Soutullo CA, Keck PE, Kmetz GF. A pilot trial of adjunctive gabapentin in the treatment of bipolar disorder. *Ann Clin Psychiatry* 1997; 9: 99-103. https://doi.org/10.1023/A:1026257303275.

**Perugi 1999**

- Perugi G, Toni C, Ruffolo G, Sartini S, Simonini E, Akiskal H. Clinical experience using adjunctive gabapentin in treatment-resistant bipolar mixed states. *Pharmacopsychiatry* 1999; 32(4): 136-141. https://doi.org/10.1055/s-2007-979219.

**Schaffer 2012**

- Schaffer LC, Schaffer CB, Miller AR, Manley JL, Piekut JA, Nordahl TE. An open trial of pregabalin as an acute and maintenance adjunctive treatment for outpatients with treatment resistant bipolar disorder. *J Affect Disord* 2013; 147: 407-410. https://doi.org/10.1016/j.jad.2012.09.005.

**Wang 2002**

- Wang PW, Santosa C, Schumacher M, Winsberg ME, Strong C, Ketter TA. Gabapentin augmentation therapy in bipolar depression. *Bipolar Disord* 2002; 4: 296–301. https://doi.org/10.1034/j.1399-5618.2002.01211.x.

**Young 1997**

- Young LT, Robb JC, Patelis-Siotis I, MacDonald C, Joffe RT. Acute treatment of bipolar depression with gabapentin. Biol Psychiatry 1997; 42(9): 851-853. https://doi.org/10.1016/S0006-3223(97)00305-3.

**Young 1999**

- Young LT, Robb JC, Hasey GM, MacQueen GM, Siotis IP, Marriott M, et al. Gabapentin as an adjunctive treatment in bipolar disorder. *J Affect Disord* 1999; 55(1): 73-77. https://doi.org/10.1016/S0165-0327(98)00192-X.

**Generalised anxiety disorder (GAD)**

**Cvjetkovic-Bosnjak 2015**

- Cvjetkovic-Bosnjak M, Soldatovic-Stajic B, Babovic SS, Boskovic K, Jovicevic M. Pregabalin versus sertraline in generalized anxiety disorder. An open label study. *Eur Rev Med Pharmacol Sci* 2015; 19(11): 2120–2124.

**Generalised anxiety disorder, social anxiety disorder and panic disorder**

**Montgomery 2013**

- Montgomery S, Emir B, Haswell H, Prieto R. Long-term treatment of anxiety disorders with pregabalin: A 1 year open-label study of safety and tolerability. *Curr Med Res Opin* 2013; 29(10): 1223-1230. https://doi.org/10.1185/03007995.2013.820694.

**OCD**

**Oulis 2011**

- Oulis P, Mourikis I, Konstantakopoulos G. Pregabalin augmentation in treatment-resistant obsessive-compulsive disorder. *Int Clin Psychopharmacol* 2011; 26(4): 221-224. https://doi.org/10.1097/YIC.0b013e3283466657.

**Insomnia**

**NCT02040532**

- NCT02040532. Gabapentin for Insomnia Symptoms and Nighttime Vasomotor Symptoms (VMS) in Peri- and Postmenopausal Women. <https://clinicaltrials.gov/ct2/show/NCT02040532> (first received 20 January 2014).

# Table 1. Characteristics of all diagnostic groups

**Total DB-RCT studies, k = 55**

**Total participants randomized, n = 9012**

|  | **Total num.**  **of studies** |  |  | **Num. of randomised patients** | **Range of publication dates** | **Range of duration**  **of double-blind intervention** | **Primary outcome measures** | **Mean age, years** | **Mean % female** | **% DSM / % ICD** |
| --- | --- | --- | --- | --- | --- | --- | --- | --- | --- | --- |
|  |  | ***Num. >1 type of***  ***active investigational drug*** | ***Num. placebo controlled*** |  |  |  |  |  |  |  |
| Bipolar disorder (BD) | 4 | 3 | 2 | 231 | 2000 - 2006 | 6 – 52 weeks | MMPI-2 (Mokhber 2008)  YMRS, HAM-D (Pande 2000)  CGI-BP (Frye 2000)  CGI-BP-M (Vieta 2006) | 37.5 | 64.1% | 100% DSM-IV |
| Insomnia (all) | 9 | 7 | 1 | 1460 | 2007 - 2015 | Single dose –  12 weeks | ESS, PSQI, ISS, PSG-WASO, sleep diaries | 42.0 | 58.9% | 66.7% DSM-IV / 0% ICD |
| Insomnia  (gabapentin) | 8 | 6 | 0 | 1086 | 2007 - 2015 | Single dose –  12 weeks |  | 42.5 | 58.2 | 62.5% DSM-IV / 0% ICD |
| Insomnia  (pregabalin) | 1 | 1 | 1 | 374 | 2010 | 8 weeks |  | 40.8 | 60.7% | 100% DSM-IV-TR, GAD and related sleep disturbance |
| Anxiety studies (all) | 42 | 40 | 12 | 7321 | 1999 - 2020 | Single dose –  26 weeks |  | 43.0 | 60.4% | 100% DSM-IV or DSM-V, for all studies (excluding pre-op studies) |
| Generalized anxiety disorder  (pregabalin) | 11 | 10 | 5 | 3825 | 2003 - 2014 | 4 – 24 weeks | HAM-A | 43.1 | 62.7% | 100% DSM-IV |
| Pre-operative anxiety  (gabapentin) | 13 | 12 | 5 | 1062 | 2004 - 2015 | * | VAS 0-100, VAS 0-10,  NRS 0-10, STAI-S | 48.9 | 70.3% | * |
| Pre-operative anxiety  (pregabalin) | 10 | 10 | 1 | 1463 | 2009 - 2019 | * | VAS 0-100, VAS 0-10,  NRS 0-10, VRS 0-10 | 42.5 | 55.6% | * |
| Pre-operative anxiety  (gabapentin and pregabalin) | 1 | 1 | 1 | 90 | 2012 | * | VAS 0-10 | 44.7 | 100% | * |
| Social anxiety disorder (all) | 4 | 4 | 0 | 685 | 1999 - 2011 | 10 – 26 weeks | LSAS | 36.0 | 40.9% | 100% DSM-IV |
| Posttraumatic Stress Disorder (pregabalin) | 1 | 1 | 0 | 37 | 2014 | 6 weeks | PCL-M | 48.2 | 0 | 100% DSM-IV-TR |
| Panic disorder (gabapentin) | 1 | 1 | 0 | 103 | 2000 | 8 weeks | PAS | 35.0 | 63.4% | 100% DSM-IV |
| Obsessive Compulsive Disorder  (pregabalin) | 1 | 1 | 0 | 56 | 2020 | 12 weeks | Y-BOCS | 32.4 | 66.2% | 100% DSM-V |

**CGI-BP/CGI-BP-M,** Clinical Global Impression modified for use in Bipolar Disorder

**ESS,** Epworth Sleepiness Scale

**HAM-A,** Hamilton Anxiety Rating Scale

**HAM-D,** Hamilton Depressing Rating Scale

**ISS,** Insomnia Severity Scale

**LSAS,** Liebowitz Social Anxiety Scale

**MMPI-2,** Minnesota Multiphasic Personality Inventory-2

**NRS,** Numeric rating scale

**PAS,** Panic and Agoraphobia scale

**PCL-M,** PTSD Checklist-Military version

**PSG-WASO,** Polysomnography-derived Wake after sleep onset

**PSQI,** Pittsburgh Sleep Quality Index

**STAI-S,** State-Trait Anxiety Inventory (State anxiety scale)

**VAS,** Visual analogue scale

**VRS,** Verbal rating scale

**Y-BOCS,** Yale-Brown Obsessive Compulsive Scale

**YMRS,** Young Mania Rating Scale

# Table 2. Characteristics of included double-blind randomised controlled studies

| **Study name** | **No. of arms** | **Drug** | **No. patients randomised** | **Mean age (SD)** | **% Female** | **Dose**  **(min-max)** | | **Dosing Schedule** | **Mean dose delivered** | | **Baseline severity scale, threshold** | **Diagnosis/Procedure** | | **Study type** | | **Patient status** | | **Length of intervention** | | | | **Rescue medication**  ******* | | | | | **Industry**  **Sponsored** | | | | | | | |  |  |  |  |  |
| --- | --- | --- | --- | --- | --- | --- | --- | --- | --- | --- | --- | --- | --- | --- | --- | --- | --- | --- | --- | --- | --- | --- | --- | --- | --- | --- | --- | --- | --- | --- | --- | --- | --- | --- | --- | --- | --- | --- | --- |
|  |  | |  |  | | | **Bipolar Disorder** | | |  |  |  |  | | | | | | | | | | | | | | | | |  | | | | | | | | |  |
|  |  | |  |  | | | ***Gabapentin*** | | |  |  |  |  | | | | | | | | | | | | | | | | |  | | | | | | | | |  |
| Frye (2000)  (NCT00001482) | 3 | Gabapentin | 9 | * | * | 900-4800 (mg/day) | | Flexible | * | | * | DSM-IV, Refractory bipolar and unipolar affective illness (including BP I and BP II; rapid cycling and non-rapid cycling; and unipolar patients) | | DB Randomized  Cross-over  Monotherapy | | Inpatients | | 6 weeks | | | | Unclear | | | | | No | | | | | | | |  |  |  |  |  |
|  |  | Lamotrigine | 10 | * | * | 25-500 (mg/day) | | Flexible | * | | * |  |  |  |  |  |  |  |  |  |  |  | | | | |  | | | | | | | |  |  |  |  |  |
|  |  | Placebo | 11 | * | * | 0-0 | | Flexible | 0 | | * |  |  |  |  |  |  |  |  |  |  |  | | | | |  | | | | | | | |  |  |  |  |  |
| Mokhber (2008) | 3 | Gabapentin | 20 | 28.9 (10.6) | 50 | 900 (mg/day) | | Fixed | * | | * | Bipolar DSM-IV  Dysphoric mania. History of BP I. | | DB Randomized  Parallel  Monotherapy  Placebo run-in | | Outpatients | | 8 weeks | | | | Yes | | | | | Unclear | | | | | | | |  |  |  |  |  |
|  |  | Carbamazepine | 19 | 28.9 (9.6) | 62 | 600 (mg/day) | | Fixed | * | | * |  |  |  |  |  |  |  |  |  |  |  | | | | |  | | | | | | | |  |  |  |  |  |
|  |  | Lamotrigine | 20 | 27.8 (7.7) | 55 | 100 (mg/day) | | Fixed | * | | * |  |  |  |  |  |  |  |  |  |  |  | | | | |  | | | | | | | |  |  |  |  |  |
| Pande (2000)  (945-209) | 2 | Gabapentin  (+ Lithium, valproate, or combination) | 59 | 40.7 (9.4) | 50 | 600-3600 (mg/day) | | Flexible | * | | YMRS, >/=12 | DSM-IV  BP I, with manic/hypomanic or mixed symptoms | | DB Randomized  Parallel  Adjunctive | | Outpatients | | 10 weeks | | | | Unclear | | | | | Yes | | | | | | | |  |  |  |  |  |
|  |  | Placebo  (+ Lithium, valproate, or combination) | 58 | 38.2 (10.5) | 46 | 0-0 | | Flexible | 0 | | YMRS, >/=12 |  |  |  |  |  |  |  |  |  |  |  | | | | |  | | | | | | | |  |  |  |  |  |
| Vieta (2006)  (945-291) | 2 | Gabapentin + TAU | 13 | 46.2 (14.3) | 76.9 | 1200-2400 (mg/day) | | Flexible | * | | HAM-D, <8 | DSM-IV  Bipolar Depression | | DB Randomized  Parallel  Adjunctive | | Outpatients | | 52 weeks | | | | Unclear | | | | | Yes | | | | | | | |  |  |  |  |  |
|  |  | Placebo + TAU | 12 | 47.6 (15.8) | 66.7 | 0-0 | | Flexible | 0 | | HAM-D, <8 |  | |  |  |  |  |  |  | | | | | |  | | | | | |  | | | | | | | | |
|  |  | |  |  | | | **Insomnia/Sleep Disturbance** | | |  |  |  |  | | | | | | | | | | | | | | | | |  | | | | | | | | |  |
|  |  | |  |  | | | ***Gabapentin*** | | |  |  |  |  | | | | | | | | | | | | | | | | |  | | | | | | | | |  |
| Brower (2008) | 2 | Gabapentin | 10 | 46 (*) | 40 | 1500 (mg/day) | | Fixed | * | |  | DSM-IV  Alcohol Dependence and comorbid Insomnia | | DB Randomized  Parallel  Monotherapy  Placebo run-in | | Outpatients | | 6 weeks | | | | Unclear | | | | | Yes | | | | | | | |  |  |  |  |  |
|  |  | Placebo | 11 | 44 (*) | 54.5 | 0-0 | | Fixed | 0 | |  |  |  |  |  |  | |  |  |  |  |  | | | | |  | | | | | | | |  |  |  |  |  |
| Furey (2014) | 2 | Gabapentin | 128 | 41.4 (16.7) | 56.3 | 250 (mg/day) | | Fixed | 250 | | ESS, <10 | Healthy Volunteers with occasional disturbed sleep | | DB Randomized  Parallel  Monotherapy | | Outpatients | | 4 weeks | | | | Unclear | | | | | Yes | | | | | | | |  |  |  |  |  |
|  |  | Placebo | 128 | 41.5 (16.6) | 52.3 | 0-0 | | Fixed | 0 | | ESS, <10 |  | |  | |  | |  | | | |  | | | | |  | | | | | | | |  |  |  |  |  |
| Malcolm (2007) | 2 | Gabapentin | 101^a^ | * | * | 600-1200 (mg/day) | | Fixed | * | | CIWA-Ar,  >/=10 | DSM-IV  Alcohol Dependence and Alcohol Withdrawal Syndrome with Sleep Disturbance | | DB Randomized  Parallel  Monotherapy | | Outpatients | | 4 days | | | | Unclear | | | | | No | | | | | | | |  |  |  |  |  |
|  |  | Lorazepam | 101^a^ | * | * | 6 (mg/day) | | Fixed | * | | CIWA-Ar,  >/=10 |  |  |  |  |  | |  |  |  |  |  | | | | |  | | | | | | | |  |  |  |  |  |
| Mason (2014) (NCT00391716) | 3 | Gabapentin (low dose) | 54 | 41.9 (10.1) | 38.9 | 900 (mg/day) | | Fixed | * | | * | DSM-IV  Alcohol Dependence and related sleep disturbance | | DB Randomized  Parallel  Monotherapy | | Outpatients | | 12 weeks | | | | Unclear | | | | | Yes | | | | | | | |  |  |  |  |  |
|  |  | Gabapentin (high dose) | 47 | 45.2 (11.3) | 34 | 1800 (mg/day) | | Fixed | * | | * |  |  |  |  |  | |  |  |  |  |  | | | | |  | | | | | | | |  |  |  |  |  |
|  |  | Placebo | 49 | 46.8 (11.3) | 57.1 | 0-0 | | Fixed | * | | * |  |  |  |  |  | |  |  |  |  |  | | | | |  | | | | | | | |  |  |  |  |  |
| Mowla (2015) | 2 | Gabapentin + TAU | 31 | * | * | 100-600 (mg/day) | | Flexible | 400 | | PSQI,  >5,  ISI >8 | DSM-IV  Major Depressive Disorder with sleep problems | | DB Randomized  Parallel  Monotherapy | | Inpatients | | 4 weeks | | | | Unclear | | | | | No | | | | | | | |  |  |  |  |  |
|  |  | Clonazepam + TAU | 32 | * | * | 0.5-2 (mg/day) | | Flexible | 1 | | PSQI,  >5  ISI >8 |  | |  |  |  | |  |  |  |  |  | | | | |  | | | | | | | |  |  |  |  |  |
| NCT01014533  (HUM00010947) (1R01AA016117-01A1) | 2 | Gabapentin | 30 | 37 (8.9) | 23.3 | 600-1200 (mg/day) | | Fixed | * | | CIWA-Ar,  <8 | DSM-IV  Alcohol Dependence and related sleep disturbance | | DB Randomised  Parallel  Monotherapy | | Outpatients | | 1 week | | | | | Unclear | | | | | | Yes | | | | | | | | |  |  |
|  |  | Placebo | 29 | 34.9 (12.5) | 20.7 | 0-0 | | Fixed | * | | CIWA-Ar,  <8 |  |  |  |  |  |  | |  | | | | | |  | | | | | |  | | | | | | | | |
| Rosenberg (2014) (NCT00674752) | 3 | Gabapentin (low dose) | 125 | 42.2 (17.0) | 62.4 | 250 mg | | Fixed | 250 | | * | Healthy Volunteers with occasional disturbed sleep | | DB Randomised  Parallel  Monotherapy | | Outpatients | | Single dose | | | | | Unclear | | | | | | Yes | | | | | | | | |  |  |
|  |  | Gabapentin (high dose) | 125 | 40.7 (17.1) | 68.8 | 500 mg | | Fixed | 500 | | * |  |  |  |  |  | |  |  |  |  |  |  | | | | | |  | | | | | | | | |  |  |
|  |  | Placebo | 127 | 42.0 (15.6) | 68.5 | 0-0 | | Fixed | 0 | | * |  |  |  |  |  | |  |  |  |  |  |  | | | | | |  | | | | | | | | |  |  |
| Yurcheshen (2009) | 2 | Gabapentin | 30 | 52.7 (3.6) | 100 | 900 (mg/day) | | Fixed | 900 | | * | Post-menopausal women experiencing at least seven hot flushes per day | | DB Randomized  Parallel  Monotherapy | | Outpatients | | 12 weeks | | | | | No | | | | | | No | | | | | | | | |  |  |
|  |  | Placebo | 29 | 53 (3.1) | 100 | 0-0 | | Fixed | 0 | | * |  |  |  |  |  | |  |  |  |  |  |  | | | | | |  | | | | | | | | |  |  |
|  |  | |  |  | | | ***Pregabalin*** | | |  |  |  |  | | | | | | | | | | | | | | | | |  | | | | | | | | |  |
| Bollu (2010) | 3 | Pregabalin | 121 | 39.5 (11.9) | 63.6 | 300-600 (mg/day) | | Flexible | * | | * | DSM-IV-TR Generalized Anxiety Disorder and related sleep disturbance | | DB Randomized  Parallel  Monotherapy  Placebo run-in | | Outpatients | | 8 weeks | | | | | No | | | | | | Yes | | | | | | | | |  |  |
|  |  | Venlafaxine | 125 | 42.6 (11.8) | 57.6 | 75-225 (mg/day) | | Flexible | * | | * |  |  |  |  |  |  | |  | | | | | |  | | | | | |  | | | | | | | | |
|  |  | Placebo | 128 | 40.2 (12.1) | 60.9 | 0-0 | | Flexible | * | | * |  |  |  |  |  |  | |  |  |  |  |  |  |  | | | | | |  | | | | | | | | |
|  |  | |  |  | | | **Generalized Anxiety Disorder** | | |  |  |  |  | | | | | | | | | | | | | | | | |  | | | | | | | | |  |
|  |  | |  |  | | | ***Pregabalin*** | | |  |  |  |  | | | | | | | | | | | | | | | | |  | | | | | | | | |  |
| Feltner (2003) | 4 | Pregabalin (low dose) | 70 | 37.9 (10.9) | 51.4 | 150 (mg/day) | | Fixed | 150 | | HAM-A >/= 20 | DSM-IV  Generalized Anxiety Disorder | | | DB Randomized  Parallel  Monotherapy | Outpatient | | 4 weeks | | | Unclear | | | | | | | Yes | | | | |  | | |  |  |  |  |
|  |  | Pregabalin (high dose) | 66 | 36.3 (10.9) | 50.0 | 600 (mg/day) | | Fixed | 600 | | HAM-A >/= 20 |  |  |  |  |  |  |  | | |  |  |  |  |  |  |  |  | | | | |  | | |  |  |  |  |
|  |  | Lorazepam | 68 | 39.2 (11.7) | 58.8 | 6 (mg/day) | | Fixed | 6 | | HAM-A >/= 20 |  |  |  |  |  |  |  |  | | | | |  | | | | | | |  | | | | | |  | | |
|  |  | Placebo | 67 | 37.8 (10.8) | 50.7 | 0-0 | | Fixed | 0 | | HAM-A >/= 20 |  |  |  |  |  |  |  |  | | | | |  |  |  |  |  |  |  |  | | | | | |  | | |
| Feltner (2008) | 2 | Pregabalin | 168 | 38.7 (*) | 55.4 | 450 (mg/day) | | Fixed | 450 | | HAM-A </= 11 | DSM-IV  Generalized Anxiety Disorder | | | DB Randomized  Parallel  Monotherapy | Outpatient | | 24 weeks | | Unclear | | | | | | Yes | | | | | |  | |  |  |  |  |  |  |
|  |  | Placebo | 170 | 38.8 (*) | 58.8 | 0-0 | | Fixed | 0 | | HAM-A </= 11 |  |  |  |  |  |  |  | |  |  |  |  |  |  |  | | | | | |  | |  |  |  |  |  |  |
| Hadley (2012)  (NCT00368745)  (2006-001347-66) | 2 | Pregabalin | 56 | 40.1 (10.6) | 75 | 150-600 (mg/day) | | Flexible | * | | * | DSM-IV  Generalized Anxiety Disorder, on benzodiazepine (BDZ) | | | DB Randomized  Parallel  Monotherapy | Outpatient | | 12 weeks | | | Yes | | | | | | | Yes | | | | |  | | |  |  |  |  |
|  |  | Placebo | 50 | 43.5 (11.3) | 68 | 0-0 | | Flexible | 0 | | * |  |  |  | BDZ taper for up to 6 weeks from baseline |  |  |  | | |  |  |  |  |  |  |  |  |  |  |  |  |  | | |  |  |  |  |
| Kasper (2009)  (NCT00151450)  (EUCTR2004-001500-13-IE) | 3 | Pregabalin | 121 | 39.5 (11.9) | 64 | 300-600 (mg/day) | | Fixed | * | | HAM-A >/= 20 | DSM-IV  Generalized Anxiety Disorder | | | DB Randomized  Parallel  Monotherapy | Outpatient | | 8 weeks | | | Unclear | | | | | | | Yes | | | | |  | | |  |  |  |  |
|  |  | Venlafaxine | 125 | 42.6 (11.8) | 58 | 75-225 (mg/day) | | Fixed | * | | HAM-A >/= 20 |  |  |  |  |  |  |  |  | | | | |  | | | | | | |  | | | | | |  | | |
|  |  | Placebo | 128 | 40.2 (12.1) | 61 | 0-0 | | 0 |  | | HAM-A >/= 20 |  |  |  |  |  |  |  |  | | | | |  |  |  |  |  |  |  |  | | | | | |  | | |
| Kasper (2014)  (NCT00624780)  (EUCTR2007-004768-32-ES) | 3 | Pregabalin (low dose) | 206 | 40.5 (12.3) | 64.6 | 150-300 (mg/day) | | Flexible | * | | HAM-A >/= 14 | DSM-IV  Generalized Anxiety Disorder | | | DB Randomized  Parallel  Monotherapy | * | | 12 weeks | | No | | | | | | Yes | | | | | |  | |  |  |  |  |  |  |
|  |  | Pregabalin (high dose) | 206 | 42.4 (11.5) | 57.8 | 450-600 (mg/day) | | Flexible | * | | HAM-A >/= 14 |  |  |  |  |  |  |  |  | | | | |  | | | | | | |  | | | | | |  | | |
|  |  | Lorazepam | 203 | 42.6 (11.2) | 60.1 | 3-4 (mg/day) | | Flexible | * | | HAM-A >/= 14 |  |  |  |  |  |  |  |  | | | | |  |  |  |  |  |  |  |  | | | | | |  | | |
| Montgomery (2006) | 4 | Pregabalin (low dose) | 97 | 45.0 (12.0) | 59 | 400 (mg/day) | | Fixed | 376 | | HAM-A >/= 20 | DSM-IV  Generalized Anxiety Disorder | | | DB Randomized  Parallel  Monotherapy | Outpatient | | 6 weeks | | Unclear | | | | | | Yes | | | | | |  | |  |  |  |  |  |  |
|  |  | Pregabalin (high dose) | 110 | 42.0 (12.0) | 65 | 600 (mg/day) | | Fixed | 557 | | HAM-A >/= 20 |  |  |  |  |  |  |  |  | | | | |  | | | | | | |  | | | | | |  | | |
|  |  | Venlafaxine | 113 | 46.0  (12.0) | 65 | 75 (mg/day) | | Fixed | 75 | | HAM-A >/= 20 |  |  |  |  |  |  |  |  | | | | |  |  |  |  |  |  |  |  | | | | | |  | | |
|  |  | Placebo | 101 | 43.0 (12.0) | 58 | 0-0 | | Fixed | 0 | | HAM-A >/= 20 |  |  |  |  |  |  |  |  | | | | |  |  |  |  |  |  |  |  | | | | | |  | | |
| Montgomery (2008)  (EUCTR2004-000955-40-LV) | 2 | Pregabalin | 177 | 72.4 (5.6) | 79 | 150-600 (mg/day) | | Flexible | * | | HAM-A >/= 20,  MMSE >/= 24 | DSM-IV  Generalized Anxiety Disorder | | | DB Randomized  Parallel  Monotherapy | Outpatient | | 8 weeks | | Unclear | | | | | | Yes | | | | | |  | |  |  |  |  |  |  |
|  |  | Placebo | 96 | 72.2 (6.4) | 75 | 0-0 | | Flexible | 0 | | HAM-A >/= 20,  MMSE >/= 24 |  |  |  |  |  |  |  |  | | | | |  | | | | | | |  | | | | | |  | | |
| Pande (2003) | 4 | Pregabalin (low dose) | 69 | 37.9 (11.8) | 49.3 | 150 (mg/day) | | Fixed | 150 | | HAM-A >/= 20 | DSM-IV  Generalized Anxiety Disorder | | | DB Randomized  Parallel  Monotherapy | Outpatient | | 4 weeks | | Unclear | | | | | | Yes | | | | | |  | | |  |  |  |  |  |
|  |  | Pregabalin (high dose) | 70 | 35.5 (11.2) | 57.1 | 600 (mg/day) | | Fixed | 600 | | HAM-A >/= 20 |  |  |  |  |  |  |  |  | | | | |  | | | | | | |  | | | | | |  | | |
|  |  | Lorazepam | 68 | 33.9 (9.7) | 63.2 | 6 (mg/day) | | Fixed | 6 | | HAM-A >/= 20 |  |  |  |  |  |  |  |  | | | | |  |  |  |  |  |  |  |  | | | | | |  | | |
|  |  | Placebo | 69 | 35.7 (11.5) | 68.1 | 0-0 | | Fixed | 0 | | HAM-A >/= 20 |  |  |  |  |  |  |  |  | | | | |  |  |  |  |  |  |  |  | | | | | |  | | |
| Pohl (2005) | 4 | Pregabalin (low dose) | 78 | * | * | 200 (mg/day) | | Fixed | 200 | | HAM-A >/= 20 | DSM-IV  Generalized Anxiety Disorder | | | DB Randomized  Parallel  Monotherapy | Outpatient | | 6 weeks | | Unclear | | | | | | Yes | | | | | |  | | |  |  |  |  |  |
|  |  | Pregabalin (medium dose) | 89 | * | * | 400 (mg/day) | | Fixed | 385 | | HAM-A >/= 20 |  |  |  |  |  |  |  |  | | | | |  | | | | | | |  | | | | | |  | | |
|  |  | Pregabalin (high dose) | 88 | * | * | 450 (mg/day) | | Fixed | 440 | | HAM-A >/= 20 |  |  |  |  |  |  |  |  | | | | |  |  |  |  |  |  |  |  | | | | | |  | | |
|  |  | Placebo | 86 | * | * | 0-0 | | Fixed | 0 | | HAM-A >/= 20 |  |  |  |  |  |  |  |  | | | | |  |  |  |  |  |  |  |  | | | | | |  | | |
| Rickels (2005) | 5 | Pregabalin (low dose) | 91 | 38.0 (10.0) | 64 | 300 (mg/day) | | Fixed | 300 | | HAM-A >/= 20 | DSM-IV  Generalized Anxiety Disorder | | | DB Randomized  Parallel  Monotherapy  Placebo run-in | Outpatient | | 4 weeks | | Unclear | | | | | | Yes | | | | | |  | | |  |  |  |  |  |
|  |  | Pregabalin (medium dose) | 90 | 38.0 (12.0) | 59 | 450 (mg/day) | | Fixed | 450 | | HAM-A >/= 20 |  |  |  |  |  |  |  | |  |  |  |  |  |  |  | | | | | |  | | |  |  |  |  |  |
|  |  | Pregabalin (high dose) | 89 | 39.0 (12.0) | 67 | 600 (mg/day) | | Fixed | 600 | | HAM-A >/= 20 |  |  |  |  |  |  |  | |  |  |  |  |  |  |  | | | | | |  | | |  |  |  |  |  |
|  |  | Alprazolam | 93 | 40.0 (12.0) | 66 | 1.5 (mg/day) | | Fixed | 1.5 | | HAM-A >/= 20 |  |  |  |  |  |  |  | |  |  |  |  |  |  |  | | | | | |  | | |  |  |  |  |  |
|  |  | Placebo | 91 | 41.0 (12.0) | 63 | 0-0 | | Fixed | 0 | | HAM-A >/= 20 |  |  |  |  |  |  |  | |  |  |  |  |  |  |  | | | | | |  | | |  |  |  |  |  |
| Rickels (2012)  (NCT00413010)  (EUCTR2006-006339-31-FI) | 2 | Pregabalin  (+ SSRI/SNRI) | 180 | 43.7 (11.5) | 71.7 | 150-600 (mg/day) | | Flexible. Fixed in final 2 weeks | 431.125 | | HAM-A >/= 22 (for open-label phase), Responder rate <50% to enter DB phase | DSM-IV  Generalized Anxiety Disorder | | | DB Randomized  Parallel  Adjunct  Preceded by 8-week, open-label phase with SSRI/SNRI | Outpatients | | 8 weeks | | Unclear | | | | | | Yes | | | | | |  | | |  |  |  |  |  |
|  |  | Placebo  (+ SSRI/SNRI) | 176 | 43.5 (12.5) | 65.3 | 0-0 | | Flexible. Fixed in final 2 weeks | 0 | | HAM-A >/= 22 (for open-label phase), Responder rate <50% to enter DB phase |  |  |  |  |  |  |  |  |  |  |  |  |  |  |  | | | | | |  | | |  |  |  |  |  |
|  |  |  |  |  |  |  |  |  |  |  |  |  |  |  |  |  |  |  |  |  |  |  |  |  |  |  | | | | | |  | | |  |  |  |  |  |
|  |  | |  |  | | | **Pre-operative Anxiety** | | |  |  |  |  | | | | | | | | | | | | | | | | |  | | | | | | | | |  |
|  |  | |  |  | | | ***Gabapentin*** | | |  |  |  |  | | | | | | | | | | | | | | | | |  | | | | | | | | |  |
| Abdel-Halim (2009) | 4 | Gabapentin | 20 | 45.0 (14.0) | 100 | 800 mg | | Single dose | 800 mg | |  | Radical mastectomy | | | DB Randomized   Parallel | Pre-operative, elective,  GA | | Single dose | | Midazolam IV 2-4mg | | | | | | Unclear | | | | | |  | | |  |  |  |  |  |
|  |  | Dexamethasone IV | 20 | 48.0 (15.0) | 100 | 16 mg | | Single dose | 16 mg | |  |  |  |  |  |  |  |  |  |  |  |  |  |  |  |  | | | | | |  | | |  |  |  |  |  |
|  |  | Gabapentin + Dexamethasone IV | 20 | 46.0 (10.0) | 100 | 800 mg  16 mg | | Single dose | 800 mg  16 mg | |  |  |  |  |  |  |  |  |  | | | | |  | | | | | | |  | | | | | |  | | |
|  |  | No intervention | 20 | 46.0 (16.0) | 100 | 0-0 | | Single dose | 0 | |  |  |  |  |  |  |  |  |  | | | | |  | | | | | | |  | | | | | |  | | |
| Adam (2012) | 2 | Gabapentin | 32 | 36.0 (12.0) | 43 | 1200 mg | | Single dose | 1200 mg | |  | Orthopedic Surgery/Open Inguinal Hernia Repair | | | DB Randomized   Parallel | Pre-operative, elective,  GA | | Single dose | | No | | | | | | No | | | | | |  | | |  |  |  |  |  |
|  |  | Placebo | 32 | 39.0 (14.0) | 34 | 0-0 | | Single dose | 0 | |  |  |  |  |  |  |  |  |  |  |  |  |  |  |  |  | | | | | |  | | |  |  |  |  |  |
| Bakry (2012) | 2 | Gabapentin | 30 | 63.2 (4.8) | 27 | 1200 mg | | Single dose | 1200 mg | |  | Cataract Surgery | | | DB Randomized   Parallel | Pre-operative, elective,  LA | | Single dose | | No | | | | | | No | | | | | |  | | |  |  |  |  |  |
|  |  | Placebo | 30 | 61.3 (5.5) | 30 | 0-0 | | Single dose | 0 | |  |  |  |  |  |  |  |  |  |  |  |  |  |  |  |  | | | | | |  | | |  |  |  |  |  |
| Clarke (2010) | 2 | Gabapentin | 22 | Med (IQR)  59 (53-69) | 43.5 | 600 mg | | Single dose | 600 mg | |  | Total hip arthroplasty | | | DB Randomized   Parallel | Pre-operative, elective,  GA | | Single dose | | No | | | | | | No | | | | | |  | | |  |  |  |  |  |
|  |  | Placebo | 48 | 63.5 (55.5-68.5) | 45.8 | 0-0 | | Single dose | 0 | |  |  |  |  |  |  |  |  |  |  |  |  |  |  |  |  | | | | | |  | | |  |  |  |  |  |
| Clarke (2013) | 2 | Gabapentin | 25 | 41.6 (6.6) | 100 | 1200 mg | | Single dose | 1200 mg | |  | Non-Cardiac Surgery | | | DB Randomized   Parallel | Pre-operative,  GA | | Single dose | | No | | | | | | No | | | | | |  | | |  |  |  |  |  |
|  |  | Placebo | 25 | 41.8 (6.8) | 100 | 0-0 | | Single dose | 0 | |  |  |  |  |  |  |  |  |  |  |  |  |  |  |  |  | | | | | |  | | |  |  |  |  |  |
| Hoseini (2015)  (IRCT2014041217231N1) | 4 | Gabapentin | 22 | 40.5 (8.38) | * | 600 mg | | Single dose | 600 mg | |  | Laparoscopic Cholecystectomy | | | DB Randomized    Parallel | Pre-operative,  GA | | Single dose | | No | | | | | | Unclear | | | | | |  | | |  |  |  |  |  |
|  |  | Melatonin | 22 | 39.45 (11.4) | * | 6 mg | | Single dose | 6 mg | |  |  |  |  |  |  |  |  |  |  |  |  |  |  |  |  |  |  |  |  |  |  | | |  |  |  |  |  |
|  |  | Clonidine | 22 | 44.14 (8.41) | * | 0.2 mg | | Single dose | 0.2 mg | |  |  |  |  |  |  |  |  |  |  |  |  |  |  |  |  |  |  |  |  |  |  | | |  |  |  |  |  |
|  |  | Placebo | 22 | 38.14 (10.8) | * | 0-0 | | Single dose | 0 | |  |  |  |  |  |  |  |  |  |  |  |  |  |  |  |  |  |  |  |  |  |  | | |  |  |  |  |  |
| Joseph (2014)  (CTRI/2010/091/002830) | 3 | Gabapentin | 25 | 43.3 (8.6) | 100 | 600 mg | | Single dose | 600 mg | |  | Hysterectomy | | | DB Randomized   Parallel | Pre-operative, Elective,  GA | | Single dose | | Unclear | | | | | | No | | | | | |  | | |  |  |  |  |  |
|  |  | Alprazolam | 25 | 46.1 (9.3) | 100 | 0.5 mg | | Single dose | 0.5 mg | |  |  |  |  |  |  |  |  |  |  |  |  |  |  |  |  | | | | | |  | | |  |  |  |  |  |
|  |  | Placebo | 25 | 44.4 (5.6) | 100 | 0-0 | | Single dose | 0 | |  |  |  |  |  |  |  |  |  | | | | |  | | | | | | |  | | | | | |  | | |
| Khezri (2013)  (NCT01200641)  (ACTRN12610000727044) | 3 | Gabapentin | 40 | 75.6 (10.1) | 73.9 | 600 mg | | Single dose | 600 mg | |  | Cataract Surgery | | | DB Randomized   Parallel | Pre-operative,  Elective,  LA | | Single dose | | No | | | | | | No | | | | | |  | | |  |  |  |  |  |
|  |  | Melatonin | 40 | 73.5 (11.3) | 60 | 6 mg | | Single dose | 6 mg | |  |  |  |  |  |  |  |  |  |  |  |  |  |  |  |  | | | | | |  | | |  |  |  |  |  |
|  |  | Placebo | 40 | 72.9 (10.8) | 66.6 | 0-0 | | Single dose | 0 | |  |  |  |  |  |  |  |  |  |  |  |  |  |  |  |  | | | | | |  | | |  |  |  |  |  |
| Ménigaux (2005) | 2 | Gabapentin | 20 | 31.0 (8.0) | 30 | 1200 mg | | Single dose | 1200 mg | |  | Knee Arthroscopy | | | DB Randomized   Parallel | Pre-operative, Elective,  GA | | Single dose | | No | | | | | | No | | | | | |  | | |  |  |  |  |  |
|  |  | Placebo | 20 | 31.0 (8.0) | 35 | 0-0 | | Single dose | 0 | |  |  |  |  |  |  |  |  |  |  |  |  |  |  |  |  | | | | | |  | | |  |  |  |  |  |
| Pathak (2014) | 2 | Gabapentin | 40 | 41.5 (11.6) | 82.5 | 1200 mg | | Single dose | 1200 mg | |  | Open Cholecystectomy | | | DB Randomized   Parallel | Pre-operative,  Elective,  GA | | Single dose | | No | | | | | | Unclear | | | | | |  | | |  |  |  |  |  |
|  |  | Placebo | 40 | 37.4 (11.1) | 90 | 0-0 | | Single dose | 0 | |  |  |  |  |  |  |  |  |  |  |  |  |  |  |  |  | | | | | |  | | |  |  |  |  |  |
| Rorarius (2004) | 2 | Gabapentin | 38 | Med (IQR)  47 (44.8-50) | 100 | 1200 mg | | Single dose | 1200 mg | |  | Vaginal hysterectomy | | | DB Randomized   Parallel | Pre-operative,  Elective,  GA | | Single dose | | No | | | | | | No | | | | | |  | | |  |  |  |  |  |
|  |  | Oxazepam | 37 | 45 (42-50.5) | 100 | 15 mg | | Single dose | 15 mg | |  |  |  |  |  |  |  |  |  |  |  |  |  |  |  |  | | | | | |  | | |  |  |  |  |  |
| Sava (2009) | 2 | Gabapentin | 25 | * | * | 800 mg | | Single dose | 800 mg | |  | Rectocolic surgery | | | DB Randomized   Parallel | Pre-operative, Elective,  GA | | Single dose | | No | | | | | | Unclear | | | | | |  | | |  |  |  |  |  |
|  |  | Placebo | 25 | * | * | 0-0 | | Single dose | 0 | |  |  |  |  |  |  |  |  |  |  |  |  |  |  |  |  | | | | | |  | | |  |  |  |  |  |
| Tirault (2010) | 3 | Gabapentin | 70 | 46.0 (12.0) | 71 | 1200 mg | | Single dose | 1200 mg | |  | Various elective surgeries requiring GA | | | DB Randomized  Parallel | Pre-operative, Elective,  GA | | Single dose | | Unclear | | | | | | Unclear | | | | | |  | | |  |  |  |  |  |
|  |  | Hydroxyzine | 70 | 47.0 (12.0) | 62 | 75 mg | | Single dose | 75 mg | |  |  |  |  |  |  |  |  |  |  |  |  |  |  |  |  | | | | | |  | | |  |  |  |  |  |
|  |  | Placebo | 70 | 44.0 (13.0) | 58 | 0-0 | | Single dose | 0 | |  |  |  |  |  |  |  |  | |  |  |  |  |  |  |  | | | | | |  | | |  |  |  |  |  |
|  |  | |  |  | | | ***Pregabalin*** | | |  |  |  |  | | | | | | | | | | | | | | | | |  | | | | | | | | |  |
| Gonano (2011) | 2 | Pregabalin | 20 | 45.1 (10.2) | 40 | 300 mg | | Single dose | 300 mg | |  | Knee arthroscopic meniscectomy | | | DB Randomized  Parallel | Pre-operative, Elective,  GA | | Single dose | | No | | | | | | No | | | | | |  | | |  |  |  |  |  |
|  |  | Placebo | 20 | 41.8 (11.2) | 30 | 0-0 | | Single dose | 0 | |  |  |  |  |  |  |  |  |  |  |  |  |  |  |  |  | | | | | |  | | |  |  |  |  |  |
| Moreau-Bussiere (2013) (NCT01158859) | 2 | Pregabalin | 25 | Med (IQR) 36 (31-38) | 100 | 150 mg | | Single dose | 150 mg | |  | Laparoscopic Gynecologic Procedures | | | DB Randomized  Parallel | Pre-operative, Elective,  GA | | Single dose | | No | | | | | | No | | | | | |  | | |  |  |  |  |  |
|  |  | Placebo | 25 | 33 (29-36) | 100 | 0-0 | | Single dose | 0 | |  |  |  |  |  |  |  |  |  |  |  |  |  |  |  |  | | | | | |  | | |  |  |  |  |  |
| Nasr (2014) | 2 | Pregabalin | 20 | 31.0 (5.0) | 100 | 150 mg | | Single dose | 150 mg | |  | Laparoscopic Adhesiolysis | | | DB Randomized  Parallel | Pre-operative, Elective,  GA | | Single dose | | No | | | | | | No | | | | | |  | | |  |  |  |  |  |
|  |  | Melatonin | 20 | 32.5 (4.0) | 100 | 6 mg | | Single dose | 6 mg | |  |  |  |  |  |  |  |  |  |  |  |  |  |  |  |  | | | | | |  | | |  |  |  |  |  |
| NCT00468845 | 3 | Pregabalin | 162 | Range 25-70 | 100 | 150 mg | | Single dose | 150 mg | |  | Hysterectomy | | | DB Randomized  Parallel | Pre-operative,  GA | | Single dose | | Yes | | | | | | Yes | | | | | |  | | |  |  |  |  |  |
|  |  | Pregabalin | 170 | Range 25-71 | 100 | 300 mg | | Single dose | 300 mg | |  |  |  |  |  |  |  |  | |  |  |  |  |  |  |  | | | | | |  | | |  |  |  |  |  |
|  |  | Placebo | 169 | Range 25-72 | 100 | 0-0 | | Single dose | 0 | |  |  |  |  |  |  |  |  | |  |  |  |  |  |  |  | | | | | |  | | |  |  |  |  |  |
| NCT00551135 | 4 | Pregabalin (low dose) | 108 | * | 0 | 50 mg | | Multiple dosing | ** | |  | Inguinal Hernia Repair | | | DB Randomized  Parallel | Pre-operative, Multi center,  GA | | Multiple dosing** | | No | | | | | | Yes | | | | | |  | | |  |  |  |  |  |
|  |  | Pregabalin (medium dose) | 106 | * | 0 | 150 mg | | Multiple dosing | ** | |  |  |  |  |  |  |  |  |  |  |  |  |  |  |  |  | | | | | |  | | |  |  |  |  |  |
|  |  | Pregabalin (high dose) | 103 | * | 0 | 300 mg | | Multiple dosing | ** | |  |  |  |  |  |  |  |  |  |  |  |  |  |  |  |  | | | | | |  | | |  |  |  |  |  |
|  |  | Placebo | 108 | * | 0 | 0-0 | | Multiple dosing | 0 | |  |  |  |  |  |  |  |  |  |  |  |  |  |  |  |  | | | | | |  | | |  |  |  |  |  |
| Nutt (2009)  (EUCTR2005-003006-29-GB) | 3 | Pregabalin | 27 | 35.1 (12.2) | 70.4 | 150 mg | | Single dose | 150 mg | |  | Dental Procedure,  with Dental Anxiety Total Score >/= 12 at the screen visit and at baseline on day of procedure | | | DB Randomized  Parallel | Pre-operative, Multi center, Outpatients | | Single dose | | No | | | | | | Yes | | | | | |  | | |  |  |  |  |  |
|  |  | Alprazolam | 31 | 41.8 (12.4) | 51.6 | 0.5 mg | | Single dose | 0.5 mg | |  |  |  |  |  |  |  |  |  |  |  |  |  |  |  |  | | | | | |  | | |  |  |  |  |  |
|  |  | Placebo | 31 | 36.8 (12.7) | 64.5 | 0-0 | | Single dose | 0 | |  |  |  |  |  |  |  |  |  |  |  |  |  |  |  |  | | | | | |  | | |  |  |  |  |  |
| Shimony (2016)  (NCT01612832) | 2 | Pregabalin | 50 | 52.6 (15.5) | 48 | 150 mg | | Single dose | 150 mg | |  | Craniotomy | | | DB Randomized  Parallel | Pre-operative, Elective,  GA | | Single dose | | No | | | | | | No | | | | | |  | | |  |  |  |  |  |
|  |  | Placebo | 50 | 51.6 (16.8) | 45 | 0-0 | | Single dose | 0 | |  |  |  |  |  |  |  |  | |  |  |  |  |  |  |  | | | | | |  | | |  |  |  |  |  |
| Singh (2019) | 2 | Pregabalin | 30 | 40.9 (12.2) | 50 | 150 mg | | Single dose | 150 mg | |  | Laparoscopic Cholecystectomy | | | DB Randomized  Parallel | Pre-operative,  Elective,  GA | | Single dose | | No | | | | | | No | | | | | |  | | |  |  |  |  |  |
|  |  | Placebo | 30 | 36.3 (11.9) | 50 | 0-0 | | Single dose | 0 | |  |  |  |  |  |  |  |  |  |  | | | | | |  | | | | | |  | | |  |  |  |  |  |
| Spreng (2011)  (NCT00353704) | 2 | Pregabalin | 25 | 44.1 (10.8) | 50 | 150 mg | | Single dose | 150 mg | |  | Discectomy | | | DB Randomized  Parallel | Pre-operative, Elective,  GA | | Single dose | | Unclear | | | | | | No | | | | | |  | | |  |  |  |  |  |
|  |  | Placebo | 25 | 42.9 (7.6) | 46 | 0-0 | | Single dose | 0 | |  |  |  |  |  |  |  |  |  |  |  |  |  |  |  |  | | | | | |  | | |  |  |  |  |  |
| White (2009) | 4 | Pregabalin (low dose) | 27 | 43.0 (14.0) | 55.6 | 75 mg | | Single dose | 75 mg | |  | Elective ambulatory and short-stay (<24h) procedures, including ENT, General surgery, Plastics, Urologic | | | DB Randomized  Parallel | Pre-operative, Elective | | Single dose | | No | | | | | | Yes | | | | | |  | | |  |  |  |  |  |
|  |  | Pregabalin (medium dose) | 27 | 48.0 (16.0) | 55.6 | 150 mg | | Single dose | 150 mg | |  |  |  |  |  |  |  |  |  |  |  |  |  |  |  |  |  |  |  |  |  |  | | |  |  |  |  |  |
|  |  | Pregabalin (high dose) | 27 | 46.0 (13.0) | 29.6 | 300 mg | | Single dose | 300 mg | |  |  |  |  |  |  |  |  |  |  |  |  |  |  |  |  |  |  |  |  |  |  | | |  |  |  |  |  |
|  |  | Placebo | 27 | 48.0 (15.0) | 55.6 | 0-0 | | Single dose | 0 | |  |  |  |  |  |  |  |  |  |  |  |  |  |  |  |  |  |  |  |  |  |  | | |  |  |  |  |  |
|  |  | |  |  | | | ***Gabapentin & Pregabalin*** | | |  |  |  |  | | | | | | | | | | | | | | | | |  | | | | | | | | |  |
| Ghai (2012) | 3 | Gabapentin | 30 | 45.6 (5.9) | 100 | 900 mg | | Single dose | 900 mg | |  | Hysterectomy | | | DB Randomized   Parallel | Pre-operative,  Elective,  GA | | Single dose | | No | | | | | | No | | | | | |  | | |  |  |  |  |  |
|  |  | Pregabalin | 30 | 45.1 (6.5) | 100 | 300 mg | | Single dose | 300 mg | |  |  |  |  |  |  |  |  |  |  |  |  |  |  |  |  | | | | | |  | | |  |  |  |  |  |
|  |  | Placebo | 30 | 43.4 (5.7) | 100 | 0-0 | | Single dose | 0 | |  |  |  |  |  |  |  |  |  |  |  |  |  |  |  |  | | | | | |  | | |  |  |  |  |  |
|  |  | |  |  | | | **Posttraumatic Stress Disorder** | | |  |  |  |  | | | | | | | | | | | | | | | | |  | | | | | | | | |  |
|  |  | |  |  | | | ***Pregabalin*** | | |  |  |  |  | | | | | | | | | | | | | | | | |  | | | | | | | | |  |
| Baniasadi (2014) | 2 | Pregabalin | 18 | 47.7 (3.3) | 0 | 300 (mg/day) | | Fixed | * | | * | DSM-IV-TR  Chronic Posttraumatic Stress Disorder | | | DB Randomized  Parallel  Adjunct | Inpatients | | 6 weeks | | Unclear | | | | | | No | | | | | |  | | |  |  |  |  |  |
|  |  | Placebo | 19 | 48.6 (3.8) | 0 | 0-0 | | Fixed | 0 | | * |  |  |  |  |  |  |  |  |  |  |  |  |  |  |  | | | | | |  | | |  |  |  |  |  |
|  |  | |  |  | | | **Social Anxiety Disorder (Social Phobia)** | | |  |  |  |  | | | | | | | | | | | | | | | | |  | | | | | | | | |  |
|  |  | |  |  | | | ***Pregabalin*** | | |  |  |  |  | | | | | | | | | | | | | | | | |  | | | | | | | | |  |
| Feltner (2011) | 4 | Pregabalin (low dose) | 78 | 36.8 (10.1) | 41 | 300 (mg/day) | | Fixed | * | | LSAS >/= 50 | DSM-IV  Social Anxiety Disorder (Social Phobia), Generalized Subtype | | | DB Randomized  Parallel  Monotherapy | Outpatients | | 11 weeks | | Unclear | | | | | | Yes | | | | | |  | | |  |  |  |  |  |
|  |  | Pregabalin (medium dose) | 86 | 35.1 (12.1) | 41.9 | 450 (mg/day) | | Fixed | * | | LSAS >/= 50 |  |  |  |  |  |  |  |  |  |  |  |  |  |  |  |  |  |  |  |  |  | | |  |  |  |  |  |
|  |  | Pregabalin (high dose) | 82 | 35.1 (11.7) | 31.7 | 600 (mg/day) | | Fixed | * | | LSAS >/= 50 |  |  |  |  |  |  |  |  |  |  |  |  |  |  |  |  |  |  |  |  |  | | |  |  |  |  |  |
|  |  | Placebo | 82 | 34.6 (11.2) | 47.6 | 0-0 | | Fixed | 0 | | LSAS >/= 50 |  |  |  |  |  |  |  |  |  |  |  |  |  |  |  |  |  |  |  |  |  | | |  |  |  |  |  |
| Greist (2011) | 2 | Pregabalin | 80 | 34.4 (*) | 42.5 | 450 (mg/day) | | Fixed | * | | CGI-I </=2 & >/=25% reduction in LSAS from open-label baseline | DSM-IV  Social Anxiety Disorder (Social Phobia), Generalized Subtype | | | DB Randomized  Parallel  Monotherapy,  10 week open-label phase (LSAS >/= 50 for open-label), before 26-week DB phase. | Outpatients | | 26 weeks | | Unclear | | | | | | Yes | | | | | |  | | |  |  |  |  |  |
|  |  | Placebo | 73 | 36.5 (*) | 39.7 | 0-0 | | Fixed | 0 | | Same as above |  |  |  |  |  |  |  | |  |  |  |  |  |  |  | | | | | |  | | |  |  |  |  |  |
| Pande (2004) | 3 | Pregabalin | 42 | 36.3 (10.4) | 31 | 150 (mg/day) | | Fixed | * | | LSAS >/= 50 | DSM-IV  Social Anxiety Disorder (Social Phobia), Generalized Subtype | | | DB Randomized  Parallel  Monotherapy,  Placebo lead-in | Outpatients | | 10 weeks | | Unclear | | | | | | Yes | | | | | |  | | |  |  |  |  |  |
|  |  | Pregabalin | 47 | 37.9 (12.4) | 48.9 | 600 (mg/day) | | Fixed | * | | LSAS >/= 50 |  |  |  |  |  |  |  |  |  |  |  |  |  |  |  | | | | | |  | | |  |  |  |  |  |
|  |  | Placebo | 46 | 41 (11.2) | 43.5 | 0-0 | | Fixed | 0 | | LSAS >/= 50 |  |  |  |  |  |  |  |  | | | | |  | | | | | | |  | | | | | |  | | |
|  |  | |  |  | | | ***Gabapentin*** | | |  |  |  |  | | | | | | | | | | | | | | | | |  | | | | | | | | |  |
| Pande (1999) | 2 | Gabapentin | 34 | 35.6 (9.6) | * | 900-3600 (mg/day) | | Flexible | * | | LSAS >/= 50 | DSM-IV  Social Anxiety Disorder (Social Phobia) | | | DB Randomized  Parallel  Monotherapy,  Placebo lead-in | Outpatients | | 14 weeks | | Unclear | | | | | | Yes | | | | | |  | | |  |  |  |  |  |
|  |  | Placebo | 35 | 35.6 (9.6) | * | 0-0 | | Flexible | 0 | | LSAS >/= 50 |  |  |  |  |  |  |  |  | | | | |  | | | | | | |  | | | | | |  | | |
|  |  | |  |  | | | **Panic Disorder** | | |  |  |  |  | | | | | | | | | | | | | | | | |  | | | | | | | | |  |
|  |  | |  |  | | | ***Gabapentin*** | | |  |  |  |  | | | | | | | | | | | | | | | | |  | | | | | | | | |  |
| Pande (2000) | 2 | Gabapentin | 52 | 34.0 (10.6) | 58 | 600-3600 (mg/day) | | Flexible | * | | * | DSM-IV  Panic Disorder, with or without agoraphobia,  With at least one panic attack per week for the 3 weeks before screening | | | DB Randomized  Parallel  Monotherapy,  Placebo lead-in | * | | 8 weeks | | No | | | | | | Yes | | | | | |  | | |  |  |  |  |  |
|  |  | Placebo | 51 | 36.0 (9.4) | 69 | 0-0 | | Flexible | 0 | | * |  |  |  |  |  |  |  | |  |  |  |  |  |  |  | | | | | |  | | |  |  |  |  |  |
|  |  | |  |  | | | **Obsessive-Compulsive Disorder** | | |  |  |  |  | | | | | | | | | | | | | | | | |  | | | | | | | | |  |
|  |  | |  |  | | | ***Pregabalin*** | | |  |  |  |  | | | | | | | | | | | | | | | | |  | | | | | | | | |  |
| Mowla (2020) | 2 | Pregabalin  (+ sertraline) | 28 | 33.6 (11.3) | 65.3 | 75-225 (mg/day) | | Flexible | 185.9 | | * | DSM-V  Obsessive-Compulsive Disorder,  All patients had failed to respond to at least 12 weeks of treatment with an adequate and stable dose of sertraline. Concurrent sertraline continued throughout the trial. Mean dose of Sertraline 256.5 mg/day; dosage range 100-300mg/day | | | DB Randomized  Parallel  Adjunct | Outpatients | | 12 weeks | | Unclear | | | | | | No | | | | | |  | | |  |  |  |  |  |
|  |  | Placebo  (+ sertraline) | 28 | 31.1 (12.2) | 67.1 | 0-0 | | Flexible | 0 | | * |  |  |  |  |  |  |  |  |  |  |  |  |  |  |  | | | | | |  | | |  |  |  |  |  |

BP I = bipolar I disorder, BP II = bipolar II disorder

CGI-I = Clinical Global Impression-Improvement scale

CIWA-Ar = Clinical Institute Withdrawal Assessment for Alcohol-Revised

DB = double-blind; TAU = treatment as usual

DSM-IV = Diagnostic and Statistical Manual for Mental Disorders, 4^th^ edition

ESS = Epworth Sleepiness Scale

GA = General anesthesia

HAM-A = Hamilton Rating Scale for Anxiety

HAM-D = Hamilton Depression Rating Scale

IQR = Interquartile range

ISI = Insomnia Severity Index

LA = Local anesthesia

LSAS = Liebowitz Social Anxiety Scale

Med = Median

mg = milligrams

PSQI = Pittsburgh Sleep Quality Index

SD = standard deviation

YMRS = Young Mania Rating Scale

^a^ Malcolm 2007, number randomized patients total 101 combining both arms of the study. Authors were contacted, without success, for breakdown of numbers randomized across the arms.

* Missing

** NCT00551135:

- 50mg arm dosing: 25mg night before; 25mg 2+/-1 hour before surgery; then 25mg BD for 7 days
- 150mg arm dosing: 75mg night before; 75mg 2+/-1 hour before surgery; then 75mg BD for 7 days
- 300mg arm dosing: 150mg night before; 150mg 2+/-1 hour before surgery; then 150mg BD for 7 days

*** Rescue medications in pre-operative studies: Standard post-operative medications (e.g. analgesia, antiemetics, etc.) were not considered rescue medications when assessing pre-operative anxiety studies. Rescue medications were considered if they could plausibly confound patient anxiety pre-operatively.

**Table 3. Sensitivity analyses of Supplementary Figure 5 (pregabalin vs placebo in GAD, outcome HAM-A)**

| **Pregabalin vs placebo in generalized anxiety disorder, HAM-A** | **Reason for sensitivity analysis A-C** | **No. of studies** | **No. patients randomised** | **Mean difference (MD) in HAM-A** | **95% confidence interval (CI)** | **Heterogeneity (I^2^)** |
| --- | --- | --- | --- | --- | --- | --- |
| Random effects | Refer to supplementary Figure 5 | 10 | 2689 | -2.76 | -3.53, -2.00 | 37% |
| A | Exclusion of studies of pregabalin as add-on therapy. | 9 | 2336 | -3.14 | -3.84, -2.45 | 0% |
| B | Exclusion of studies with psychiatric comorbidities. | 6 | 1642 | -2.52 | -3.55, -1.48 | 42% |
| C | Exclusion of studies with rescue medications. | 9 | 2602 | -2.69 | -3.47, -1.92 | 38% |
| Fixed effects | Fixed effects analysis | 10 | 2689 | -2.50 | -3.06, -1.93 | 37% |

## Table 4. Sensitivity analyses of main text Figure 3: Gabapentin vs placebo in preoperative anxiety by dose subgroups

| Gabapentin vs placebo in pre-operative anxiety, by subgroups (dose=600mg and >600mg) | **Reason for sensitivity analyses A-C** | **Subgroup**  **(Low dose: 600mg;**  **High dose: >600mg)** | **No. of studies** | **No. patients randomized** | **Standardised mean difference (SMD)** | **95% confidence interval (CI)** | **Heterogeneity (I^2^)** |
| --- | --- | --- | --- | --- | --- | --- | --- |
| Random effects | Refer to Figure 3 in main text. | All doses | 13 | 817 | -0.92 | -1.32, -0.52 | 86% |
|  |  | Low dose | 4 | 244 | -0.06 | -0.32, 0.20 | 0% |
|  |  | High dose | 9 | 573 | -1.30 | -1.72, -0.87 | 81% |
| A | Sensitivity analysis – exclusion of studies that did not report baseline measures *or* reported group differences in baseline measures | All doses | 8 | 593 | -0.70 | -1.05, -0.34 | 76% |
|  |  | Low dose | 2 | 150 | -0.03 | -0.36, 0.30 | 0% |
|  |  | High dose | 6 | 443 | -0.91 | -1.21, -0.61 | 55% |
| B | Sensitivity analysis – exclusion of studies with high probability of skew (mean/SD <1). | All doses | 11 | 697 | -1.04 | -1.50, -0.59 | 87% |
|  |  | Low dose | 2 | 124 | 0.09 | -0.26, 0.44 | 0% |
|  |  | High dose | 9 | 573 | -1.30 | -1.72, -0.87 | 81% |
| C | Sensitivity analysis – exclusion of studies with extracted data reported in median/IQR/range. | All doses | 8 | 513 | -1.23 | -1.78, -0.67 | 87% |
|  |  | Low dose | 1 | 44 | 0.24 | -0.36, 0.83 | N/A |
|  |  | High dose | 7 | 469 | -1.42 | -1.94, -0.90 | 84% |
| Fixed effects | Fixed effects analysis | All doses | 13 | 817 | -0.74 | -0.89, -0.60 | 86% |
|  |  | Low dose | 4 | 244 | -0.06 | -0.32, 0.20 | 0% |
|  |  | High dose | 9 | 573 | -1.08 | -1.26, -0.90 | 81% |

## Table 5. Sensitivity analyses of main text Figure 4: Pregabalin vs placebo in preoperative anxiety by dose subgroups

| Pregabalin vs placebo in pre-operative anxiety, by subgroups (dose≤150mg and dose=300mg) | **Reason for sensitivity analysis, A-C** | **(Low dose, ≤150mg;**  **High dose, 300mg)** | **No. of studies** | **No. patients randomised** | **Standardised mean difference** | **95% confidence interval** | **Heterogeneity (I^2^)** |
| --- | --- | --- | --- | --- | --- | --- | --- |
| Random effects | Refer to Figure 4 in main text | All doses | 12 | 971 | -0.43 | -0.73, -0.13 | 77% |
|  |  | Low dose | 8 | 609 | -0.29 | -0.54, -0.05 | 45% |
|  |  | High dose | 4 | 362 | -0.74 | -1.65, 0.18 | 91% |
| A | Sensitivity analysis – exclusion of studies that did not report baseline measures *or* reported group differences in baseline measures | All doses | 11 | 925 | -0.38 | -0.69, -0.08 | 76% |
|  |  | Low dose | 7 | 563 | -0.20 | -0.38, -0.02 | 6% |
|  |  | High dose | 4 | 362 | -0.74 | -1.65, 0.18 | 91% |
| B | Sensitivity analysis – exclusion of studies with high probability of skew (mean/SD <1). | All doses | 10 | 698 | -0.51 | -0.88, -0.14 | 79% |
|  |  | Low dose | 6 | 336 | -0.39 | -0.70, -0.09 | 42% |
|  |  | High dose | 4 | 362 | -0.74 | -1.65, 0.18 | 91% |
| C | Sensitivity analysis – exclusion of studies with extracted data reported in median/IQR/range. | All doses | 11 | 925 | -0.46 | -0.78, -0.14 | 79% |
|  |  | Low dose | 7 | 563 | -0.31 | -0.59, -0.04 | 52% |
|  |  | High dose | 4 | 362 | -0.74 | -1.65, 0.18 | 91% |
| Fixed effects | Fixed effects analysis | All doses | 12 | 971 | -0.31 | -0.44, -0.17 | 77% |
|  |  | Low dose | 8 | 609 | -0.25 | -0.42, -0.09 | 45% |
|  |  | High dose | 4 | 362 | -0.40 | -0.62, -0.17 | 91% |

# **Table 6. Tolerability (table of side effects)**

| **Study name** | **Intervention** | **N** | | **N with SE** | | **Amnesia** | **Anxiety** | **Convulsion** | **Depression** | **Dizziness** | **Drowsiness** | **Emotional lability** | **Euphoria** | **Insomnia** | |  | | **Movement disorder** | | | **Vertigo** | | | | **Visual problems** | | | | **Weight gain** | | | | **Other** | | | |
| --- | --- | --- | --- | --- | --- | --- | --- | --- | --- | --- | --- | --- | --- | --- | --- | --- | --- | --- | --- | --- | --- | --- | --- | --- | --- | --- | --- | --- | --- | --- | --- | --- | --- | --- | --- | --- |
| **BIPOLAR DISORDER** | | | | | | | | | | | | | | | | | | | | | | | | | | | | | | | | | | | |  |
| **RANDOMISED DOUBLE-BLIND TRIALS** | | | | | | |  |  |  |  |  |  |  | |  | |  | |  | | |  | | | |  | | | |  | | | |  | | |
| ***GABAPENTIN*** | |  | |  | |  |  |  |  |  |  |  |  | |  | |  | |  | | | |  | | | |  | | | |  | | | |  | |
| Frye (2000) (NCT00001482) | Gabapentin 900-4800 mg/day | 9 | | * | | * | * | * | * | * | * | * | * | | * | |  | | * | | | | * | | | | * | | | | * | | | | * | |
|  | Lamotrigine 25-500 mg/day | 10 | | * | | * | * | * | * | * | * | * | * | | * | |  | | * | | | | * | | | | * | | | | * | | | | * | |
|  | Placebo | 11 | | * | | * | * | * | * | * | * | * | * | | * | |  | | * | | | | * | | | | * | | | | * | | | | * | |
| Mokhber (2008) | Gabapentin 300-900 mg/day | 18 | | 2 | | * | * | * | * | * | * | * | * | | * | |  | | * | | | | * | | | | * | | | | * | | | | * | |
|  | Carbamazepine 400-600 mg/day | 13 | | 6 | | * | * | * | * | * | * | * | * | | * | |  | | * | | | | * | | | | * | | | | * | | | | * | |
|  | Lamotrigine 25-100 mg/day | 20 | | 0 | | * | * | * | * | * | * | * | * | | * | |  | | * | | | | * | | | | * | | | | * | | | | * | |
| Pande (2000)    (945-209) | Gabapentin 600-3600 mg/day (Adjunctive therapy with Lithium, Valproate or combination) | 59 | | 45 | | 6 | 0 | 0 | 1 | 11 | 14 | 0 | 0 | | 1 | |  | | 3 | | | | 0 | | | | 4 | | | | 2 | | | | Abdominal pain=2; Acne=2; Abnormal ejaculation=2; Abnormal gait=4; Accidental injury=2; Alopecia=1; Anorexia=1; Arthralgia=1; Asthenia=2; Asthma=1; Back pain=2; Chest pain=2; Chills=1; Confusion=1; Constipation=3; Diarrhea=9; Dry mouth=5; Dry skin=1; Indigestion=1; Dysphagia=1; Dyspnea=2; Flu syndrome=2; Hair Disorder=1; Headache=6; Hypotonia=1; Impotence=1; Incoordination=2; Increased Appetite=5; Infection=1; Manic Reaction=2; Nausea=4; Neck Pain=1; Nervousness=1; Nystagmus=1; Pain=1; Papancolau Smear Susupicious=1; Paranoid reaction=1; Peripheral Edema=4; Pharyngitis=2; Polyurea=2; Postural Hypotension=1; Rhinitis=2; Sinusitis=1; Speech Disorder=2; Thirst=3; Urinary Frequency=1; Urinary Incontinence=2; Urinary Tract Infection=1; Urinary Urgency=2; Vasodilatation=1; Vomiting=1 | |
|  | Placebo (Adjunctive therapy with Lithium, Valproate or combination) | 59 | | 39 | | 2 | 0 | 0 | 0 | 3 | 7 | 0 | 0 | | 1 | |  | | 7 | | | | 0 | | | | 1 | | | | 4 | | | | Abdominal pain=4; Acne=1; Abnormal Dreams=1; Abnormal stools=1; Accidental injury=2; Alopecia=1; Anorexia=1; Arthralgia=1; Asthenia=3; Back pain=3; Balanitis=1; Confusion=2; Constipation=2; Cough Increased=1; Diarrhea=7; Dry mouth=3; Dry skin=1; Indigestion=4; Ecchymosis=1; Face Edema=1; Flu syndrome=3; Generalised Edema=1; Headache=7; Hypothyroidism=1; Impotence=1; Incoordination=2; Increased Appetite=4; Infection=6; Libido Decreased=1; Manic Reaction=5; Nausea=6; Neck Pain=1; Neuropathy=1; Pain=2; Palpitation=1; Parasthesia=1; Pericarditus=1; Peripheral Edema=3; Pharyngitis=1; Photophobia=1; Pleural Disorder=1; Pneumonia=1; Psoriasis=1; Psychosis=1; Pruritis=1; Rash=1; Rhinitis=2; Sinusitis=1; Thinking Abnormal=2; Thirst=1; Tinnitus=1; Tongue Disorder=1; Twitching=1; Urinary Tract Infection=1; Vasodilatation=1; Vomiting=1 | |
| Vieta_2006           (945-291) | Gabapentin 1200-2400 mg/day (Adjunctive therapy with Lithium, Valproate, Carbamazepine or combination) | 13 | | 10 | | * | * | * | * | 2 | * | * | * | | 2 | |  | | 2 | | | | * | | | | * | | | | * | | | | Constipation=4; Headache=3; Nausea=3 | |
|  | Placebo (Adjunctive therapy with Lithium, Valproate, Carbamazepine or combination) | 12 | | 7 | | * | * | * | * | * | * | * | * | | * | |  | | * | | | | * | | | | * | | | | * | | | | * | |
| **OPEN LABEL TRIALS** | | |  | |  |  |  |  |  |  |  |  |  | |  | |  | |  | | |  | | | |  | | | |  | | | |  | | |
| ***GABAPENTIN*** | |  | |  | |  |  |  |  |  |  |  |  | |  | |  | |  | | | |  | | | |  | | | |  | | | |  | |
| Altshuler 1999 | Gabapentin 300-3600 mg/day (Adjunctive therapy to TAU) | 28 | | 12 | | 0 | 0 | 0 | 0 | 1 | 0 | 0 | 0 | | 0 | |  | | 2 | | | | 0 | | | | 0 | | | | 0 | | | | Gastrointestinal upset=4; Headache=1; Sedation=5 | |
| Astaneh 2012 | Lithium | 30 | | * | | * | * | * | * | * | * | * | * | | * | |  | | * | | | | * | | | | * | | | | * | | | | * | |
|  | Lithium plus Gabapentin 900 mg/day | 30 | | * | | * | * | * | * | * | * | * | * | | * | |  | | * | | | | * | | | | * | | | | * | | | | * | |
| Erfurth 1998 | Gabapentin  (Adjunctive therapy to TAU) | 6 | | * | | * | * | * | * | * | * | * | * | | * | |  | | * | | | | * | | | | * | | | | * | | | | * | |
|  | Gabapentin 1200-3600 mg/day | 8 | | * | | * | * | * | * | * | * | * | * | | * | |  | | * | | | | * | | | | * | | | | * | | | | * | |
| Knoll 1998 | Gabapentin 300-3300 mg/day (Adjunctive therapy to TAU) | 12 | | 11 | | 0 | 0 | 0 | 0 | 0 | 0 | 0 | 0 | | 0 | |  | | 3 | | | | 0 | | | | 0 | | | | 1 | | | | Alopecia=1; Fatigue=2; Irritability=3; Nausea=1; Sedation=5 | |
| Mauri 2001 | Gabapentin 300-2400 mg/day | 21 | | * | | * | * | * | * | * | * | * | * | | * | |  | | * | | | | * | | | | * | | | | * | | | | * | |
| McElroy 1997 | Gabapentin 300-4800 mg/day (Adjunctive therapy to TAU) | 9 | | 7 | | 0 | 0 | 0 | 0 | 0 | 0 | 0 | 0 | | 0 | |  | | 2 | | | | 0 | | | | 1 | | | | 0 | | | | Constipation=1; Dark urine=1; Episodic disorientation=1; Forgetfullness=1; Migraine=1; Sedation=7; Memory loss=2 | |
| Perugi 1999 | Gabapentin 300-2000 mg/day (Adjunctive therapy to TAU) | 21 | | * | | * | * | * | * | * | * | * | * | | * | |  | | 7 | | | | * | | | | * | | | | * | | | | Irritability=5; Nausea=3; Sedation=9 | |
| Wang 2002 | Gabapentin 600-3300 mg/day (Adjunctive therapy to stable doses of mood stabilisers or typical antipsychotics) | 22 | | * | | * | * | * | * | * | * | * | * | | * | |  | | * | | | | * | | | | * | | | | * | | | | Hypomanic symptoms=3; Impaired cognition=1; Sedation=7 | |
| Young 1997 | Gabapentin 300-2400 mg/day (Adjunctive therapy to TAU) | 15 | | * | | 0 | 3 | 0 | 0 | 3 | 0 | 0 | 0 | | 0 | |  | | 3 | | | | 0 | | | | 0 | | | | 2 | | | | Gasrointestinal upset=13; Incoordination=1; Sedation=6 | |
| Young 1999 | Gabapentin 300-3600 mg/day (Adjunctive therapy to TAU) | 37 | | * | | * | 9 | * | * | * | 8 | * | * | | * | |  | | * | | | | * | | | | 5 | | | | * | | | | Constipation=4; Dry mouth=6; Sexual difficulties=9; Sleep problems=7 | |
| ***PREGABALIN*** | |  | |  | |  |  |  |  |  |  |  |  | |  | |  | |  | | | |  | | | |  | | | |  | | | |  | |
| Schaffer 2012 | Pregabalin (Adjunctive therapy to TAU) | 58 | | * | | * | * | * | * | * | * | * | * | | * | |  | | * | | | | * | | | | * | | | | 8 | | | | Increased appetite=8; Overactivation=12 | |
| **INSOMNIA/SLEEP DISTURBANCE** | | | | | | | | | | | | | | | | | | | | | | | | | | | | | | | | | | | |  |
| **RANDOMISED DOUBLE-BLIND TRIALS** | | | | | |  |  |  |  |  |  |  |  |  | |  | |  | |  | | | |  | | | |  | | | |  | | | | |
| ***GABAPENTIN*** | |  | |  | |  |  |  |  |  |  |  |  |  | |  | |  | | |  | | | |  | | | |  | | | |  | | | |
| Brower 2008 | Gabapentin 300-1500 mg/day | 10 | | 10 | | 0 | 0 | 0 | 0 | 2 | 3 | 0 | 0 | 0 | |  | | 1 | | | 0 | | | | 0 | | | | 0 | | | | Abdominal pain=1; Constipation=1; Diarrhea=1; Dry mouth=1; Headache=3; Indigestion=2; Memory disturbance=1; Menstrual cramps=1; Nerve/muscle pain=2; Paleness=1; Twitching/tingling=2 | | | |
|  | Placebo | 11 | | 10 | | 0 | 0 | 0 | 0 | 1 | 1 | 0 | 0 | 0 | |  | | 1 | | | 0 | | | | 0 | | | | 0 | | | | Altered mental state=2; Headache=3; Indigestion=4; Memory disturbance=1; Twitching/tingling=1 | | | |
| Furey 2014 | Gabapentin 250 mg/day | 128 | | 13 | | 0 | 0 | 0 | 0 | 1 | 4 | 0 | 0 | 0 | |  | | 0 | | | 0 | | | | 0 | | | | 0 | | | | Diarrhea=1; Dry mouth=1; Headache=4; Nausea=1; Sluggishness=1 | | | |
|  | Placebo | 128 | | 10 | | 0 | 1 | 0 | 0 | 0 | 1 | 0 | 0 | 0 | |  | | 0 | | | 1 | | | | 1 | | | | 0 | | | | Headache=3; Hypertension=1; Impotence=1; Musculoskeletal stiffness=1; Restlessness=1 | | | |
| Malcolm 2007 | Gabapentin 600-1200 mg/day | 101 total participants randomised† | | * | | * | * | * | * | * | * | * | * | * | |  | | * | | | * | | | | * | | | | * | | | | * | | | |
|  | Lorazepam 6 mg/day |  |  | * | | * | * | * | * | * | * | * | * | * | |  | | * | | | * | | | | * | | | | * | | | | * | | | |
| Mason 2014 (NCT00391716) | Gabapentin 1800 mg/day | 47 | | 1 | | * | * | * | * | * | * | * | 0 | 6 | |  | | * | | | * | | | | * | | | | * | | | | Headache=6; Fatigue=9 | | | |
|  | Gabapentin 900 mg/day | 54 | | 3 | | * | * | * | * | * | * | * | 0 | 10 | |  | | * | | | * | | | | * | | | | * | | | | Headache=7; Fatigue=13 | | | |
|  | Placebo | 49 | | 1 | | * | * | * | * | * | * | * | 1 | 11 | |  | | * | | | * | | | | * | | | | * | | | | Headache=8; Fatigue=12 | | | |
| Mowla 2015 | Gabapentin 100-600 mg/day (adjunctive to TAU for MDD) | 31 | | * | | * | * | * | * | * | * | * | * | * | |  | | * | | | * | | | | * | | | | * | | | | * | | | |
|  | Clonazepam .5-2 mg/day (adjunctive to TAU for MDD) | 32 | | * | | * | * | * | * | * | * | * | * | * | |  | | * | | | * | | | | * | | | | * | | | | * | | | |
| NCT01014533 (HUM00010947 1R01AA016117-01A1) | Gabapentin 600-1200 mg/day | 30 | | 4 | | 0 | 0 | 0 | 0 | 0 | 0 | 0 | 0 | 0 | |  | | 0 | | | 0 | | | | 0 | | | | 0 | | | | Cough Increased=2; Flu Syndrome=2 | | | |
|  | Placebo | 29 | | 2 | | 0 | 0 | 0 | 0 | 0 | 0 | 0 | 0 | 0 | |  | | 0 | | | 0 | | | | 0 | | | | 0 | | | | Irritability=2 | | | |
| Rosenburg 2013 (NCT006747552) | Gabapentin 500 mg single dose | 125 | | 5 | | 0 | 0 | 0 | 0 | 1 | 0 | 0 | 1 | 0 | |  | | 0 | | | 0 | | | | 0 | | | | 0 | | | | Altered mental state=1; Headache=2; Nausea=1 | | | |
|  | Gabapentin 250 mg single dose | 125 | | 4 | | 0 | 1 | 0 | 0 | 0 | 0 | 0 | 0 | 0 | |  | | 0 | | | 0 | | | | 0 | | | | 0 | | | | Headache=1; Hypertension=2; Sinusitis=1 | | | |
|  | Placebo | 127 | | 2 | | 0 | 0 | 0 | 0 | 0 | 0 | 0 | 0 | 0 | |  | | 0 | | | 0 | | | | 0 | | | | 0 | | | | Asthma=1; Headache=1; Nausea=1; Vomiting=1 | | | |
| Yurcheshen 2009 | Gabapentin 900 mg/day | 30 | | 15 | | * | * | * | * | 4 | 6 | * | * | * | |  | | * | | | * | | | | * | | | | * | | | | Menses=2; Rash=2 | | | |
|  | Placebo | 29 | | 8 | | * | * | * | * | 0 | 0 | * | * | * | |  | | * | | | * | | | | * | | | | * | | | | Menses=3 | | | |
| ***PREGABALIN*** | |  | |  | |  |  |  |  |  |  |  |  |  | |  | |  | | |  | | | |  | | | |  | | | |  | | | |
| Bollu 2010 | Pregabalin 300-600 mg/day | 121 | | * | | * | * | * | * | * | * | * | * | * | |  | | * | | | * | | | | * | | | | * | | | | * | | | |
|  | Venlafaxine 75-225 mg/day | 125 | | * | | * | * | * | * | * | * | * | * | * | |  | | * | | | * | | | | * | | | | * | | | | * | | | |
|  | Placebo | 128 | | * | | * | * | * | * | * | * | * | * | * | |  | | * | | | * | | | | * | | | | * | | | | * | | | |
| **OPEN LABEL TRIALS** | | | | | | | | | | | | | | | | | | | | | | | | | | | | | | | | | | | |  |
| ***GABAPENTIN*** | |  | |  | |  |  |  |  |  |  |  |  |  | |  | |  | | |  | | | |  | | | |  | | | |  | | | |
| NCT02040532 | Gabapentin 100-600 mg/day | 26 | | 11 | | 0 | 0 | 0 | 0 | 0 | 8 | 0 | 0 | 4 | |  | | 0 | | | 0 | | | | 0 | | | | 4 | | | | Abdominal pain=4; Cough Increased =4; Fatigue=6; Flatulence=4; Flu syndrome=6; Headache=9; Hot flushes=7; Increased libido=4; Musculoskeletal stiffness=4; Nausea=3; Rash=1; Sweating=8; Tension=4 | | | |
| **GENERALISED ANXIETY DISORDER** | | | | | | | | | | | | | | | | | | | | | | | | | | | | | | | | | | | |  |
| **RANDOMISED DOUBLE-BLIND TRIALS** | | | | | |  |  |  |  |  |  |  |  |  | |  | |  | |  | | | |  | | | |  | | | |  | | | | |
| ***PREGABALIN*** | |  | |  | |  |  |  |  |  |  |  |  |  | |  | |  | | |  | | | |  | | | |  | | | |  | | | |
| Feltner 2003 | Pregabalin 150 mg/day | 70 | | 51 | | 2 | * | * | * | 7 | 20 | * | * | * | |  | | * | | | * | | | | 0 | | | | * | | | | Accidental injury=2; Diarrhea=3; Dry mouth=8; Fatigue =5; Headache=7; Infection=6; Nausea=7; Thinking abnormal/confusion=1 | | | |
|  | Pregabalin 600 mg/day | 66 | | 59 | | 2 | * | * | * | 19 | 33 | * | * | * | |  | | * | | | * | | | | 5 | | | | * | | | | Accidental injury=4; Diarrhea=2; Dry mouth=10; Fatigue =1; Headache=12; Infection=4; Incoordination=1; Nausea=4; Thinking abnormal/confusion=1; Rhinitis=4 | | | |
|  | Placebo | 67 | | 45 | | 0 | * | * | * | 5 | 8 | * | * | * | |  | | * | | | * | | | | 0 | | | | * | | | | Accidental injury=2; Diarrhea=5; Dry mouth=7; Fatigue =4; Headache=6; Infection=6; Nausea=5; Thinking abnormal/confusion=1 | | | |
|  | Lorazepam 6 mg/day | 68 | | 62 | | 4 | * | * | * | 13 | 41 | * | * | * | |  | | * | | | * | | | | 1 | | | | * | | | | Accidental injury=1; Dry mouth=5; Fatigue =7; Headache=5; Infection=1; Incoordination=5; Nausea=11; Thinking abnormal/confusion=6; Rhinitis=1 | | | |
| Feltner 2008 | Pregabalin 450 mg/day | 168 | | * | | * | * | * | * | 7 | 10 | * | 0 | * | |  | | * | | | * | | | | * | | | | 8 | | | | Dry mouth=6; Headache=17; Infection=25; Incoordination=2; Thinking abnormal/confusion=3 | | | |
|  | Placebo | 170 | | * | | * | * | * | * | 5 | 0 | * | 0 | * | |  | | * | | | * | | | | * | | | | 0 | | | | Dry mouth=2; Headache=19; Infection=19 | | | |
| Hadley 2012 (NCT00368745) | Pregabalin 150-600 mg/day | 56 | | 33 | | * | 11 | * | * | 12 | 3 | * | * | 4 | |  | | 2 | | | * | | | | 5 | | | | * | | | | Diarrhea=3; Fatigue=5; Headache=7; Hyperacusis=3; Hyperhidrosis=3; Nausea=5; Pain=3; Paraesthesia=6; Peripheral oedema=5; Vomiting=3 | | | |
|  | Placebo | 50 | | 29 | | * | 10 | * | * | 3 | 2 | * | * | 7 | |  | | 4 | | | * | | | | 2 | | | | * | | | | Diarrhea=5; Fatigue=8; Headache=13; Hyperacusis=1; Irritability=4; Nausea=7 | | | |
| Kasper 2009 (NCT00151450) | Pregabalin 300-600 mg/day | 121 | | * | | * | * | * | * | 25 | 11 | * | * | 5 | |  | | * | | | 16 | | | | * | | | | 2 | | | | Constipation=5; Dry mouth=13; Fatigue=12; Headache=21; Hyperhidrosis=3; Nausea=15 | | | |
|  | Venlafaxine 75-225 mg/day | 125 | | * | | * | * | * | * | 12 | 6 | * | * | 12 | |  | | * | | | 10 | | | | * | | | | 1 | | | | Constipation=7; Dry mouth=15; Fatigue=16; Headache=20; Hyperhidrosis=10; Nausea=32 | | | |
|  | Placebo | 128 | | * | | * | * | * | * | 8 | 3 | * | * | 6 | |  | | * | | | 4 | | | | * | | | | 1 | | | | Constipation=4; Dry mouth=5; Fatigue=5; Headache=15; Hyperhidrosis=7; Nausea=11 | | | |
| Kasper 2014 (NCT00624780) | Pregabalin 450-600 mg/day | 206 | | * (SEs not reported for DB period) | | * | * | * | * | * | * | * | * | * | |  | | * | | | * | | | | * | | | | * | | | | * | | | |
|  | Pregabalin 150-300 mg/day | 206 | | * (SEs not reported for DB period) | | * | * | * | * | * | * | * | * | * | |  | | * | | | * | | | | * | | | | * | | | | * | | | |
|  | Lorazepam 3-4 mg/day | 206 | | * (SEs not reported for DB period) | | * | * | * | * | * | * | * | * | * | |  | | * | | | * | | | | * | | | | * | | | | * | | | |
| Montgomery 2006 | Pregabalin 400 mg/day | 97 | | * | | * | * | * | * | 22 | 13 | * | * | 1 | |  | | * | | | * | | | | * | | | | * | | | | Constipation=7; Diarrhea=4; Dry mouth=5; Fatigue=5; Headache=7; Infection=9; Nausea=9; Vomiting=4 | | | |
|  | Pregabalin 600 mg/day | 110 | | * | | * | * | * | * | 29 | 15 | * | * | 3 | |  | | * | | | * | | | | * | | | | * | | | | Constipation=7; Diarrhea=5; Dry mouth=5; Fatigue=4; Headache=9; Infection=3; Nausea=14; Vomiting=2 | | | |
|  | Venlafaxine 75 mg/day | 113 | | * | | * | * | * | * | 14 | 4 | * | * | 8 | |  | | * | | | * | | | | * | | | | * | | | | Constipation=7; Diarrhea=5; Dry mouth=8; Fatigue=14; Headache=10; Infection=3; Nausea=31; Vomiting=9 | | | |
|  | Placebo | 101 | | * | | * | * | * | * | 7 | 3 | * | * | 5 | |  | | * | | | * | | | | * | | | | * | | | | Constipation=2; Diarrhea=6; Dry mouth=2; Fatigue=6; Headache=13; Infection=4; Nausea=8; Vomiting=5 | | | |
| Montgomery 2008 (EUCTR2004-000955-40-LV) | Pregabalin 150-600 mg/day | 181 | | 127 | | * | * | * | * | 36 | 23 | * | * | 5 | |  | | 5 | | | 4 | | | | 5 | | | | 5 | | | | Abdominal pain=7; Accidental injury=7; Back pain=4; Constipation=5; Diarrhea=4; Dry mouth=6; Fatigue=5; Headache=18; Hypertension=7; Infection=10; Incoordination=2; Indigestion=4; Nausea=16; Pain=1; Peripheral Edema=7; Paresthesia=5; Thinking abnormal=1 | | | |
|  | Placebo | 96 | | 54 | | * | * | * | * | 11 | 7 | * | * | 6 | |  | | 0 | | | 0 | | | | 1 | | | | 0 | | | | Abdominal pain=3; Accidental injury=1; Back pain=5; Constipation=2; Diarrhea=5; Dry mouth=2; Fatigue=3; Headache=8; Hypertension=1; Infection=3; Nausea=6; Pain=4; Peripheral Edema=3; Thinking abnormal=2 | | | |
| Pande 2003 | Pregabalin 150 mg/day | 69 | | * | | 1 | * | * | * | 16 | 10 | * | * | 2 | |  | | 0 | | | * | | | | 4 | | | | * | | | | Constipation=1; Depersonalisation=1; Diarrhea=2; Dry mouth=6; Fatigue=4; Headache=13; Infection=8; Nausea=5; Pain=4; Thinking abnormal=2; Vomiting=1 | | | |
|  | Pregabalin 600 mg/day | 70 | | * | | 5 | * | * | * | 27 | 25 | * | * | 1 | |  | | 7 | | | * | | | | 8 | | | | * | | | | Constipation=5; Depersonalisation=6; Diarrhea=8; Dry mouth=11; Fatigue=6; Headache=15; Infection=3; Incoordination=8; Nausea=6; Pain=1; Thinking abnormal=9; Vomiting=2 | | | |
|  | Lorazepam 6 mg/day | 68 | | * | | 3 | * | * | * | 9 | 37 | * | * | 4 | |  | | 8 | | | * | | | | 1 | | | | * | | | | Constipation=3; Depersonalisation=4; Diarrhea=2; Dry mouth=3; Fatigue=11; Headache=6; Infection=2; Incoordination=13; Nausea=6; Thinking abnormal=6; Vomiting=1 | | | |
|  | Placebo | 69 | | * | | 3 | * | * | * | 4 | 8 | * | * | 1 | |  | | 0 | | | * | | | | 1 | | | | * | | | | Constipation=2; Diarrhea=2; Dry mouth=2; Fatigue=4; Headache=9; Infection=9; Incoordination=2; Nausea=7; Pain=1; Thinking abnormal=1; Vomiting=5 | | | |
| Pohl 2005 | Pregabalin 200 mg/day | 78 | | * | | * | * | * | * | 27 | 24 | * | 8 | * | |  | | * | | | * | | | | 5 | | | | * | | | | Dry mouth=19; Flatulence=8; Infection=13; Incoordination=3; Thinking abnormal=6 | | | |
|  | Pregabalin 400 mg/day | 89 | | * | | * | * | * | * | 44 | 33 | * | 9 | * | |  | | * | | | * | | | | 10 | | | | * | | | | Dry mouth=24; Flatulence=2; Infection=11; Incoordination=14; Thinking abnormal=12 | | | |
|  | Pregabalin 450 mg/day | 88 | | * | | * | * | * | * | 37 | 21 | * | 13 | * | |  | | * | | | * | | | | 12 | | | | * | | | | Dry mouth=15; Flatulence=11; Infection=9; Incoordination=12; Thinking abnormal=5 | | | |
|  | Placebo | 86 | | * | | * | * | * | * | 13 | 11 | * | 1 | * | |  | | * | | | * | | | | 3 | | | | * | | | | Dry mouth=9; Flatulence=2; Infection=6; Incoordination=2; Thinking abnormal=2 | | | |
| Rickels 2005 | Pregabalin 300 mg/day | 91 | | * | | * | * | * | * | 37 | 35 | * | * | * | |  | | * | | | * | | | | 8 | | | | * | | | | Constipation=2; Dry mouth=18; Fatigue=7; Infection=10; Incoordination=4; Nausea=10 | | | |
|  | Pregabalin 300-450 mg/day | 90 | | * | | * | * | * | * | 34 | 36 | * | * | * | |  | | * | | | * | | | | 10 | | | | * | | | | Constipation=12; Dry mouth=16; Fatigue=10; Infection=14; Incoordination=11; Nausea=13 | | | |
|  | Pregabalin 300-600 mg/day | 89 | | * | | * | * | * | * | 35 | 37 | * | * | * | |  | | * | | | * | | | | 8 | | | | * | | | | Constipation=3; Dry mouth=21; Fatigue=7; Infection=15; Incoordination=15; Nausea=10 | | | |
|  | Alprazolam 0.5-1.5 mg/day | 93 | | * | | * | * | * | * | 15 | 42 | * | * | * | |  | | * | | | * | | | | 4 | | | | * | | | | Constipation=3; Dry mouth=4; Fatigue=13; Infection=8; Incoordination=3; Nausea=9 | | | |
|  | Placebo | 91 | | * | | * | * | * | * | 9 | 15 | * | * | * | |  | | * | | | * | | | | 3 | | | | * | | | | Constipation=5; Dry mouth=8; Fatigue=2; Infection=9; Nausea=10 | | | |
| Rickels 2012 (NCT00413010) | Pregabalin 150-600 mg/day (Adjunctive to SSRI/SNRI) | 180 | | 81 | | * | * | * | * | 21 | 22 | * | 4 | 5 | |  | | * | | | * | | | | 8 | | | | 4 | | | | Cold=5; Constipation=5; Cough=2; Diarrhea=7; Dry mouth=4; Fatigue=7; Headache=17; Indigestion=6; Flatulence=4; Infection=3; Joint pain= 5; Nausea=13; Paraesthesia=4; Vomiting=2 | | | |
|  | Placebo (Adjunctive to SSRI/SNRI) | 176 | | 59 | | * | * | * | * | 10 | 15 | * | 0 | 1 | |  | | * | | | * | | | | 2 | | | | 0 | | | | Cold=6; Constipation=3; Cough=6; Diarrhea=7; Dry mouth=2; Fatigue=5; Headache=7; Indigestion=1; Infection=6; Joint pain= 1; Nausea=8; Paraesthesia=1; Vomiting=4 | | | |
| **OPEN LABEL TRIALS** | | |  | |  |  |  |  |  |  |  |  |  |  | |  | |  | |  | | | |  | | | |  | | | |  | | | | |
| ***PREGABALIN*** | |  | |  | |  |  |  |  |  |  |  |  |  | |  | |  | | |  | | | |  | | | |  | | | |  | | | |
| Cvjetkovic-Bosnjak 2015 | Pregabalin 225 mg/day | 47 | | * | | * | * | * | * | 6 | 5 | * | * | 0 | |  | | * | | | 1 | | | | * | | | | * | | | | Nausea=2 | | | |
|  | Sertraline 150 mg/day | 60 | | * | | * | * | * | * | 3 | 0 | * | * | 2 | |  | | * | | | 1 | | | | * | | | | * | | | | Diarrhea=3; Nausea=8 | | | |
| **SOCIAL ANXIETY DISORDER** | | | | | | | | | | | | | | | | | | | | | | | | | | | | | | | | | | | |  |
| **RANDOMISED DOUBLE-BLIND TRIALS** | | | | | |  |  |  |  |  |  |  |  |  | |  | |  | |  | | | |  | | | |  | | | |  | | | | |
| ***GABAPENTIN*** | |  | |  | |  |  |  |  |  |  |  |  |  | |  | |  | | |  | | | |  | | | |  | | | |  | | | |
| Pande 1999 | Gabapentin 900-3600 mg/day | 34 | | * | | * | 2 | * | * | 8 | 7 | * | * | 3 | |  | | * | | | * | | | | 2 | | | | 2 | | | | Decreased libido=3; Diarrhea=4; Dry mouth=4; Fatigue=5; Flatulence=3; Headache=8; Increased appetite=3; Infection=10; Nausea=4; Nervousness=5; Rhinitis/Sinusitis=5; Thinking abnormal=4; Vasodilation=2 | | | |
|  | Placebo | 35 | | * | | * | 0 | * | * | 2 | 3 | * | * | 3 | |  | | * | | | * | | | | 1 | | | | 4 | | | | Diarrhea=2; Fatigue=3; Headache=9; Infection=8; Increased appetite=2; Nausea=1; Nervousness=4; Rhinitis/Sinusitis=1; Thinking abnormal=2; Vasodilation=1 | | | |
| ***PREGABALIN*** | |  | |  | |  |  |  |  |  |  |  |  |  | |  | |  | | |  | | | |  | | | |  | | | |  | | | |
| Feltner 2011 | Pregabalin 300 mg/day | 78 | | 68 | | 8 | * | * | * | 27 | 29 | * | * | * | |  | | * | | | * | | | | * | | | | 4 | | | | Dry mouth=7; Fatigue=8; Headache=10; Infection=4; Nausea=4; Thinking abnormal=5 | | | |
|  | Pregabalin 450 mg/day | 86 | | 80 | | 3 | * | * | * | 34 | 31 | * | * | * | |  | | * | | | * | | | | * | | | | 6 | | | | Dry mouth=12; Fatigue=3; Headache=12; Infection=10; Nausea=11; Thinking abnormal=9 | | | |
|  | Pregabalin 600 mg/day | 82 | | 76 | | 6 | * | * | * | 30 | 39 | * | * | * | |  | | * | | | * | | | | * | | | | 10 | | | | Dry mouth=11; Fatigue=10; Headache=9; Infection=10; Nausea=9; Thinking abnormal=15 | | | |
|  | Placebo | 82 | | 49 | | 0 | * | * | * | 5 | 9 | * | * | * | |  | | * | | | * | | | | * | | | | 0 | | | | Dry mouth=1; Fatigue=1; Headache=11; Infection=10; Nausea=5; Thinking abnormal=1 | | | |
| Griest 2011 | Pregabalin 450 mg/day | 80 | | * | | * | * | * | 8 | 9 | 2 | * | * | 20 | |  | | * | | | * | | | | * | | | | * | | | | Headache=11; Infection=17; Nausea=9; Thinking abnormal=1 | | | |
|  | Placebo | 73 | | * | | * | * | * | 8 | 3 | 1 | * | * | 21 | |  | | * | | | * | | | | * | | | | * | | | | Headache=17; Infection=12; Nausea=7; Thinking abnormal=2 | | | |
| Pande 2004 | Pregabalin 150 mg/day | 42 | | 34 | | 1 | * | * | 0 | 5 | 4 | * | * | 3 | |  | | 0 | | | * | | | | 1 | | | | * | | | | Anorgasmia=1; Depersonalisation=2; Dry mouth=3; Fatigue=6; Headache=12; Infection=7; Nausea=3; Pain=4; Thinking abnormal=1 | | | |
|  | Pregabalin 600 mg/day | 47 | | 47 | | 5 | * | * | 3 | 19 | 20 | * | * | 4 | |  | | 3 | | | * | | | | 5 | | | | * | | | | Accidental injury=4; Anorgasmia=3; Decreased libido=4 Depersonalisation=4; Dry mouth=3; Fatigue=9; Headache=10; Infection=3; Incoordination=4; Nausea=4; Nervousness=6; Pain=3; Thinking abnormal=10 | | | |
|  | Placebo | 46 | | 40 | | 2 | * | * | 1 | 4 | 4 | * | * | 6 | |  | | 0 | | | * | | | | 0 | | | | * | | | | Dry mouth=1; Fatigue=3; Headache=11; Infection=9; Nausea=2; Nervousness=4; Pain=1; Thinking abnormal=3 | | | |
| **PANIC DISORDER** | | | | | | | | | | | | | | | | | | | | | | | | | | | | | | | | | | | |  |
| **RANDOMISED DOUBLE-BLIND TRIALS** | | | | | |  |  |  |  |  |  |  |  |  | |  | |  | |  | | | |  | | | |  | | | |  | | | | |
| ***GABAPENTIN*** | |  | |  | |  |  |  |  |  |  |  |  |  | |  | |  | | |  | | | |  | | | |  | | | |  | | | |
| Pande 2000 | Gabapentin 600-3600 mg/day | 52 | | * | | * | * | * | * | 12 | 16 | * | * | * | |  | | 6 | | | * | | | | * | | | | * | | | | Fatigue=9; Headache=14; Indigestion=5; Infection=11; Nausea=6 | | | |
|  | Placebo | 51 | | * | | * | * | * | * | 6 | 9 | * | * | * | |  | | 1 | | | * | | | | * | | | | * | | | | Fatigue=7; Headache=12; Indigestion=7; Infection=7; Nausea=8 | | | |
| **GENERALISED ANXIETY DISORDER, SOCIAL ANXIETY DISORDER AND PANIC DISORDER** | | | | | | | | | | | | | | | | | | | | | | | | | | | | | | | | | | | |  |
| **OPEN LABEL TRIALS** | | |  | |  |  |  |  |  |  |  |  |  |  | |  | |  | |  | | | |  | | | |  | | | |  | | | | |
| ***PREGABALIN*** | |  | |  | |  |  |  |  |  |  |  |  |  | |  | |  | | |  | | | |  | | | |  | | | |  | | | |
| Montgomery 2013 | Pregabalin 200-600 mg/day | 528 | | 404 | | * | * | * | * | 66 | 40 | * | * | 25 | |  | | * | | | * | | | | * | | | | 29 | | | | Accidental injury=3; Diarrhea=10; Fatigue=21; Headache=28; Nausea=18 | | | |
| **OBSESSIVE COMPULSIVE DISORDER** | | | | | | | | | | | | | | | | | | | | | | | | | | | | | | | | | | | |  |
| **RANDOMISED DOUBLE-BLIND TRIALS** | | | | | |  |  |  |  |  |  |  |  |  | |  | |  | |  | | | |  | | | |  | | | |  | | | | |
| ***PREGABALIN*** | |  | |  | |  |  |  |  |  |  |  |  |  | |  | |  | | |  | | | |  | | | |  | | | |  | | | |
| Mowla 2020 | Pregabalin 75-225 mg/day (Adjunctive to Sertraline) | 28 | | * | | 0 | 0 | 0 | 0 | 1 | 4 | 0 | 0 | 0 | |  | | 0 | | | 0 | | | | 0 | | | | 0 | | | | Headache=1 | | | |
|  | Placebo (Adjunctive to Sertraline) | 28 | | * | | 0 | 0 | 0 | 0 | 0 | 1 | 0 | 0 | 0 | |  | | 0 | | | 0 | | | | 0 | | | | 0 | | | | Headache=1; Nausea=2 | | | |
| **OPEN LABEL TRIALS** | | |  | |  |  |  |  |  |  |  |  |  |  | |  | |  | |  | | | |  | | | |  | | | |  | | | | |
| ***PREGABALIN*** | |  | |  | |  |  |  |  |  |  |  |  |  | |  | |  | | |  | | | |  | | | |  | | | |  | | | |
| Oulis 2011 | Pregabalin 225-675 mg/day (Adjunctive to TAU) | 10 | | 8 | | 0 | 0 | 0 | 0 | 5 | 0 | 0 | 0 | 0 | |  | | 0 | | | 0 | | | | 0 | | | | 3 | | | | Fatigue=3 | | | |
| **POST TRAUMATIC STRESS DISORDER** | | | | | | | | | | | | | | | | | | | | | | | | | | | | | | | | | | | |  |
| **RANDOMISED DOUBLE-BLIND TRIALS** | | | | | |  |  |  |  |  |  |  |  |  | |  | |  | |  | | | |  | | | |  | | | |  | | | | |
| ***PREGABALIN*** | |  | |  | |  |  |  |  |  |  |  |  |  | |  | |  | | |  | | | |  | | | |  | | | |  | | | |
| Baniasadi 2014 | Pregabalin 300 mg/day | 18 | | * | | * | * | * | * | * | * | * | * | * | |  | | * | | | * | | | | * | | | | * | | | | * | | | |
|  | Placebo | 19 | | * | | * | * | * | * | * | * | * | * | * | |  | | * | | | * | | | | * | | | | * | | | | * | | | |
| **PRE-OPERATIVE ANXIETY** | | | | | | | | | | | | | | | | | | | | | | | | | | | | | | | | | | | |  |
| **RANDOMISED DOUBLE-BLIND TRIALS** | | | | | | |  |  |  |  |  |  |  |  | |  | |  | |  | | | |  | | | |  | | | |  | | | | |
| ***GABAPENTIN*** | |  | |  | |  |  |  |  |  |  |  |  |  | |  | |  | | |  | | | |  | | | |  | | | |  | | | |
| Abdel-Halim 2009 | Gabapentin 800 mg single dose | 20 | | * | | * | * | * | * | 0 | 2 | * | * | * | |  | | * | | | * | | | | * | | | | * | | | | Nausea/Vomiting=1 | | | |
|  | Dexamethasone IV 16 mg single dose | 20 | | * | | * | * | * | * | 0 | 0 | * | * | * | |  | | * | | | * | | | | * | | | | * | | | | Nausea/Vomiting=1 | | | |
|  | Gabapentin 800 mg + Dexamethasone IV 16 mg single dose | 20 | | * | | * | * | * | * | 0 | 2 | * | * | * | |  | | * | | | * | | | | * | | | | * | | | | * | | | |
|  | No intervention | 20 | | * | | * | * | * | * | 0 | 1 | * | * | * | |  | | * | | | * | | | | * | | | | * | | | | Nausea/Vomiting=1 | | | |
| Adam 2012 | Gabapentin 1200 mg single dose | 32 | | * | | * | * | * | * | * | * | * | * | * | |  | | * | | | * | | | | * | | | | * | | | | * | | | |
|  | Placebo | 32 | | * | | * | * | * | * | * | * | * | * | * | |  | | * | | | * | | | | * | | | | * | | | | * | | | |
| Bakry 2012 | Gabapentin 1200 mg single dose | 30 | | * | | * | * | * | * | * | * | * | * | * | |  | | * | | | * | | | | * | | | | * | | | | * | | | |
|  | Placebo | 30 | | * | | * | * | * | * | * | * | * | * | * | |  | | * | | | * | | | | * | | | | * | | | | * | | | |
| Clarke 2010 | Gabapentin 600 mg single dose | 22 | | * | | * | * | * | * | * | * | * | * | * | |  | | * | | | * | | | | * | | | | * | | | | * | | | |
|  | Placebo | 48 | | * | | * | * | * | * | * | * | * | * | * | |  | | * | | | * | | | | * | | | | * | | | | * | | | |
| Clarke 2013 | Gabapentin 1200 mg single dose | 25 | | * | | * | * | * | * | * | * | * | * | * | |  | | * | | | * | | | | * | | | | * | | | | * | | | |
|  | Placebo | 25 | | * | | * | * | * | * | * | * | * | * | * | |  | | * | | | * | | | | * | | | | * | | | | * | | | |
| Hoseini 2015 (IRCT2014041217231N1) | Gabapentin 600 mg single dose | 22 | | * | | * | * | * | * | * | * | * | * | * | |  | | * | | | * | | | | * | | | | * | | | | * | | | |
|  | Melatonin 6 mg single dose | 22 | | * | | * | * | * | * | * | * | * | * | * | |  | | * | | | * | | | | * | | | | * | | | | * | | | |
|  | Clonidine 0.2 mg single dose | 22 | | * | | * | * | * | * | * | * | * | * | * | |  | | * | | | * | | | | * | | | | * | | | | * | | | |
|  | Placebo | 22 | | * | | * | * | * | * | * | * | * | * | * | |  | | * | | | * | | | | * | | | | * | | | | * | | | |
| Joseph 2014 (CTRI/2010/091/002830) | Gabapentin 600 mg single dose | 25 | | * | | * | * | * | * | * | * | * | * | * | |  | | * | | | * | | | | * | | | | * | | | | * | | | |
|  | Alprazolam 0.5 mg single dose | 25 | | * | | * | * | * | * | * | * | * | * | * | |  | | * | | | * | | | | * | | | | * | | | | * | | | |
|  | Placebo | 25 | | * | | * | * | * | * | * | * | * | * | * | |  | | * | | | * | | | | * | | | | * | | | | * | | | |
| Khezri 2013 (NCT01200641) | Gabapentin 600 mg single dose | 40 | | * | | * | * | * | * | * | * | * | * | * | |  | | * | | | * | | | | * | | | | * | | | | * | | | |
|  | Melatonin 6 mg single dose | 40 | | * | | * | * | * | * | * | * | * | * | * | |  | | * | | | * | | | | * | | | | * | | | | * | | | |
|  | Placebo | 40 | | * | | * | * | * | * | * | * | * | * | * | |  | | * | | | * | | | | * | | | | * | | | | * | | | |
| Ménigaux 2005 | Gabapentin 1200 mg single dose | 20 | | 0 | | * | * | * | * | * | 0 | * | * | * | |  | | * | | | * | | | | * | | | | * | | | | * | | | |
|  | Placebo | 20 | | 0 | | * | * | * | * | * | 0 | * | * | * | |  | | * | | | * | | | | * | | | | * | | | | * | | | |
| Pathak 2014 | Gabapentin 1200 mg single dose | 40 | | * | | * | * | * | * | * | * | * | * | * | |  | | * | | | * | | | | * | | | | * | | | | * | | | |
|  | Placebo | 40 | | * | | * | * | * | * | * | * | * | * | * | |  | | * | | | * | | | | * | | | | * | | | | * | | | |
| Sava 2009 | Gabapentin 800 mg single dose | 25 | | * | | * | * | * | * | * | * | * | * | * | |  | | * | | | * | | | | * | | | | * | | | | * | | | |
|  | Placebo | 25 | | * | | * | * | * | * | * | * | * | * | * | |  | | * | | | * | | | | * | | | | * | | | | * | | | |
| Tirault 2010 | Gabapentin 1200 mg single dose | 70 | | * | | * | * | * | * | 1 | 5 | * | * | * | |  | | * | | | * | | | | 0 | | | | * | | | | * | | | |
|  | Hydroxyzine 75 mg single dose | 70 | | * | | * | * | * | * | 1 | 6 | * | * | * | |  | | * | | | * | | | | 0 | | | | * | | | | Headache=1 | | | |
|  | Placebo | 70 | | * | | * | * | * | * | 0 | 2 | * | * | * | |  | | * | | | * | | | | 1 | | | | * | | | | Headache=1; Nausea/Vomiting=1 | | | |
| ***PREGABALIN*** | |  | |  | |  |  |  |  |  |  |  |  |  | |  | |  | | |  | | | |  | | | |  | | | |  | | | |
| Gonano 2011 | Pregabalin 300 mg single dose | 20 | | 0 | | * | * | * | * | 0 | 0 | * | * | * | |  | | 0 | | | * | | | | 0 | | | | * | | | | * | | | |
|  | Placebo | 20 | | 0 | | * | * | * | * | 0 | 0 | * | * | * | |  | | 0 | | | * | | | | 0 | | | | * | | | | * | | | |
| Moreau-Bussiere 2013 (NCT01158859) | Pregabalin 150 mg single dose | 25 | | * | | * | * | * | * | * | * | * | * | * | |  | | * | | | * | | | | * | | | | * | | | | * | | | |
|  | Placebo | 25 | | * | | * | * | * | * | * | * | * | * | * | |  | | * | | | * | | | | * | | | | * | | | | * | | | |
| Nasr 2014 | Pregabalin 150 mg single dose | 20 | | * | | * | * | * | * | * | * | * | * | * | |  | | * | | | * | | | | * | | | | * | | | | * | | | |
|  | Melatonin 6 mg single dose | 20 | | * | | * | * | * | * | * | * | * | * | * | |  | | * | | | * | | | | * | | | | * | | | | * | | | |
| NCT00551135 2009 | Pregabalin 50 mg/day (two 25 mg doses) | 108 | | * | | * | * | * | * | * | * | * | * | * | |  | | * | | | * | | | | * | | | | * | | | |  | | | |
|  | Pregabalin 150 mg/day (two 75 mg doses) | 106 | | * | | * | * | * | * | * | * | * | * | * | |  | | * | | | * | | | | * | | | | * | | | |  | | | |
|  | Pregabalin 300 mg/day (two 150 mg doses) | 103 | | * | | * | * | * | * | * | * | * | * | * | |  | | * | | | * | | | | * | | | | * | | | |  | | | |
|  | Placebo | 108 | | * | | * | * | * | * | * | * | * | * | * | |  | | * | | | * | | | | * | | | | * | | | |  | | | |
| NCT00468845 2011 | Pregabalin 150 mg/day | 162 | | * | | * | * | * | * | * | * | * | * | * | |  | | * | | | * | | | | * | | | | * | | | | * | | | |
|  | Pregabalin 300 mg/day | 170 | | * | | * | * | * | * | * | * | * | * | * | |  | | * | | | * | | | | * | | | | * | | | | * | | | |
|  | Placebo | 169 | | * | | * | * | * | * | * | * | * | * | * | |  | | * | | | * | | | | * | | | | * | | | | * | | | |
| Nutt 2009 | Pregabalin 150 mg single dose | 27 | | * | | * | * | * | * | 6 | 3 | * | * | * | |  | | * | | | 0 | | | | * | | | | * | | | | Disturbance in attention=3; Fatigue=7 | | | |
|  | Alprazolam 0.5 mg single dose | 31 | | * | | * | * | * | * | 3 | 0 | * | * | * | |  | | * | | | 2 | | | | * | | | | * | | | | Disturbance in attention=1; Fatigue=7; Feeling abnormal=2 | | | |
|  | Placebo | 31 | | * | | * | * | * | * | 3 | 0 | * | * | * | |  | | * | | | 0 | | | | * | | | | * | | | | Fatigue=3 | | | |
| Roraruis 2004 | Gabapentin 1200 mg single dose | 38 | | 38 | | * | * | * | * | 6 | 14 | * | * | * | |  | | * | | | * | | | | 2 | | | |  | | | | Dry mouth=21; Headache=5 | | | |
|  | Oxezepam 15 mg single dose | 37 | | 37 | | * | * | * | * | 4 | 12 | * | * | * | |  | | * | | | * | | | | 1 | | | |  | | | | Dry mouth=15; Headache=5 | | | |
| Spreng 2011 | Pregabalin 150 mg single dose | 25 | | * | | * | * | * | * | * | * | * | * | * | |  | | * | | | * | | | | * | | | | * | | | | * | | | |
|  | Placebo | 25 | | * | | * | * | * | * | * | * | * | * | * | |  | | * | | | * | | | | * | | | | * | | | | * | | | |
| Shimoni 2016 | Pregabalin 150 mg single dose | 50 | | * | | * | * | * | * | 2 | 1 | * | * | * | |  | | * | | | * | | | | * | | | | * | | | | * | | | |
|  | Placebo | 50 | | * | | * | * | * | * | 0 | 2 | * | * | * | |  | | * | | | * | | | | * | | | | * | | | | * | | | |
| Singh 2019 | Pregabalin 150 mg single dose | 30 | | * | | * | * | * | * | * | * | * | * | * | |  | | * | | | * | | | | * | | | | * | | | | * | | | |
|  | Placebo | 30 | | * | | * | * | * | * | * | * | * | * | * | |  | | * | | | * | | | | * | | | | * | | | | * | | | |
| White 2009 | Pregabalin 75 mg single dose | 27 | | * | | * | * | * | * | * | * | * | * | * | |  | | * | | | * | | | | * | | | | * | | | | * | | | |
|  | Pregabalin 150 mg single dose | 27 | | * | | * | * | * | * | * | * | * | * | * | |  | | * | | | * | | | | * | | | | * | | | | * | | | |
|  | Pregabalin 300 mg single dose | 27 | | * | | * | * | * | * | * | * | * | * | * | |  | | * | | | * | | | | * | | | | * | | | | * | | | |
|  | Placebo | 27 | | * | | * | * | * | * | * | * | * | * | * | |  | | * | | | * | | | | * | | | | * | | | | * | | | |
| ***GABAPENTIN AND PREGABALIN*** | | | | | |  |  |  |  |  |  |  |  |  | |  | |  | |  | | | |  | | | |  | | | |  | | | | |
| Ghai 2012 | Gabapentin 900 mg single dose | 30 | | * | | * | * | * | * | * | * | * | * | * | |  | | * | | | * | | | | * | | | | * | | | | * | | | |
|  | Pregabalin 300 mg single dose | 30 | | * | | * | * | * | * | * | * | * | * | * | |  | | * | | | * | | | | * | | | | * | | | | * | | | |
|  | Placebo | 30 | | * | | * | * | * | * | * | * | * | * | * | |  | | * | | | * | | | | * | | | | * | | | | * | | | |
| * Side effect data is not reported/not reported in full e.g. only most frequent side effects (in more than 5 or 10% of patients) are reported and presence of other side effects are unknown.  † Authors contacted for data per arm, but no response. | | | | | | | | | | | | | | | | | | | | | | | | | | | | | | | | | | | |  |

# Risk of bias of included studies

## Figure 1. Risk of bias summary


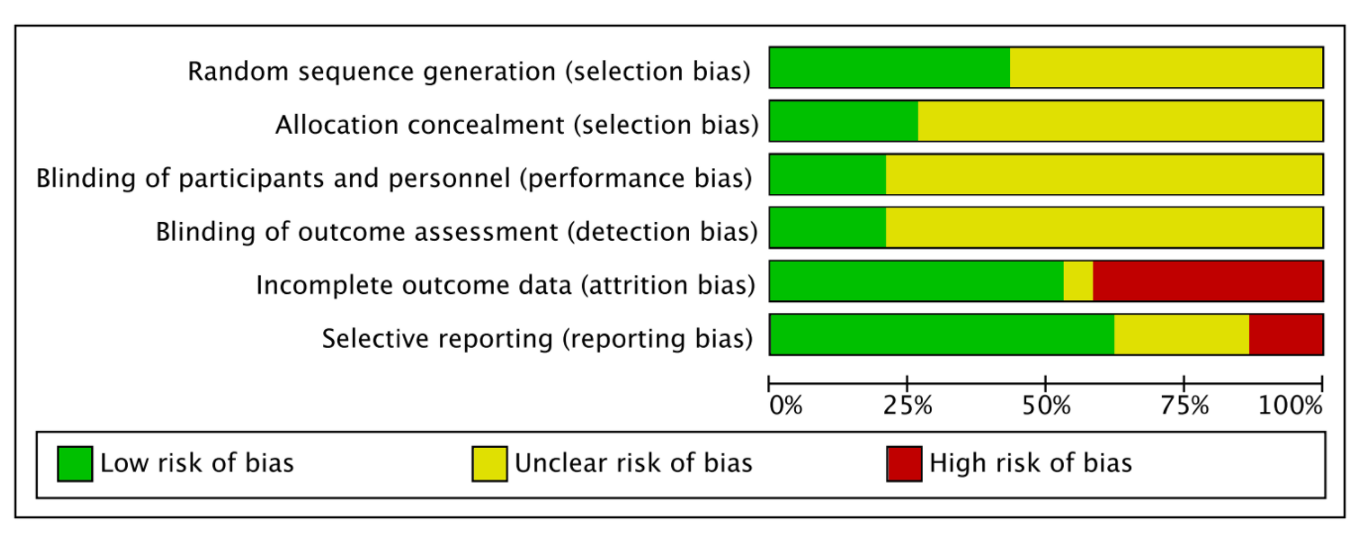


**Risk of bias, Figure 1 (above) and Figure 2 (next page).**

The Risk of Bias (RoB) assessment was done for all included double-blind RCTs using the 2011 Cochrane RoB tool. The majority of domains were rated as unclear risk of bias due to lack of sufficient details to warrant either a low or high risk of bias judgment. Notably, studies investigating bipolar disorder (BD) and generalised anxiety disorder (GAD) rated mostly as high risk of bias in the ‘incomplete outcome data’ domain, due to high rates of drop-out and the use of simple imputation methods (e.g. Last Observation Carried Forward, LOCF) for missing data.

## Figure 2. Risk of bias by individual study.

Bipolar disorder

-
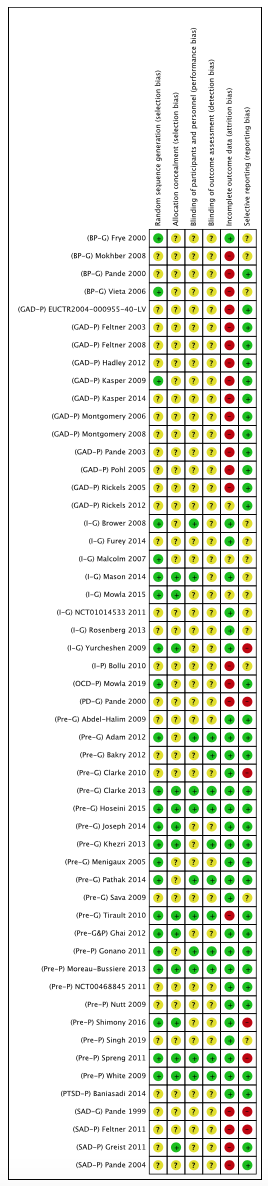
**BP-G:** Studies investigating the efficacy of gabapentin in bipolar disorder

Generalised anxiety disorder

- **GAD-P:** Studies investigating the efficacy of pregabalin in generalized anxiety disorder

Insomnia

- **I-G:** Studies investigating the efficacy of gabapentin in insomnia
- **I-P:** Studies investigating the efficacy of pregabalin in insomnia

Obsessive compulsive disorder

- **OCD-P:** Studies investigating the efficacy of pregabalin in obsessive compulsive disorder

Panic disorder

- **PD-G:** Studies investigating the efficacy of gabapentin in panic disorder

Preoperative anxiety

- **Pre-G:** Studies investigating the efficacy of gabapentin in preoperative anxiety
- **Pre-P**: Studies investigating the efficacy of pregabalin in preoperative anxiety
- **Pre-G&P:** Studies investigating both gabapentin and pregabalin for preoperative anxiety

Posttraumatic stress disorder

- **PTSD-P**: Studies investigating the efficacy of pregabalin in posttraumatic stress disorder

Social anxiety disorder

- **SAD-G:** Studies investigating the efficacy of gabapentin in social anxiety disorder
- **SAD-P:** Studies investigating the efficacy of pregabalin in social anxiety disorder

##
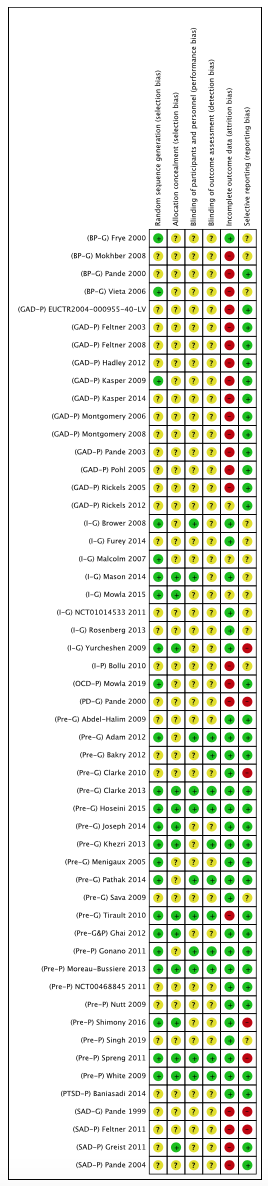


# Primary outcome: efficacy, forest plots, including sensitivity analyses

## Figure 3: Pregabalin vs placebo in a meta-analysis of generalised anxiety disorder, social anxiety disorder, pre-operative anxiety, PTSD, and OCD


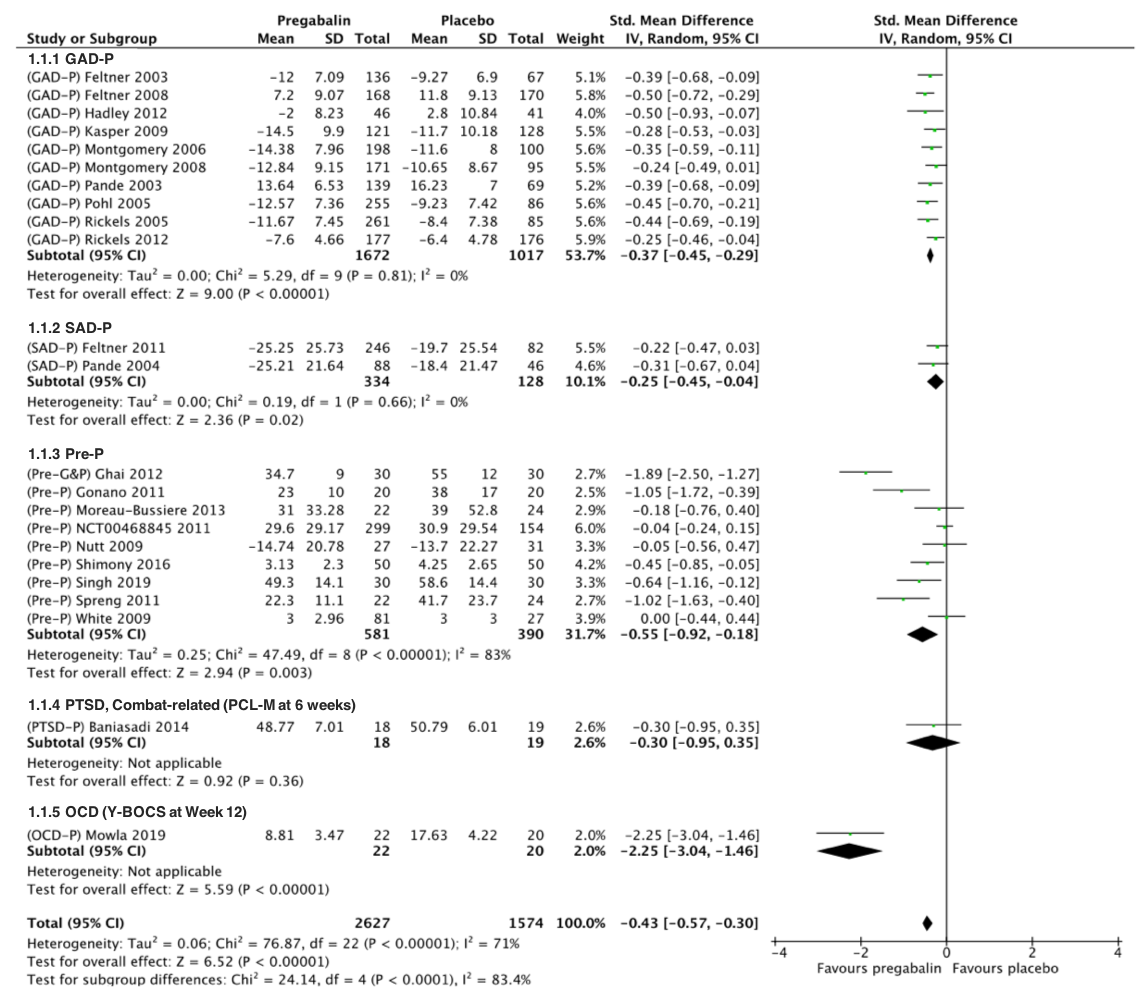


**Figure 3.1 is a random-effects meta-analysis of all studies assessing the efficacy of pregabalin versus placebo in anxiety disorders. The effect estimate is in standardised mean difference (SMD). The outcome measures are detailed below.**

**GAD-P**: Meta-analysis of double-blind RCTs (DB-RCTs) testing pregabalin versus placebo in generalised anxiety disorder (GAD), using baseline-to-endpoint change in total HAM-A score or (in Feltner 2008) endpoint HAM-A score.

**SAD-P:** Meta-analysis of DB-RCTs testing pregabalin versus placebo in social anxiety disorder (SAD), using baseline-to-endpoint change in primary outcome measure (Liebowitz Social Anxiety Scale, LSAS).

**Pre-P:** Meta-analysis of DB-RCTs testing pregabalin versus placebo in pre-operative anxiety.

**PTSD-P:** DB-RCT of pregabalin versus placebo (Baniasadi 2014) in chronic PTSD (combat-related) patients using PCL-M at 6 weeks from baseline.

**OCD-P:** DB-RCT of pregabalin versus placebo (Mowla 2020) in SSRI-resistant OCD using Y-BOCS at 12 weeks from baseline.

## Figure 4: Gabapentin vs placebo in a meta-analysis of pre-operative anxiety, social anxiety disorder, and panic disorder


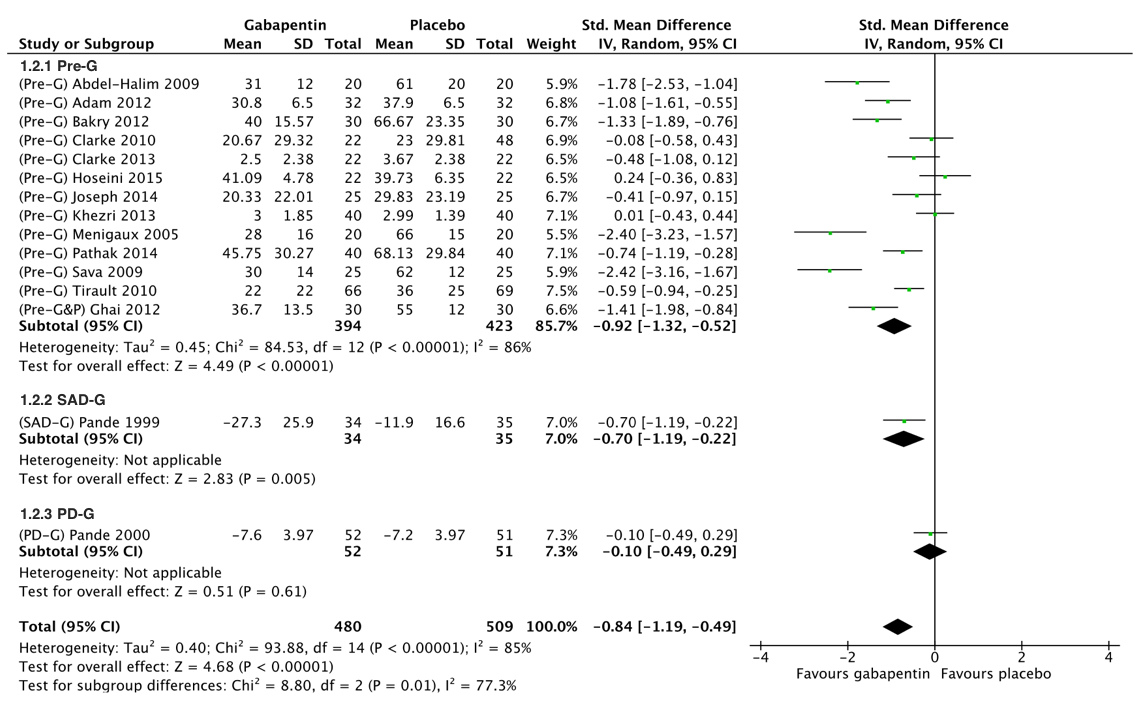


**Random-effects meta-analysis of all studies assessing the efficacy of gabapentin versus placebo in anxiety disorders. The effect estimate is in standardised mean difference (SMD). The outcome measures are detailed below.**

**Pre-G:** Meta-analysis of DB-RCTs testing gabapentin versus placebo in pre-operative anxiety

**SAD-G:** DB-RCT testing gabapentin versus placebo in social anxiety disorder (Pande 1999), using baseline-to-endpoint change in primary outcome measure (Liebowitz Social Anxiety Scale, LSAS).

**PD-G:** DB-RCT testing gabapentin versus placebo in panic disorder (Pande 2000)

## Figure 5: Pregabalin vs placebo in generalised anxiety disorder, Mean difference in HAM-A*


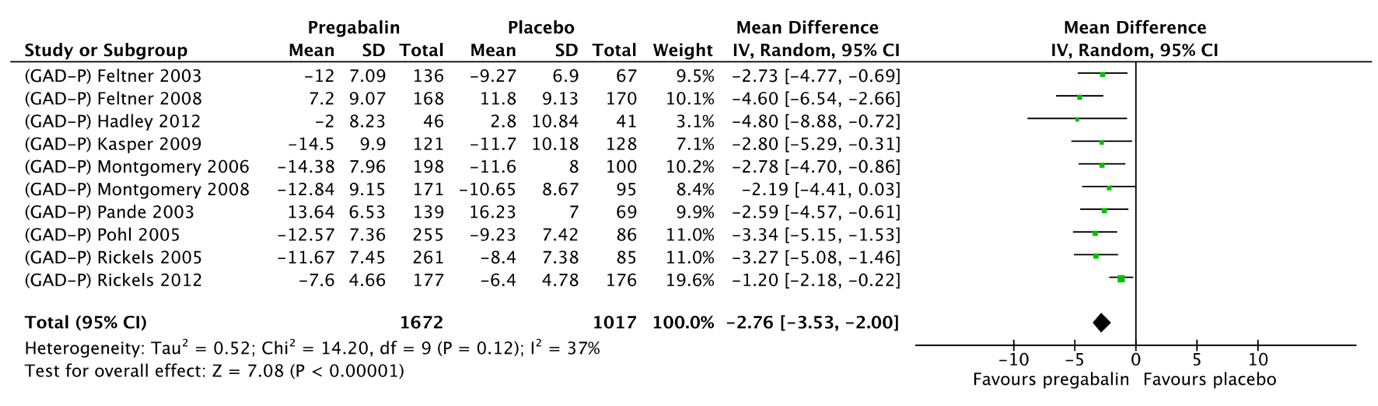


**Random-effects meta-analyses of the efficacy of pregabalin, or another active compound, versus placebo in generalized anxiety disorder. The effect estimate is mean difference (MD) in HAM-A between the treatment and placebo groups. *HAM-A, Hamilton Anxiety Rating Scale**

## Figure 6: Funnel Plot - Pregabalin vs placebo in generalised anxiety disorder (GAD), Mean difference in HAM-A


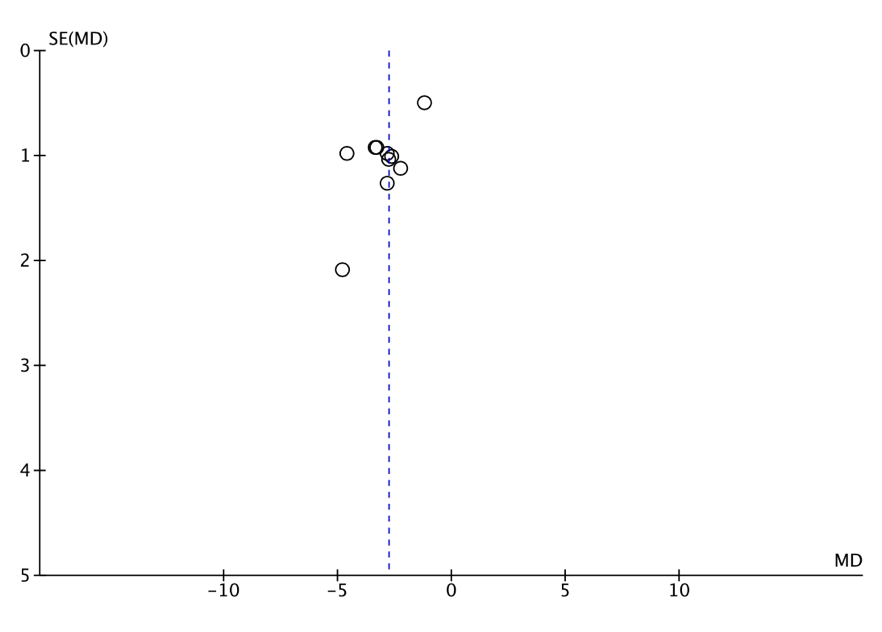


A funnel plot was made for studies investigating the efficacy of pregabalin versus placebo in GAD. X-axis: Effect estimate, Mean difference (MD) in HAM-A. Y-axis: Standard error (SE) of the MD. Each dot represents a single study. There was no obvious funnel plot asymmetry.

HAM-A, Hamilton Anxiety Rating Scale.

## Figure 7: Pregabalin vs lorazepam in generalised anxiety disorder, Mean difference in HAM-A


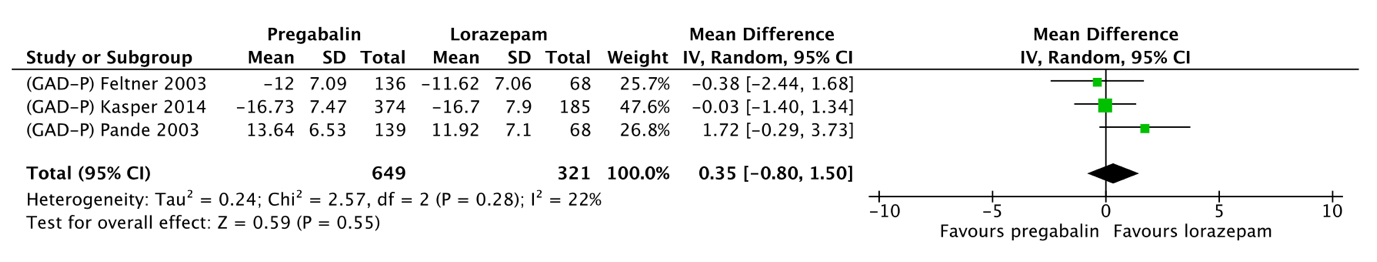


## Figure 8: Lorazepam vs placebo in generalised anxiety disorder, Mean difference in HAM-A


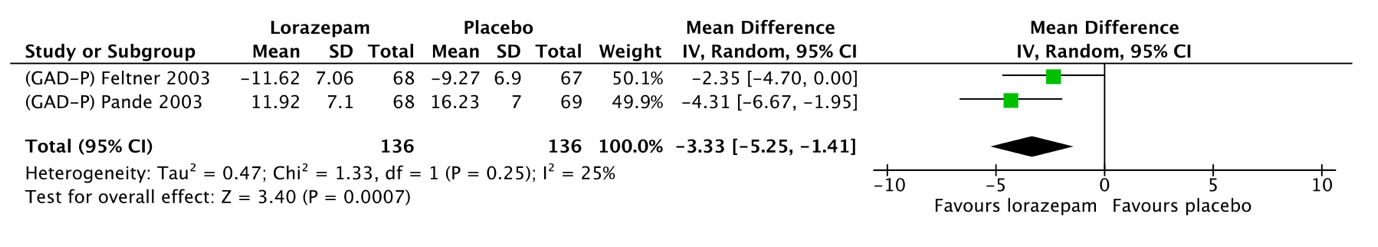


## Figure 9: Pregabalin vs venlafaxine in generalised anxiety disorder, Mean difference in HAM-A


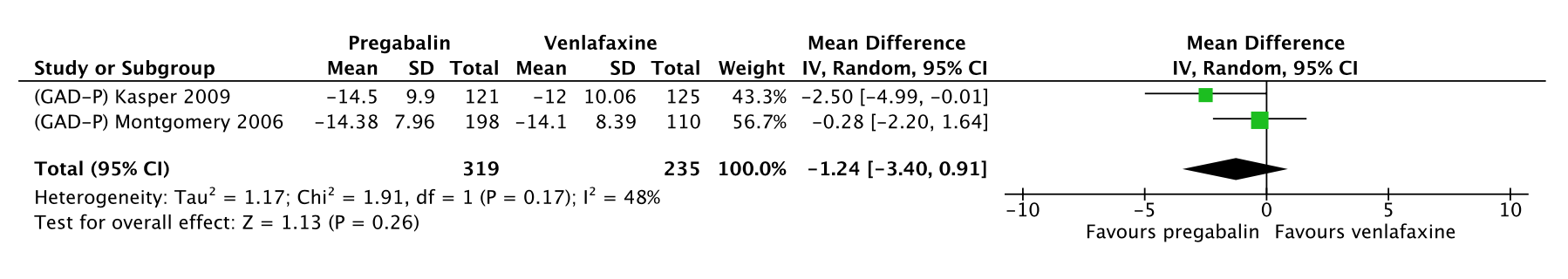


## Figure 10: Venlafaxine vs placebo in generalised anxiety disorder, Mean difference in HAM-A


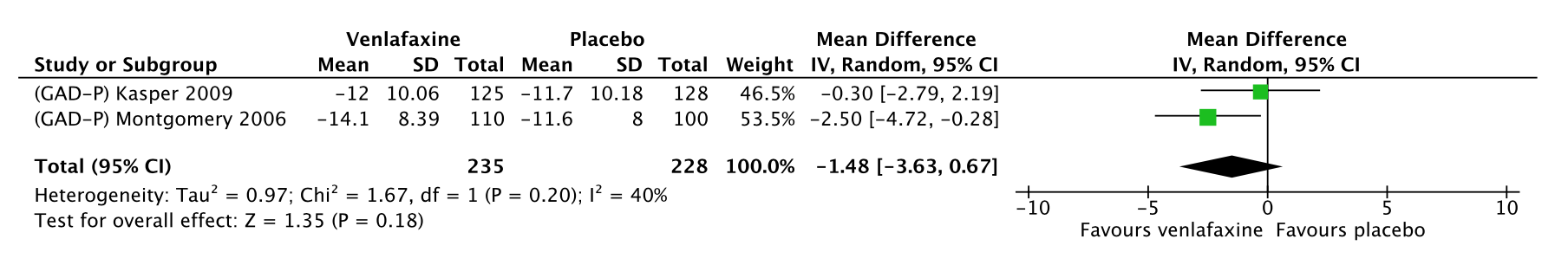


## Figure 11: Funnel plot (gabapentin versus placebo in preoperative anxiety)


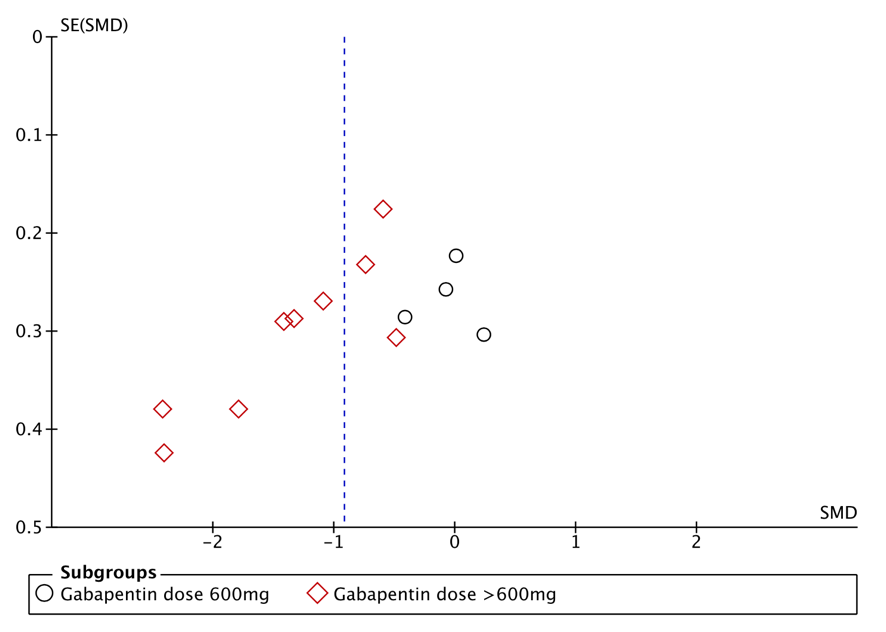


A funnel plot was made for studies investigating the efficacy of gabapentin versus placebo in preoperative anxiety. X-axis: treatment effect estimate in standardised mean difference (SMD). Y-axis: standard error of the SMD. Each dot represents a single study, with red square representing studies investigating gabapentin at a dose above 600mg (k=9 studies), and black circles representing studies investigating gabapentin dose of 600mg (k=4). The funnel plot suggests possible small study effects, with greater effect size being observed in high-dose gabapentin studies with smaller samples. This should be interpreted with caution due to potential confounding by the high degree of I^2^ heterogeneity that was observed in the high-dose group and lack of formal statistical assessment.

## Figure 12: Gabapentin vs placebo in insomnia participants with alcohol dependence, Week 6, SMD (standardised mean difference)


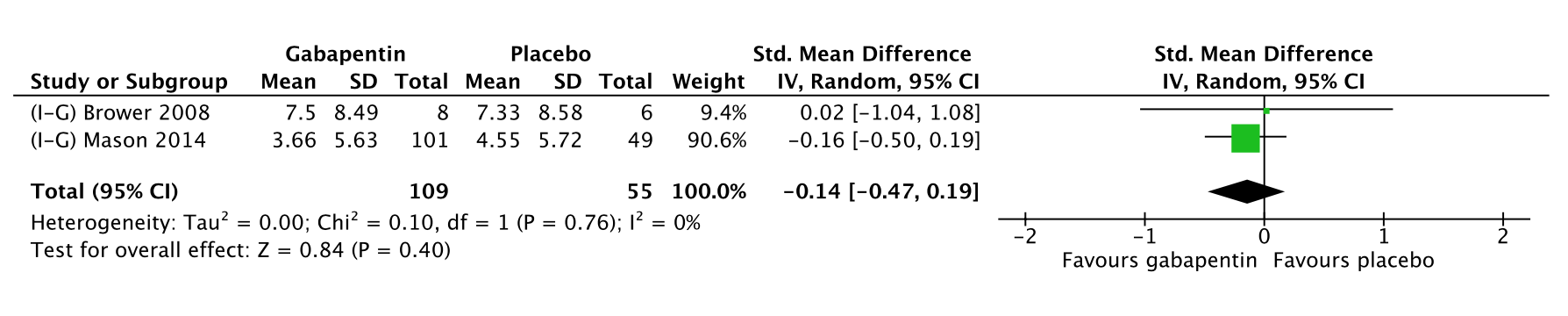


## Figure 13: Gabapentin vs placebo in insomnia participants with alcohol dependence, Week 12, SMD (standardised mean difference)


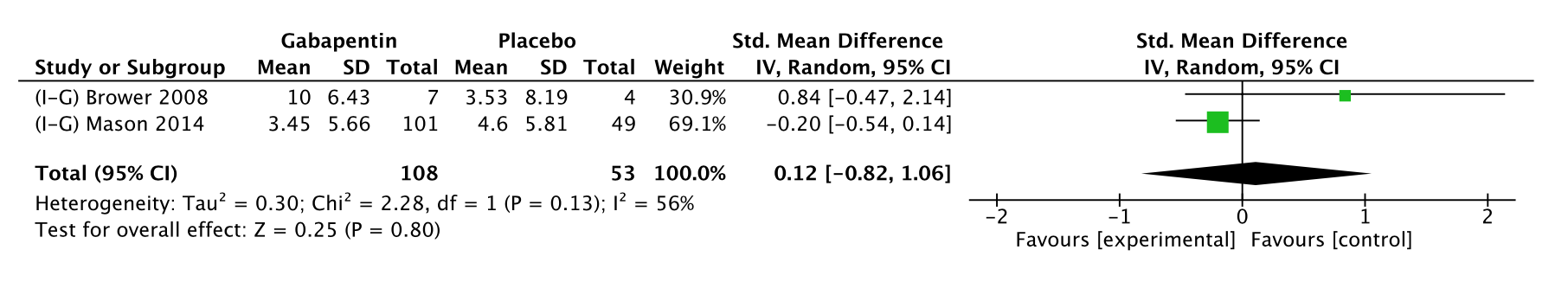


## Figure 14: Gabapentin vs placebo, weeks 4-6, subjective measures, including healthy volunteers, SMD (standardised mean difference)


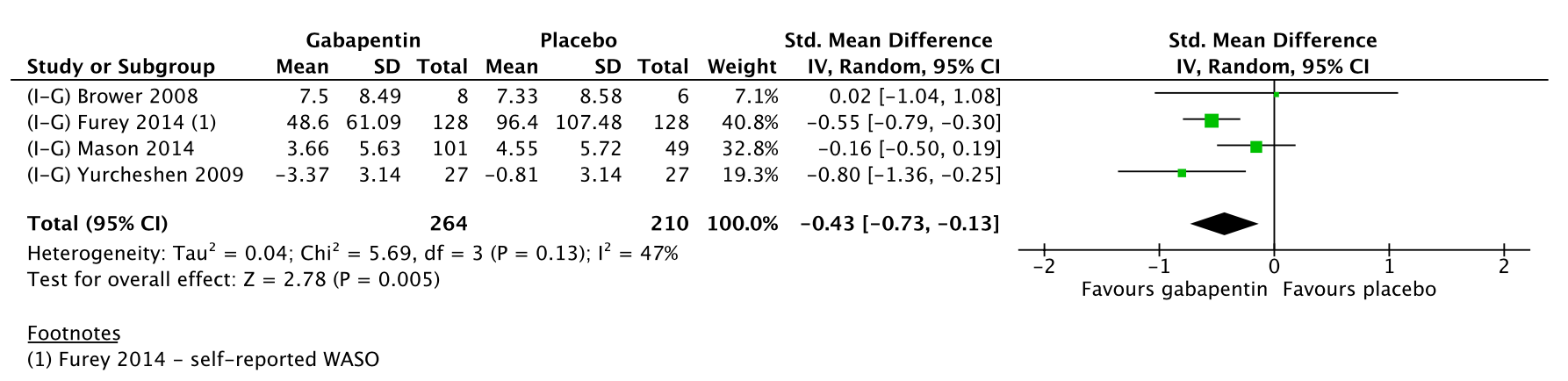


## Figure 15: Gabapentin vs placebo, week 12, subjective measures, including healthy volunteers, SMD (standardised mean difference)


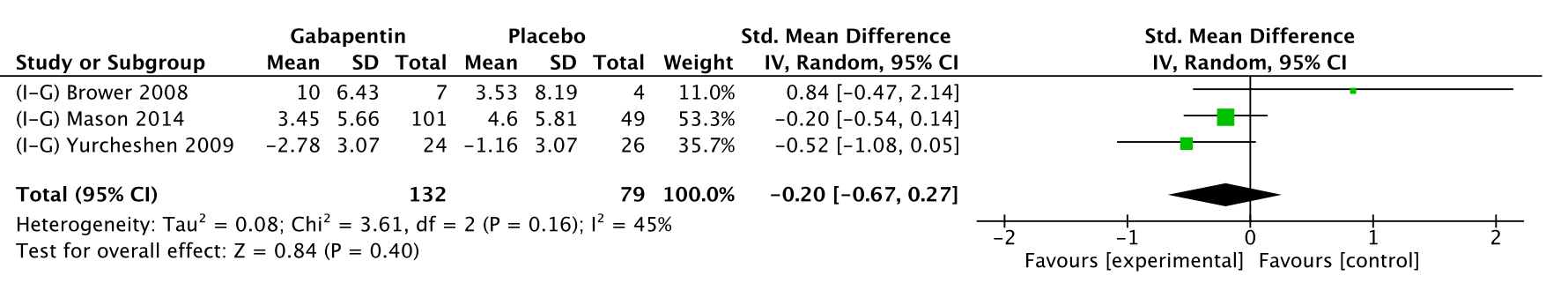


## Figure 16: Gabapentin vs placebo, weeks 1-4, Mean difference in PSG-derived WASO*, including healthy volunteers


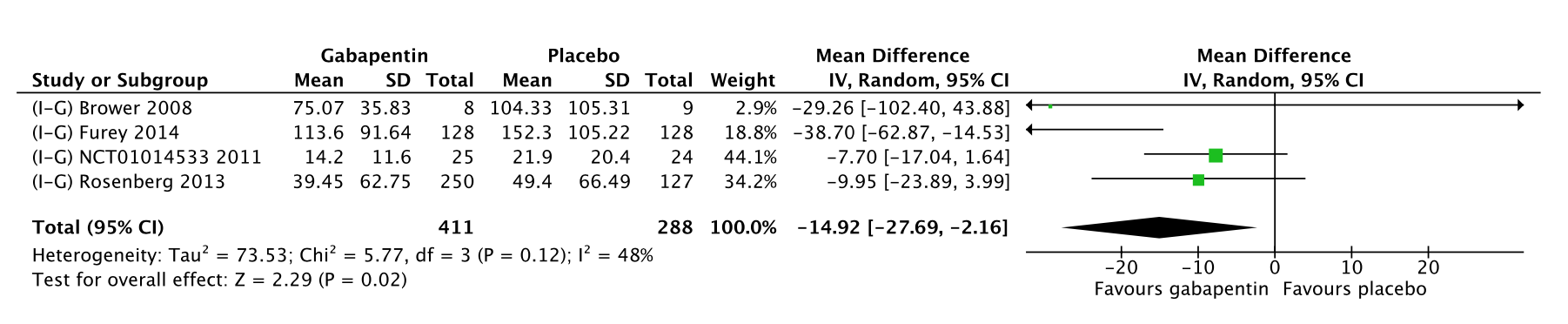


*PSG-derived WASO: polysomnography-derived wake after sleep onset

## Figure 17: Pregabalin vs placebo in social anxiety disorder, Mean difference in LSAS*


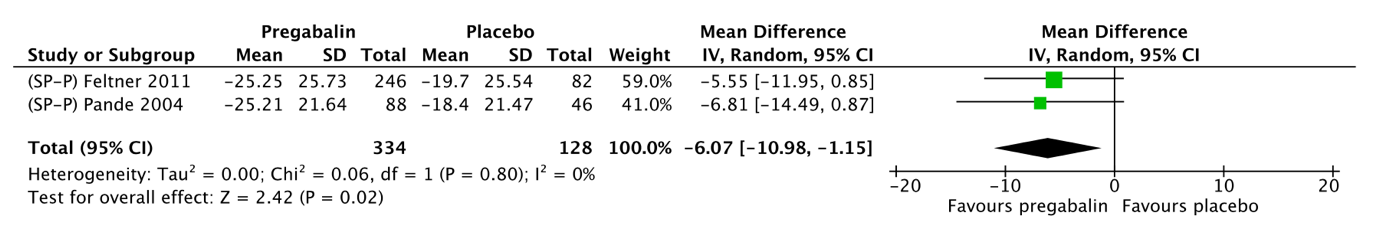


*LSAS: Liebowitz Social Anxiety Scale

## Figure 18: Pregabalin vs placebo in social anxiety disorder, Mean difference in HAM-A*


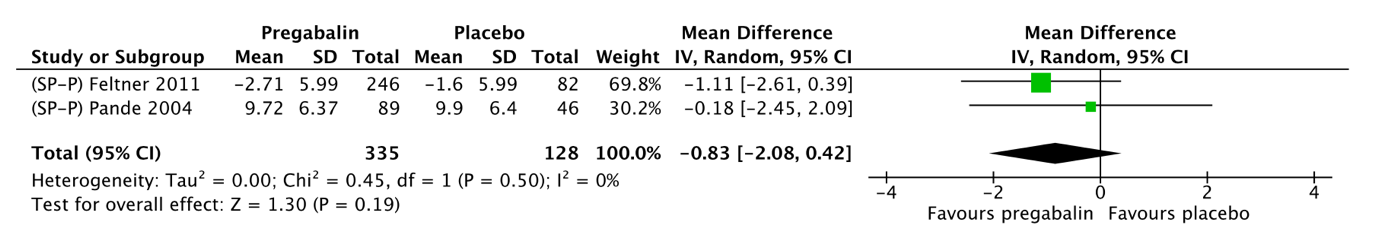


*HAM-A: Hamilton Anxiety Rating Scale

## Figure 19: Pregabalin vs placebo in social anxiety disorder, Mean difference in HAM-D*


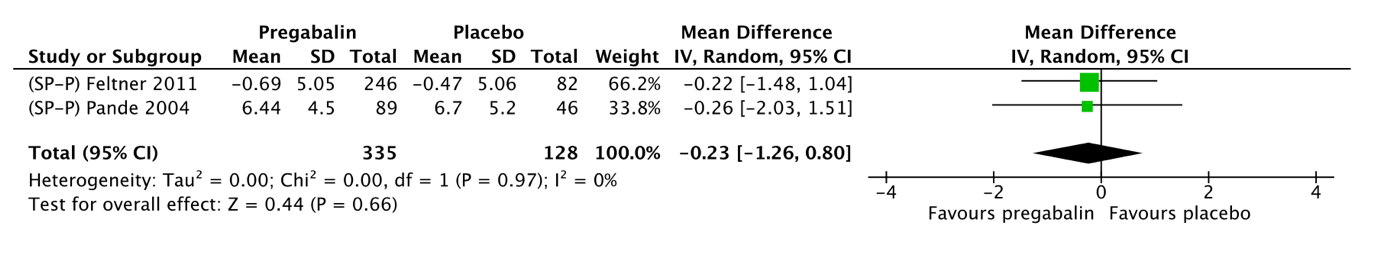


*HAM-D: Hamilton Rating Scale for Depression

## Figure 20: Pregabalin vs placebo in social anxiety disorder, Mean difference in MFQ*


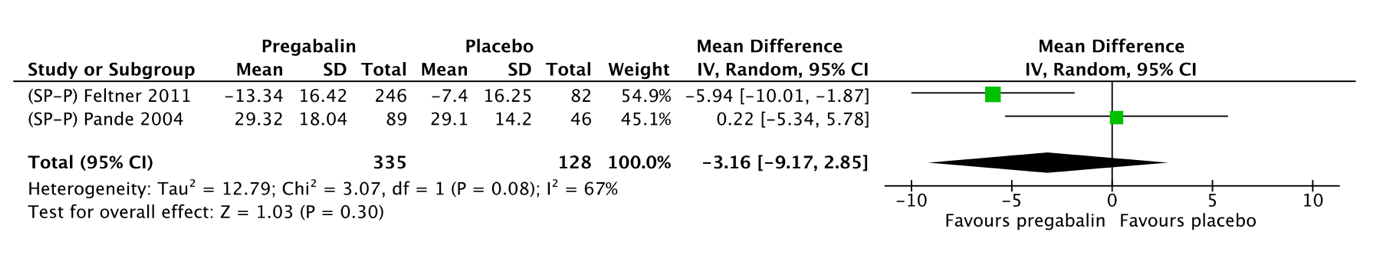


*MFQ: Marks Fear Questionnaire

# Secondary outcome: acceptability, forest plots

## Figure 21: Acceptability of pregabalin versus placebo in GAD


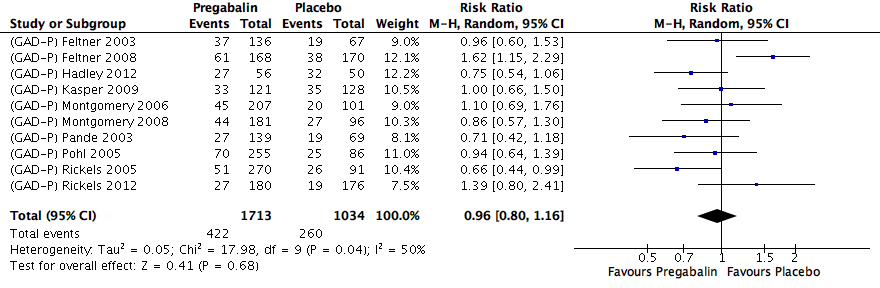

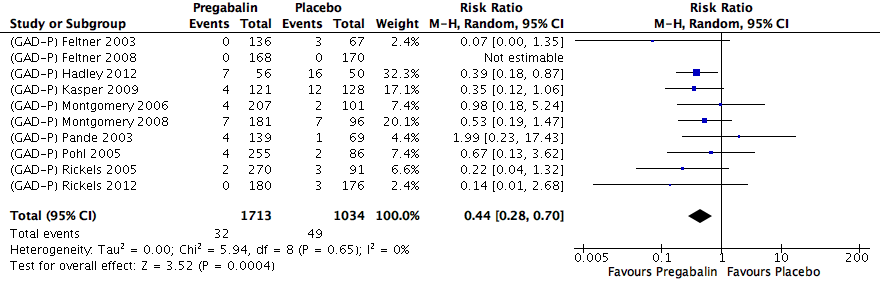

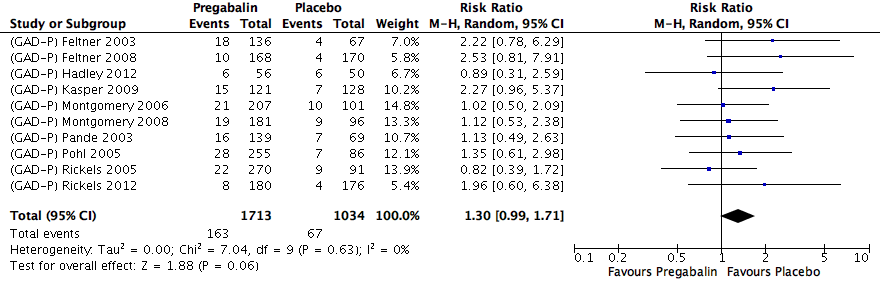


**A. All cause dropout**

**B. Dropout due to inefficacy**

**C. Dropout due to adverse events**

## Figure 22: Acceptability of pregabalin versus placebo in SAD

**A. All cause dropout**

**B. Dropout due to inefficacy**

**C. Dropout due to adverse events**


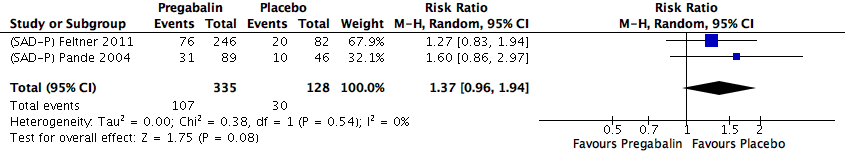

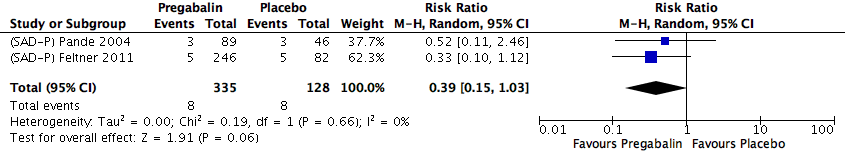

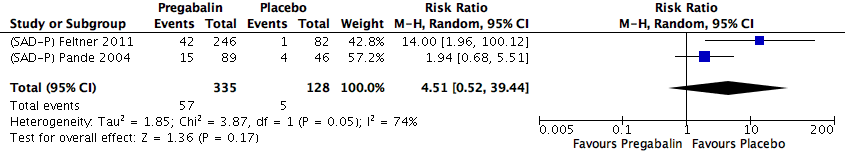


## Supplementary Analyses

**GAD**

Three of the 11 DB-RCTs assessing pregabalin in GAD included a lorazepam arm (Feltner 2003; Kasper 2014; Pande 2003). Two of these studies reported acceptability outcomes and were subsequently compared in a random effects meta-analysis. Compared to lorazepam, pregabalin showed reduced risk of all cause dropout (risk ratio [RR]=0.53, 95% CI=0.40-0.71, *p*<0.001), and dropouts due to adverse events (RR=0.39, 95% CI=0.26-0.58, *p*<0.001), however there was no difference in their risk of dropout due to inefficacy (RR=0.76, 95% CI=0.07-7.94, *p*=0.82) (see Supplementary Figure 3.1-3.3).

Two of the 11 DB-RCTs assessing pregabalin in GAD included a venlafaxine arm (Kasper 2009; Montgomery 2006). A random effects meta-analysis of acceptability outcomes in these studies did not reveal significant differences between pregabalin and venlafaxine in the risk of all cause dropout (RR=0.77, 95% CI=0.59-1.02, *p*=0.06) or dropouts due to inefficacy (RR=1.28, 95% CI=0.40-4.05, *p*=0.68), however pregabalin showed a significant reduction in risk of dropout due to adverse events (RR=0.58, 95% CI=0.39-0.87, *p*=0.009). Fixed effects analyses did not significantly alter effect estimates for any of the aforementioned acceptability outcomes.

## Figure 23: Acceptability of pregabalin versus lorazepam in GAD

**A. All cause dropout**

**B. Dropout due to inefficacy**

**C. Dropout due to adverse events**


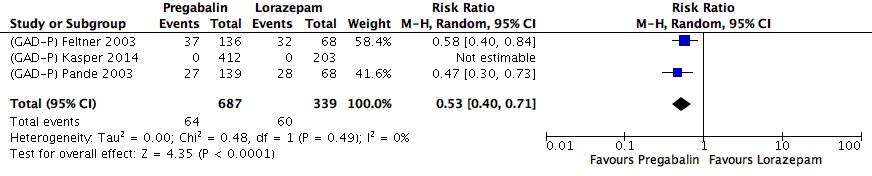

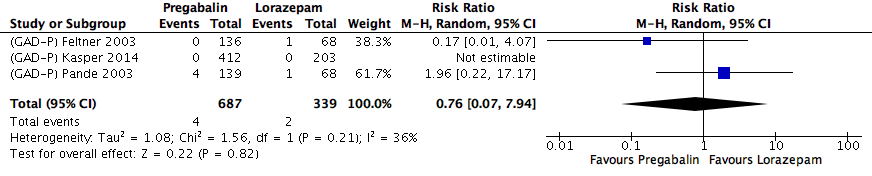

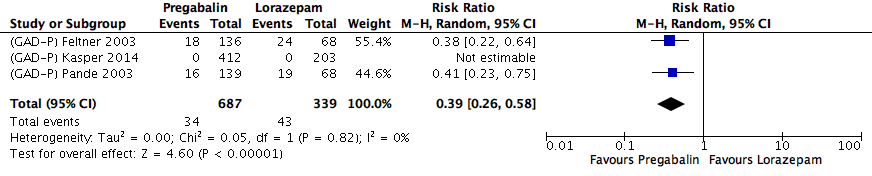


## Figure 24: Acceptability of pregabalin versus venlafaxine in GAD

**A. All cause dropout**

**B. Dropout due to inefficacy**

**C. Dropout due to adverse events**


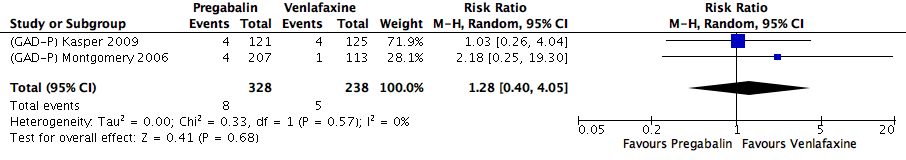

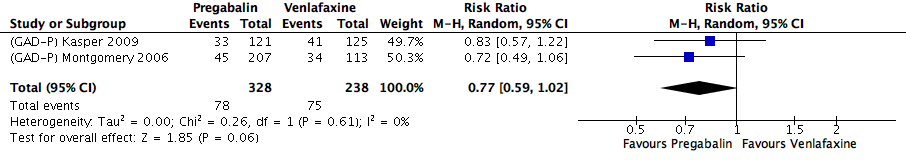

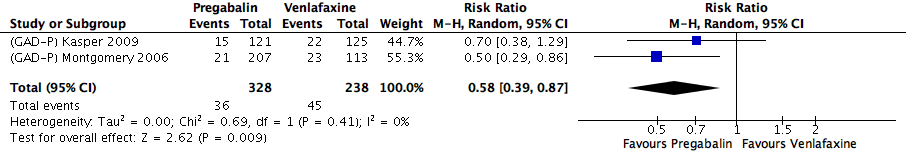


## Figure 25: Acceptability of gabapentin versus placebo in participants with alcohol dependence and related sleep disturbance

**All cause dropout**


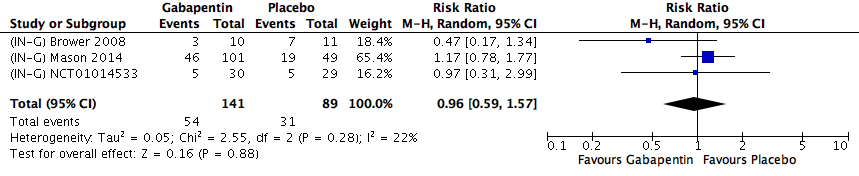


# **Further details of tolerability data**

## Bipolar disorder (BD)

In addition to double-blind RCTs, 11 open-label studies were included for analysis of tolerability, 8 of which reported outcome data.

The following seven open-label studies assessed gabapentin as an adjunct to mood stabiliser or benzodiazepine treatment (total patients *n*=144): Altshuler et al., 1999; Knoll et al., 1998; McElroy et al., 1997; Perugi et al., 1999; Wang et al., 2002; Young et al., 1997; Young et al., 1999. Gabapentin was most commonly associated with sedation (39/144, 27%), movement disorders (ataxia/tremor) (17/144, 12%) and gastrointestinal upset (17/144, 12%). In addition, sleep problems, drowsiness, fatigue and cognitive side-effects (forgetfulness/memory loss, impaired cognition) were reported across studies.

One study assessed pregabalin as an adjunct to standard treatments for BD (*n*=58, Schaffer et al., 2012). This study reported adverse events including thought overactivation (12/58, 21%), weight gain (8/58, 14%) and increased appetite (8/58, 14%).

## Generalised anxiety disorder (GAD) and Social anxiety disorder (SAD)

12 monotherapy DB-RCTs (Feltner et al., 2003; Feltner et al., 2008; Feltner et al., 2011; Griest et al., 2011; Hadley et al., 2012; Kasper et al., 2009; Montgomery et al., 2006; Montgomery et al., 2008; Pande et al., 2003; Pande et al., 2004; Pohl et al., 2005; Rickels et al., 2005), 1 DB-RCT adjunctive to SSRI/SNRI treatment (Rickels et al., 2012), 1 open-label, randomised study (Cvjetkovic-Bosnjak et al., 2015) and 1 open-label non-randomised study (Montgomery et al., 2013). Of 2,703 patients treated with pregabalin across these studies, 631 (23.3%) reported experiencing dizziness, 541 (20%) drowsiness, 235 (8.7%) headache, 223 (8.3%) dry mouth, 204 (7.5%) infection, 187 (6.9%) nausea, 135 (5%) fatigue, 94 (3.5%) visual disturbance, 81 (3%) thinking abnormally, 78 (2.9%) insomnia, 68 (2.5%) weight gain, 52 (1.9%) constipation and 48 (1.8%) diarrhoea. Other less commonly reported side-effects included vertigo, movement disorders, vomiting and incoordination. In studies with multiple dose arms, it was observed that more side-effects were reported with increasing pregabalin dose, as might be expected (e.g. Feltner et al., 2003; Feltner et al., 2011, Montgomery et al., 2006; Pande et al., 2003; Pande et al., 2004; Rickels et al., 2005). Only one DB-RCT study assessed gabapentin in SAD (Pande et al., 1999), with tolerability data showing that side effects included infection, dizziness, headache, drowsiness and fatigue.

## Preoperative anxiety

Gabapentin was associated with drowsiness in 3 DB-RCTs in preoperative anxiety (23/148, 16% patients randomised to gabapentin arms) (Abdel-Halim et al., 2009; Rorarius et al., 2004; Tirault et al., 2010). Rorarius et al., (2004) further reported side effects of dry mouth (21/38, 55%), dizziness (6/38, 16%), headache (5/38, 13%) and visual disturbance (2/38, 5%) in gabapentin treated patients, although similar incidence of side-effects were experienced in the comparison oxepam arm of the study. Only 1 DB-RCT of pregabalin in preoperative anxiety reported tolerability data (Nutt et al., 2009), with reported side effects including fatigue (7/27, 26%) dizziness (6/27, 22%), disturbances in attention (3/27, 11%) and drowsiness (3/27, 11%).

## Insomnia

Nine studies were included to examine the tolerability of gabapentin in insomnia/sleep disturbance, of which 7 reported side-effect outcomes; 6 DB-RCTs (Brower et al., 2008; Furey et al., 2014; Mason et al., 2014; NCT01014533, Rosenburg et al., 2013; Yurchesen et al., 2009) and 1 open-label, non-randomised study (NCT02040532). Of 575 patients treated with gabapentin across these studies, 66 (11%) reported experiencing at least 1 side effect. The most commonly reported across studies were headache (32/66, 48% of patients), fatigue (27/66, 41%), drowsiness (21/66, 32%), dizziness (8/66, 12%) and nausea (5/66, 8%) although it is unclear whether these could be attributed to the condition being studied. Tolerability of pregabalin in insomnia/sleep disturbance could not be examined due to side-effect data not being reported.

# Changes to original protocol

Post-hoc subgroup analyses using empirically guided dose thresholds were conducted to investigate the large between-study heterogeneity observed in preoperative anxiety studies. See Methods section of the main text for details.

Additional sensitivity analyses (e.g. studies originally reporting data in median/IQR/range requiring conversion to mean/SD values) were conducted due to concerns regarding the impact of such factors on the treatment effect estimate. See Methods section of the main text.
